# An evidence-based methodology for systematic evaluation of clinical outcome assessment measures for traumatic brain injury

**DOI:** 10.1371/journal.pone.0242811

**Published:** 2020-12-14

**Authors:** Andrea N. Christoforou, Melissa J. Armstrong, Michael J. G. Bergin, Ann Robbins, Shannon A. Merillat, Patricia Erwin, Thomas S. D. Getchius, Michael McCrea, Amy J. Markowitz, Geoffrey T. Manley, Joseph T. Giacino

**Affiliations:** 1 Department of Physical Medicine and Rehabilitation, Spaulding Rehabilitation Hospital, Charlestown, Massachusetts, United States of America; 2 Department of Physical Medicine and Rehabilitation, Harvard Medical School, Boston, Massachusetts, United States of America; 3 Department of Neurology, College of Medicine, University of Florida, Gainesville, Florida, United States of America; 4 Ann Robbins LLC, Bradenton, Florida, United States of America; 5 American Academy of Neurology, Minneapolis, Minnesota, United States of America; 6 Mayo Clinic, Rochester, Minnesota, United States of America; 7 American Heart Association/American College of Cardiology, Dallas, Texas, United States of America; 8 Department of Neurosurgery, Medical College of Wisconsin, Milwaukee, Wisconsin, United States of America; 9 Brain and Spinal Injury Center, University of California, San Francisco, San Francisco, California, United States of America; 10 Department of Neurosurgery, University of California, San Francisco, San Francisco, California, United States of America; University of Palermo, ITALY

## Abstract

**Introduction:**

The high failure rate of clinical trials in traumatic brain injury (TBI) may be attributable, in part, to the use of untested or insensitive measurement instruments. Of more than 1,000 clinical outcome assessment measures (COAs) for TBI, few have been systematically vetted to determine their performance within specific “contexts of use (COU).” As described in guidance issued by the U.S. Food and Drug Administration (FDA), the COU specifies the population of interest and the purpose for which the COA will be employed. COAs are commonly used for screening, diagnostic categorization, outcome prediction, and establishing treatment effectiveness. COA selection typically relies on expert consensus; there is no established methodology to match the appropriateness of a particular COA to a specific COU. We developed and pilot-tested the *E*vidence-*B*ased *C*linical *O*utcome assessment *P*latform (EB-COP) to systematically and transparently evaluate the suitability of TBI COAs for specific purposes.

**Methods and findings:**

Following a review of existing literature and published guidelines on psychometric standards for COAs, we developed a 6-step, semi-automated, evidence-based assessment platform to grade COA performance for six specific purposes: diagnosis, symptom detection, prognosis, natural history, subgroup stratification and treatment effectiveness. Mandatory quality indicators (QIs) were identified for each purpose using a modified Delphi consensus-building process. The EB-COP framework was incorporated into a Qualtrics software platform and pilot-tested on the Glasgow Outcome Scale—Extended (GOSE), the most widely-used COA in TBI clinical studies.

**Conclusion:**

The EB-COP provides a systematic methodology for conducting more precise, evidence-based assessment of COAs by evaluating performance within specific COUs. The EB-COP platform was shown to be feasible when applied to a TBI COA frequently used to detect treatment effects and can be modified to address other populations and COUs. Additional testing and validation of the EB-COP are warranted.

## Introduction

Every year, over 50 million people worldwide sustain traumatic brain injury (TBI). The global burden of TBI is enormous, dramatically impacting economic, societal, professional and personal welfare. Apart from the direct effects, which are often life-changing, there is growing evidence that TBI is a risk factor for development of major co-morbidities and neurodegenerative diseases [1]. As such, TBI has become a leading cause of death and disability globally across all ages, costing approximately $400 billion annually [2]. Despite decades of large-scale, government-funded research, effective treatments focused on neuroprotection and acceleration of recovery after TBI remain elusive. A recent systematic review of randomized controlled trials in acute moderate-to-severe TBI identified 191 completed trials, of which only 26 were judged as high-quality and with sufficient sample sizes. Of these, only three showed a statistically significant positive effect and none resulted in clearly discernible clinical benefit. The authors concluded that considerable investment of resources had resulted in very little clinically translatable evidence [3].

There is increasing concern that the use of insensitive, mis-matched, or psychometrically unproven clinical outcome assessment (COAs) measures in drug and device trials may be contributing to the failure to develop effective therapies for TBI [4–6]. Of the nearly 1000 COAs that have been employed in TBI research [2], including those that yield a quantitative score, most rely on subjective ratings made by either the examiner or respondent. This approach can increase error variance, introduce bias and compromise reproducibility. A second concern is that many COAs are not adequately responsive to important changes in function, including their ability to effectively measure response to treatment, which may predispose TBI clinical trials to false negative results [7]. Perhaps most importantly, very few COAs have been systematically vetted to assess their performance within well-defined contexts of use (COU), whether in TBI or other neurologic disorders [8]. The U.S. Food and Drug Administration (FDA) utilizes this concept in assessing whether a particular outcome measure is appropriate for use in a well-controlled clinical trial, as described in their guidance documents [8–13] on patient reported outcomes and drug development tools. At a minimum, the COU specifies, (1) the characteristics of the disease and the intended population to be tested and (2) the intended purpose of the COA (e.g., diagnostic categorization, outcome prediction). Each specific COU for a COA must meet a different set of performance criteria as a selected instrument may be valid and reliable when used for one purpose or population but not another [14].

The Glasgow Outcome Scale-Extended (GOSE) [15] is the most widely-used COA in TBI clinical studies [16] and in many TBI drug and device trials submitted to FDA. The scale was originally developed to assess global disability and recovery following TBI. Ratings are obtained through a structured interview and outcomes categorized into eight levels of function ranging from dead to good recovery. While the GOSE has demonstrated its utility in generally describing level of global functional recovery after TBI [16], its appropriateness for use in detecting therapeutic effectiveness has never be evaluated. This knowledge gap is amplified by the broad range of function subsumed by a single GOSE outcome category. For example, 20–30% of patients who fall into the highest level of the GOSE (ie, Level 8: Upper Good Recovery) have been shown to have persistent injury-related cognitive and psychological health problems when more specialized measures are administered [17]. On the other end of the severity spectrum, the GOSE’s Lower Severe Disability category (Level 3) lumps together patients who can live unsupervised at home for less than 8 hours a day with those whose only sign of consciousness is visual pursuit. These observations suggest that the GOSE may not be able to capture natural history or treatment-related changes at the upper and lower limits of the TBI continuum.

Several research funding agencies, including the National Institute of Neurological Disorders and Stroke (NINDS), Department of Defense (DOD), and National Institute on Disability, Independent Living and Rehabilitation Research (NIDILRR) prioritized the need to identify the most robust TBI COAs in the existing pool of outcome assessment tools. To advance this goal, the NINDS launched the Common Data Element (CDE) Project, which aims to improve the precision, reproducibility and cross-study comparability of data elements, including COAs. CDEs have now been established for 23 different neurologic disorders. The first iteration of outcome CDEs for TBI was published in 2010 [18], followed by updated recommendations in 2013 [19].

While the development of TBI CDEs has been an important step toward narrowing the field of potential COAs that may be selected for use in clinical trials and observational studies, a key shortcoming is that the CDEs included in the 2013 update were selected by expert consensus, not through evidentiary review. Many of the major TBI research funding agencies now mandate that grantees incorporate the recommended CDEs into their research design. Thus, investigators should have some assurance that the TBI CDEs will perform adequately, and at least as well as other COAs that have not been designated as CDEs.

In 2014, the U.S. Department of Defense established the TBI Endpoint Development (TED) initiative (#W81XWH-14-2-0176, PI: G. Manley) to identify and validate COAs and biomarkers that could serve as potential FDA Drug Development Tools (DDTs) to more accurately detect injury sequelae, identify patient subpopulations most likely to benefit from therapeutic interventions and detect changes attributable to efficacious therapies. In response to Priority #4 of the TED initiative, which called for the development of processes to assess the strength of clinical outcome measures, the authors were awarded a seed grant to design, build, and pilot-test an evidence-based assessment platform to enable the efficient, transparent, and systematic grading of TBI COAs for use in research and clinical practice. This effort culminated in the development of the *Evidence-Based Clinical Outcome Assessment Platform (“EB-COP”)*, a semi-automated tool that guides an investigator through key questions designed to ascertain if the COA fits the intended COU, and presents the supporting evidence base for this assessment. The EB-COP extends the *Consensus-based Standards for the selection of health Measurement Instruments (COSMIN)* [20], a methodology for critically appraising and selecting appropriate outcome measurement instruments, by incorporating distinct sets of quality indicators (QIs) that are specific to different purposes of use. This feature helps the user determine the contexts for which a particular COA is appropriate. In this report, we describe the EB-COP framework, discuss how the platform grades COAs for each purpose of use and review the results of EB-COP pilot testing, which was conducted on the GOSE using the Qualtrics software platform.

## Materials and methods

We convened a multidisciplinary team of experts to serve as the EB-COP Design Team. The team was composed of two TBI COA content experts who also co-led the Outcomes Core for the TED initiative (JG, MM), an evidence-based medicine methodologist (MJA), two database developers (SM, TSDG) from the American Academy of Neurology (AAN), a medical librarian (PE) from the Mayo Clinic, a regulatory affairs advisor (AR), and two TED post-doctoral fellows (AC, MB). A glossary of key terms and abbreviations is included in S1 File.

The conceptual framework adopted by the EB-COP Design Team was informed by the AAN’s *Clinical Practice Guideline Process Manual* [21], which follows recommendations proposed in two Institute of Medicine reports- “*Finding What Works in Health Care*: *Standards for Systematic Reviews*,” [22] and *“Clinical Practice Guidelines We Can Trust*,*”* [23] and guidance provided by the FDA entitled, “*Roadmap to Patient-Focused Outcome Measurement in Clinical Trials* [8].” In addition, the Design Team agreed that the EB-COP should adhere to three foundational principles:

- COAs should be assessed within distinct COUs
- Only studies meeting accepted standards for high-quality methodology [20, 22, 24, 25] should qualify for evidentiary review
- The review process should be systematic and transparent and lead to a clearly stated recommendation.

The criteria used to identify high-quality studies (i.e., those with low risk of bias) were informed by existing data quality standards, including the AAN’s Guideline Development Process Manual [21], COSMIN Checklist [26], Quality Assessment of Diagnostic Accuracy Studies (QUADAS-2) [27] and Quality in Prognostic Studies (QUIP) [28].

We selected six of the most common COA applications (i.e., purposes of use) for inclusion in the EB-COP: 1) diagnosis, 2) identification of TBI sequelae, 3) stratification of distinct TBI subgroups, 4) outcome prediction, 5) sensitivity to natural history changes, and 6) detection of treatment effects. After consulting the peer-reviewed literature [29–35] and additional on-line resources [8–10, 36, 37], 36 different QIs were identified as relevant to clinical outcome assessment. Next, we employed a two-part modified Delphi consensus process [38] to further narrow the QI pool. Prior to voting, panel members were furnished with a narrative description of each QI and summaries of previously-published recommendations concerning their use (e.g., FDA’s COA Qualification Guidelines and Clinical Trial Roadmap). The first part of the Delphi process was conducted to identify those QIs that were believed to be fundamental to all 6 purposes of use (n = 8). It was determined a-priori, that if any of the fundamental QIs were missing or inadequate, this would be considered a “fatal flaw” and the measure could not be recommended for any of the 6 purposes of use. For example, if an administration and scoring manual was not available for a particular COA, it would not be possible to ensure that the measure was being used in a standardized and reliable manner, resulting in low confidence in the measure across all applications. After the fundamental QIs were selected, a second modified Delphi poll was conducted to select QIs that were considered mandatory for each of the six purposes of use (n = 28). Depending on the number of missing purpose-specific QIs, the COA was either tentatively recommended or not currently recommended in view of the need for additional psychometric testing. A recommendation against use of the measure was levied when any of the mandatory QIs were found to be inadequate for the intended purpose. At the end of each round, participants’ votes and comments were synthesized and made available to the panel prior to voting in the next round. Consensus was defined as at least 80% agreement within three rounds of voting. A full transcript of the voting, including the instructions, voters’ responses and comments and resulting recommendations and actions, was maintained to ensure transparency in the process.

To facilitate the COA review process, the EB-COP was subsequently incorporated into a semi-automated on-line platform using Qualtrics software to facilitate data extraction and synthesis. The accompanying EB-COP Manual of Operating Procedures (MOP) (see S2 File) provides links to the on-line platform, instructs the user in how to navigate the EB-COP platform, defines every quality indicator included in the EB-COP platform, provides examples of key psychometric constructs and includes additional supporting materials to assist the user in answering the evidence questions. The software’s automation features aid the user in building the evidence question, guide the evidence review process using branch logic, display the mandatory quality indicators required for each purpose of use, produce an evidence summary table and grade the COA in relation to the COU.

## Results

**Fig 1:**
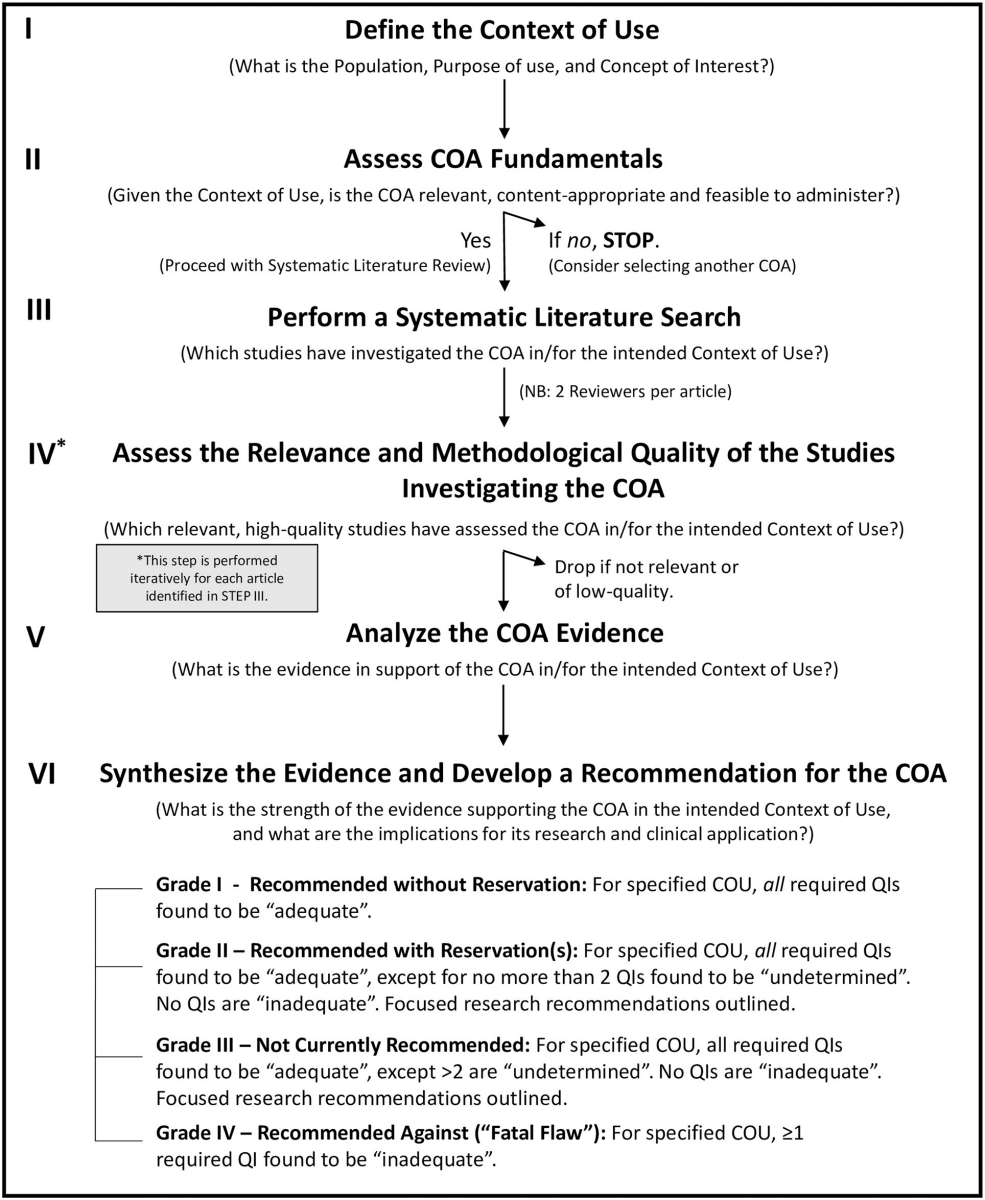
Six-step EB-COP systematic review process.

The EB-COP review process is comprised of six steps as shown in Fig 1. This procedure is intended to help ensure that the review process is systematic, transparent and considers only high-quality evidence.

### Step I: Define the context of use

The readiness of a COA for use within a specific COU is framed around an evidence question. The COU describes the: 1) population (e.g., age, injury severity, chronicity), 2) COA purpose (e.g., diagnosis, outcome prediction, detection of treatment effects), 3) concept of interest (COI)- the domain, activity or function assessed by the COA (e.g., processing speed, toileting ability, quality of life, global level of function), and 4) COA of interest (e.g., name, type, version, mode of administration). The selected COU parameters align with the TBI CDEs. After the user enters these parameters, the EB-COP software generates an evidence question. For example:

*In adult patients (i.e*., > *age 17) with acute mild TBI from blunt trauma (Population), for the purpose of predicting outcome at six months (Purpose), is short-term recall (COI) adequately measured by the Rey Auditory Verbal Learning Test* [38] *(COA)?*

### Step II: Assess fundamental quality indicators (QI)

Assessment of the COA begins by vetting the measure against a set of eight “fundamental” QIs that were selected by the Design Team through the modified Delphi process. These are: 1) the availability of documentation on how the COA was developed, 2) specification of the target population, 3) description of the intended COI, 4) information on content validity and 5) face validity, 6) clarity of the administration and scoring instructions, 7) extent of missing data in the population sampled, and 8) feasibility of application within the COU (e.g., cost, completion time, alternate forms). Table 1 shows the criteria used to assess each of the fundamental QIs. Source documents required for evaluation of the fundamental QIs include the COA’s administration and scoring manual and other materials that describe its development and use.

**Table 1:** Criteria for assessment of fundamental quality indicators (QIs).

Description of Quality Indicator	Criteria
**1) Documented Development** Availability of a manual and/or peer-reviewed article that describes the COAs conceptual framework and how it was developed. The following searchable websites may be helpful: NINDS Common Data Elements Rehabilitation Measures Database Evidence-Based Review of Moderate to Severe Acquired Brain Injury	A. Is there documentation of how the COA was developed? B. Is the documentation and instrument publicly accessible for review? C. Has the conceptual framework or theoretical background underlying the COA been described?
**2) Specification of Intended Population(s)** Description of the disease, chronicity, mechanism of injury, setting and other information that may be relevant to the target population.	A. Does the COA specify the intended population? B. If yes, is the intended population relevant to TBI, or is there evidence that the COA subsequently has been studied in a population that is relevant to TBI?
**3) Specification of Intended Concept(s) of Interest (COI(s))** Description of the construct the COA is intended to measure and its relevance to the target population.	A. Does the COA specify the concept of interest (COI) it is intended to measure? B. If yes, does the intended concept of interest match the COI specified in the evidence question in STEP I?
**4) Specification of Intended Purpose of Use** Description of the purpose for which the COA has been developed, including diagnosis, detection of TBI sequelae, subgroup stratification, outcome prediction, detection of natural history changes, detection of treatment effects.	A. Does the COA specify its intended purpose? B. If yes, does the intended purpose match the purpose specified in the evidence question in STEP I?
**5) Content Validity** Non-statistical assessment of the degree to which the COA represents all aspects of the COI it is intended to measure. Includes consideration of items, domains and corresponding response options. Typically determined by reviewing patient/caregiver focus groups and/or expert panels that informed item composition.	A. Has the content validity of the COA been tested? That is, have the items or questions in the COA been determined to have adequate coverage across all relevant facets of the COI being measured for the intended study population and purpose of use? B. If yes, does the COA exhibit adequate content validity for the COU (i.e., COI, Population, and Purpose) specified in the evidence question in STEP I of the EB-COP? C. Is the range of answers to the questions appropriate to the purpose of the COA (e.g., dichotomous answers may be sufficient for diagnostic/discriminative purposes but a greater range may be more appropriate for evaluative)? D. Are the answers or categories mutually exclusive?
**6) Face Validity** Subjective assessment of the extent to which the COA, its items and responses *appear* (at ‘face value’) to be adequate and appropriate to the COA’s intended measurement concept, population and use. Formal assessment may involve asking people—typically, non-experts—to rate the suitability of the COA to its purpose, as it appears to them.	A. Does the COA appear to measure what it intends to? B. Are the questions/items in the COA clearly worded and easy to understand? C. Are the response options appropriate to questions/items being asked? D. If a global/total score is calculated, is the method of obtaining the global/total score appropriate?
**7) Feasibility** Subjective assessment of acceptability and feasibility of the administration and scoring of the COA. Includes determination of completion time, comprehensibility, legibility, availability of language translations and/or culturally-adapted versions for multi-geographic use (if applicable) and availability of alternate forms for attenuation of practice effects (if applicable).	A. Are there established standardized administration and scoring procedures and training materials? B. Are the instructions for administration and scoring and training materials clearly worded and easy to understand? C. Is the COA available in the desired language(s)? D. Is the COA under review culturally acceptable to the population of interest, or is an acceptable culturally-adapted version of the COA available for use? E. Is the administration format/mode (e.g. patient-reported vs in-person interview) and time appropriate for the pre-specified purpose of use? F. Is the administration format/mode and time appropriate for the pre-specified Population? G. If appropriate (see Guidance), does an appropriate alternate/parallel form for your COA exist (if needed for the pre-specified COU)?
**8) Missingness/Data Quality** The extent to which expected responses to items on the COA are missing. Serves as an indicator of data quality. When >20% of items are missing, data quality is considered poor and may reflect inappropriate content, poor readability and/or lack of feasibility in a particular population.	A. In the material reviewed so far, has the number or percentage of missing items/responses in the COA been described? B. If yes, was the Population in which the number or percentage of the missing items/responses was described related to the TBI population? C. If yes to QI8-B, was the number of missing items/responses in the TBI population <20%?

The EB-COP’s on-line data entry platform defines each fundamental QI and prompts the user to respond to pre-programmed questions that determine whether the criteria for each QI has been met. The EB-COP MOP provides the user with additional guidance for responding to the questions. If the criteria for all eight fundamental QIs are met, the review process proceeds to the next step. If any one of the fundamental QIs fails, the user is advised that the COA is not appropriate for further review.

### Step III: Perform a systematic literature search

After establishing that the COA is fundamentally sound, the EB-COP prompts the user to perform a literature search to identify published abstracts of relevant, high-quality studies that have investigated the COA within the intended COU.

To assist the user in searching the literature, the Design Team adapted a pre-existing COSMIN filter to facilitate capture of articles that address the TBI population. At least two databases (e.g., OVID Medline, Web of Science, EMBASE, PsycINFO, EBSCO, CINAHL and SCOPUS) should be searched to help ensure that the review is exhaustive. Book chapters, conference proceedings, dissertations and case studies are excluded from the review. A list of TBI-related search terms that builds on recommendations from the COSMIN group [39] is included in S3 File. This list can be tailored by the user to reflect the evidence question (see Step I).

The EB-COP excludes clinical trials and observational studies that used (v. investigated) the COA to assess outcomes. This rule was based on guidance from the COSMIN group, who warned that it is not possible to use the COA as an outcome measure (for example, to assess the impact of a treatment) while concurrently testing its responsiveness [40]. If the effect size is zero, it is not possible to determine whether the lack of effect was due to an ineffective treatment or an unresponsive COA. If the effect size is moderate, either the treatment effect is moderate and the COA is responsive, or the treatment effect is large or small, but the true effect is over- or underestimated because of a poorly responsive COA.

### Step IV: Assess the relevance and methodologic quality of studies investigating the psychometric properties of the COA

Step IV is composed of four parts. In Step IVa, the abstracts retrieved in Step III are screened by the investigators to determine which articles should be acquired for full-text review. This is followed by a full-text review of the surviving studies to confirm the study’s relevance and sample size (Step IVb). The strength of the study design and generalizability of the results to the target population are assessed in Step IVc. Finally, in Step IVd, the mandatory QIs associated with the COA’s selected purpose(s) of use are examined based on the data extracted from the literature in Steps IVa-IVc. Step IV is designed to ensure that the final COA recommendations rely only on methodologically sound studies that can effectively evaluate the designated QIs. To control for bias, standardized data extraction forms are used to guide users through each of the steps described above. To help ensure reproducibility of the findings, two independent reviewers are required to screen the abstracts, extract data from the full-text articles, classify the strength of the study design and reconcile any discrepancies detected. After all of the harmonized data are entered into the on-line platform, the Qualtrics software automatically determines the final COA grade, based on the number of the number of mandatory quality criteria met.

#### Step IVa: Abstract review to assess study relevance and sample size

Step IVa essentially serves as a high-level filter, eliminating articles that are unlikely to inform the evaluation of the COA due to weaknesses in the study design. The aims of Step IVa are to capture the abstracts of all published studies that may be relevant to the defined COU (as described in Step I), and ensure that extracted studies have a sufficient sample size to assess the performance of the COA for the selected purpose(s) of use. The relevance of the study is determined by reviewing the information provided in the abstract about the: 1) population studied (e.g., do the subjects have the specified condition of interest?), 2) age group (e.g., is the target population adult or pediatric?) and 3) measurement properties studied (e.g., are the indicators studied included in the EB-COP QI list?). The user also determines if the study enrolled the minimum number of subjects required to achieve the stated aims. If the abstract indicates that the study does not meet these criteria, it is excluded, but if it lacks information or is ambiguous, the study is retained for full-text review. Upon completion of this step, the EB-COP provides a summary of the user’s responses. Step IVa essentially serves as a high-level filter, eliminating articles that are unlikely to inform the evaluation of the COA due to low relevance to the COU or insufficient sample size.

#### Step IVb: Full-text review to confirm study relevance and sample size

After both reviewers independently complete the abstract review and reconcile any disagreements about inclusion or exclusion, the full-text articles are retrieved by the user to confirm that the abstract describes an original research study and meets the requirements for relevance and sample size. Upon completion of this step, the EB-COP provides a summary of the user’s responses and indicates whether the requirements for proceeding to Step IVc have been met.

#### Step IVc: Confirmation of generalizability of results and overall methodological quality

Step IVc is comprised of a series of questions designed to confirm that: 1) the design of the study under review provides adequate protection against risk of bias, and 2) the results can be generalized to the target population. These criteria follow guidelines proposed by the AAN [21], COSMIN [26], QUADAS-2 [27] and QUIP [28]. The criteria are examined through a series of queries about the recruitment method used (e.g., consecutive series or convenience sample), range of the target population sampled (i.e., narrow or broad spectrum), whether the COA was administered and scored in accord with standardized procedures and the percentage of missing data (e.g., </> 20%). If the study meets the requirements in this area, the user is furnished with a summary of the responses and the review advances to the next step.

#### Step IVd: Assessment of the adequacy of the QIs mandated for each purpose of use

**Fig 2:**
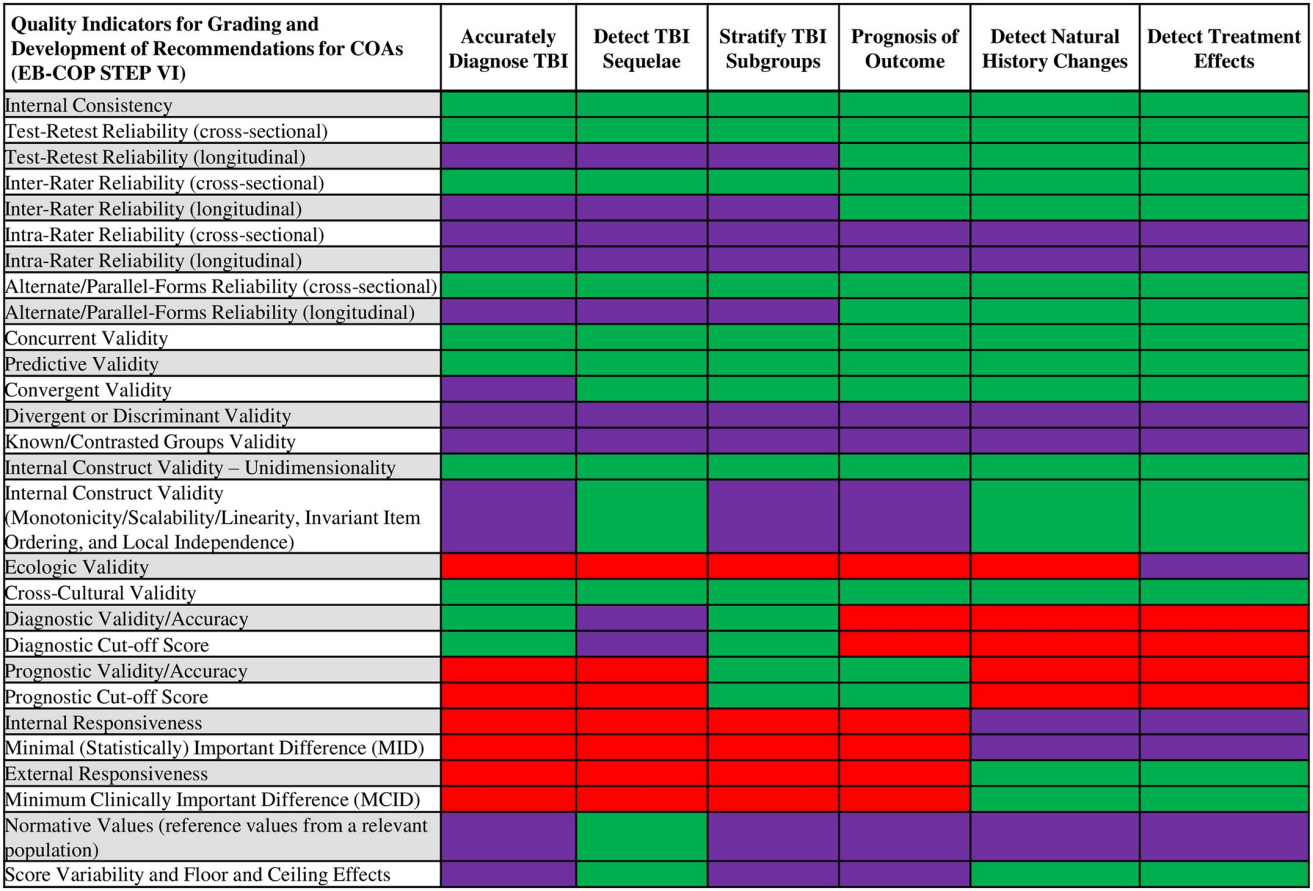
COA quality indicators organized by purpose of use and level of obligation. QIs were selected for each of the six COA applications. Mandatory QIs are shaded green, non-mandatory QIs (i.e., should be assessed if investigated) are shaded purple and QIs that are not relevant to a specific purpose are shaded red.

In step IVd, the user assesses the quality of the study investigating the QIs linked to the selected purpose of use. To ensure that only high-quality COA evidence is included in the data analysis and synthesis, only studies that meet all of the mandatory QI criteria are considered in the grading process and in the final recommendation. Fig 2 shows the QIs that have been assigned to each purpose of use. The full list of QIs included in Steps IVa through IVd is shown in the EB-COP MOP (S2 File).

### Step V: Compile the COA evidence

After all the queries about the selected QIs have been completed and entered into the EB-COP on-line tool, the data are automatically exported into an Excel-based Evidence Summary table, managed by the EB-COP Administrator based at the AAN. The Evidence Summary is then emailed back to the user by the EB-COP Administrator in preparation for the evidence analysis which is performed in Step V. Studies that survive the EB-COP review process produce evidence that is associated with no more than moderate risk of bias as determined by the AAN evidence classification scheme [21].

### Step VI: Synthesize the evidence and develop a recommendation for the COA

In Step VI, the user reviews the Evidence Summary table, which includes all the data extracted from the high-quality studies reviewed in Step IV. Acceptability cut-offs for all the QIs (Table 2) are based on pre-specified, commonly accepted cut-off values [34, 35, 37, 41]. The Evidence Summary table indicates whether each mandatory purpose-specific QI is ‘adequate’ (i.e., meets threshold), ‘inadequate’ (i.e., fails to meet threshold), or ‘not determined’ (i.e., not investigated in a high-quality study). Based on these parameters, one of the following four recommendations is made:

- Grade I—Recommended without Reservation: For the specified purpose of use, all mandatory QIs were found to be “adequate.”
- Grade II—Recommended with Reservation(s): For the specified purpose of use, all mandatory QIs were found to be “adequate,” except for no more than 2 mandatory QIs that were found to be “undetermined.” No mandatory QIs were found to be inadequate. Additional research is indicated.
- Grade III—Not Currently Recommended: For the specified purpose of use, all mandatory QIs were found to be “adequate,” however more than 2 mandatory QIs were found to be “undetermined.” No mandatory QIs were found to be inadequate. Additional research is indicated.
- Grade IV—Not Recommended: For the specified purpose of use, one or more mandatory QIs were found to be “inadequate.”

**Table 2:** Quality indicators for grading and development of recommendations for COAs.

Quality Indicators	QI Cut-Offs
Internal Consistency	Correlation coefficient ≥ 0.70
Test-Retest Reliability (cross-sectional)	Correlation coefficient ≥ 0.70
Test-Retest Reliability (longitudinal)	Correlation coefficient ≥ 0.70
Inter-Rater Reliability (cross-sectional)	Correlation coefficient ≥ 0.70
Inter-Rater Reliability (longitudinal)	Correlation coefficient ≥ 0.70
Intra-Rater Reliability (cross-sectional)	Correlation coefficient ≥ 0.70
Intra-Rater Reliability (longitudinal)	Correlation coefficient ≥ 0.70
Alternate/Parallel-Forms Reliability (cross-sectional)	Correlation coefficient ≥ 0.70
Alternate/Parallel-Forms Reliability (longitudinal)	Correlation coefficient ≥ 0.70
Concurrent Validity	Correlation coefficient ≥ 0.60
	ROC AUC ≥ 0.70
Predictive Validity	Correlation coefficient ≥ 0.60
	ROC AUC ≥ 0.70
Convergent Validity	Correlation coefficient ≥ 0.60
	ROC AUC ≥ 0.70
Divergent or Discriminant Validity	Correlation coefficient < 0.30
	ROC AUC < 0.70
Known/Contrasted Groups Validity	Cohen’s d ≥ 0.50 with P ≤ 0.05
Internal Construct Validity—Unidimensionality	Depends on the Model/Method
Internal Construct Validity (Monotonicity/Scalability/Linearity, Invariant Item Ordering, and Local Independence)	Depends on the Model/Method
Ecologic Validity	*Veridicality*–Correlation ≥ 0.70
	*Verisimilitude*– ≥70%* of items resemble tasks/activities performed in everyday life.
Cross-Cultural Validity	Process is appropriate.
Diagnostic Validity/Accuracy	Sensitivity >80%, Specificity >60%
	ROC AUC ≥ 0.80
	LR+ >10
	LR- <0.1
Diagnostic Cut-off Score	Described, with adequate diagnostic validity
Prognostic Validity/Accuracy	Sensitivity >80%, Specificity >60%
	ROC AUC ≥ 0.80
	LR+ >10
	LR- <0.1
Prognostic Cut-off Score	Described, with adequate prognostic validity
Internal Responsiveness	Direction and magnitude of change as stated in *a priori* hypothesis
Minimal (Statistically) Important Difference (MID)	Described
External Responsiveness	Correlation coefficient ≥ 0.70
	ROC AUC ≥ 0.70
Minimum Clinically Important Difference (MCID)	Described
Normative Values (reference values from a relevant population)	Described
Score Variability and Floor and Ceiling Effects	All scores <15% of sample

The EB-COP does not stipulate which statistical tests should be used to calculate the cut-off values, but some general considerations are provided in the EB-COP MOP. The final COA grade and recommendation is based on the number of mandatory QIs that meet the pre-specified cut-off values as shown in Table 2. If any of the mandatory QIs is found to be ‘inadequate,’ the lowest grade (i.e., “Recommended Against”) is assigned.

### EB-COP pilot-testing

We pilot-tested the EB-COP on the GOSE because it is among the most commonly used COAs in TBI research. The primary aim of the pilot-testing was to assess the utility of the EB-COP review process and the feasibility of the data entry platform. Pilot-testing was performed by two post-doctoral fellows who first participated in a primer on evidentiary methods conducted by the Design Team’s evidence-based medicine methodologist (MJA). Although we expected the results to be scientifically informative, our primary aim was to assess the EB-COP’s capacity to interrogate the GOSE within a well-defined COU. We chose detection of treatment effects as the purpose of use, given the GOSE’s widespread use in clinical trials. The results of each step are summarized below.

#### Step I: Determination of the GOSE evidence question

**Fig 3:**
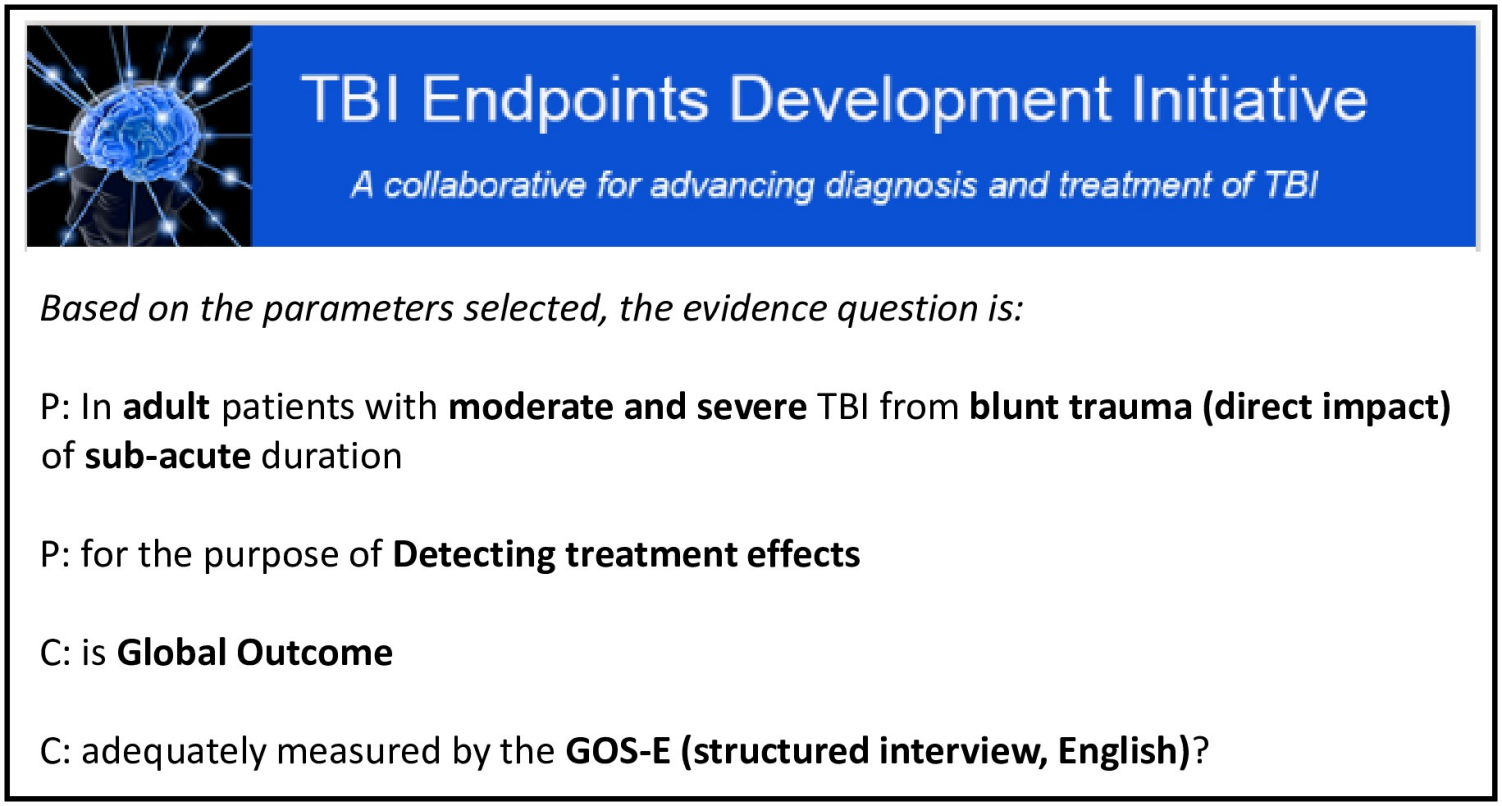
Screenshot of the evidence question generated by the EB-COP. The evidence question describes the context of use for which the COA will be tested. Contextual factors include descriptive characteristics of the sample, the chronicity of the condition, the purpose for which the COA has been selected and the concept of interest the COA purports to measure.

In framing the evidence question concerning the GOSE, we selected the following parameters from the EB-COP’s drop-down menus: population = adults with moderate to severe TBI; injury duration = subacute; cause of injury = blunt trauma; Purpose = detection of treatment effects and concept of interest = global outcome. After these parameters (shown in bold in Fig 3) were entered, the EB-COP automatically generated the evidence question as shown below in Fig 3.

#### Step II. Assessment of fundamental QIs

To assess the fundamental QIs, we identified three studies [15, 42, 43], a review article [16] and an on-line curated COA database [35] that included information about the development of the GOSE. Using these sources, we were able to assess each of the fundamental QIs. Based on the EB-COP criteria, all eight QIs were judged to be adequate, allowing the review to progress to the next step.

#### Step III: Results of the systematic literature review

The Design Team’s reference librarian (PE) performed a literature search using the terms, “traumatic brain injury,” “Glasgow Outcome Scale” AND “Glasgow Outcome Scale—Extended.” The search was refined by employing the COSMIN filter [39] that was modified to capture TBI studies. We searched OVID MEDLINE, OVID, EMBASE, PsycINFO, EBSCO, CINAHL and SCOPUS and did not restrict the search to studies written in English. Dissertations, book chapters, conference proceedings and case studies were excluded. A total of 2,849 unique abstracts were identified for review, enabling transition to Step IV.

#### Step IV: Relevance and methodologic quality of identified studies investigating the GOSE

**Fig 4:**
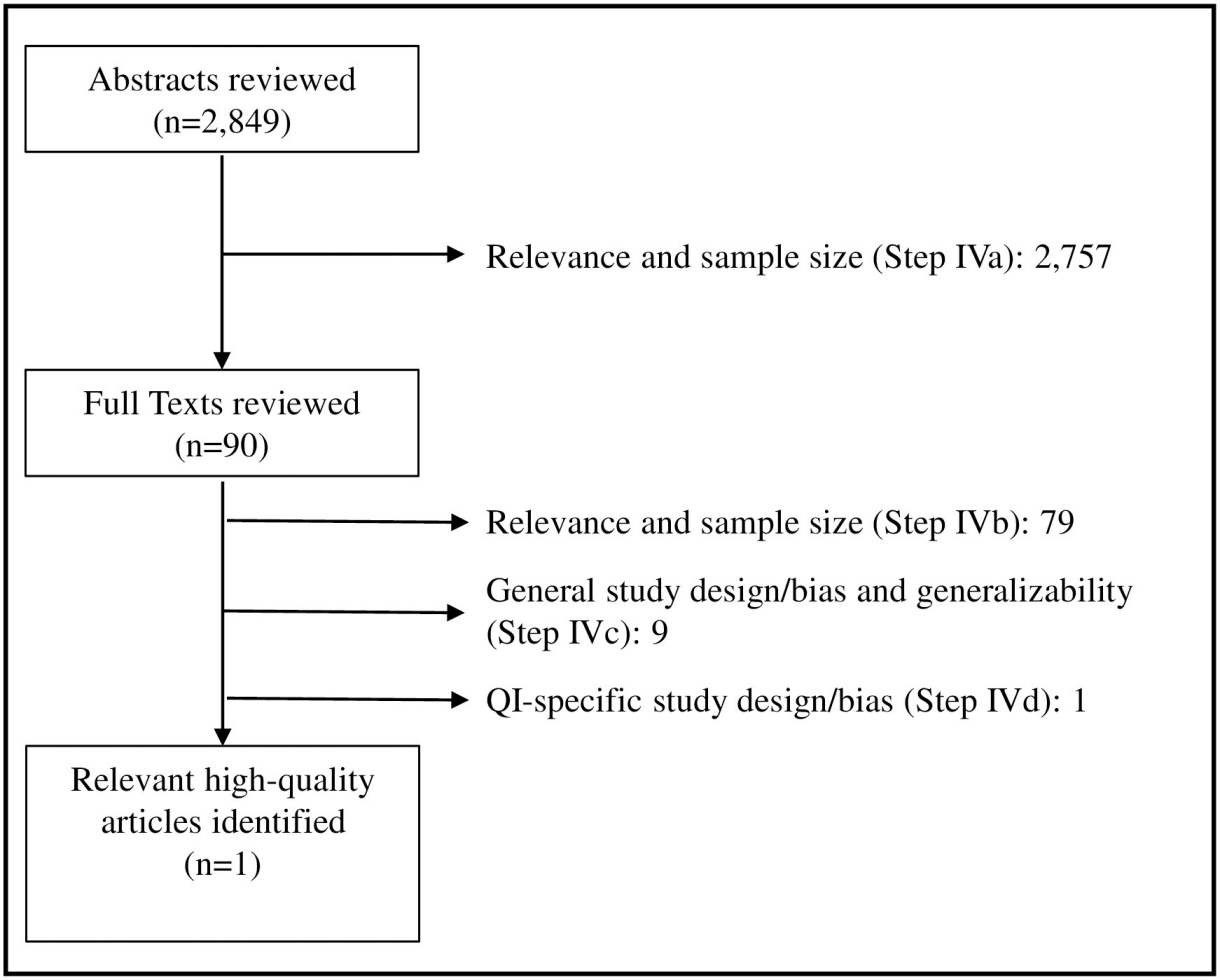
Flow diagram for the GOSE abstract and full-text review process. The EB-COP uses a two-step review process which begins with the abstract and progresses to full-text review. The arrows and numbers indicate the reasons articles were excluded from the review, and the number of articles that were excluded for each reason, respectively.

Ninety abstracts were retained for full-text review after reconciliation by the two independent reviewers (Step IVa). Approximately 6% (170) of the abstracts required reconciliation. Abstracts that could not be definitively excluded were retained for full-text review. By far, the most common reason for exclusion was that the study clearly used the COA to test an intervention, rather than investigating its psychometric properties. During the full-text review (Step IVb), the reviewers excluded 79 studies for relevance because they used (versus tested) the GOSE as an outcome measure. In Step IVc, nine more studies were excluded, either because the study design had increased risk of bias or low probability of generalization. Finally, one additional study was excluded in Step IVd because the study did not include any of the QIs required for detection of treatment effects. Fig 4 shows the flow diagram for the abstract and full-text review process. The reviewers were able to extract and evaluate the data elements included in all four parts of Step IV in a systematic and transparent manner.

#### Steps V and VI: Evidence analysis and recommendation for use of the GOSE in detecting treatment effects

The data extracted in Step IV were exported into an Excel spreadsheet by the Database Administrator and an Evidence Summary table was returned to the reviewers, indicating successful execution of Step V. Evidence analysis (Step VI) indicated that only one study met all of the EB-COP criteria required to complete the grading process. This study, published in 2003 by Pettigrew and colleagues [44], investigated two of the 16 QIs required for detection of treatment effects, test-retest and inter-rater reliability. Test-retest reliability could not be assessed due to insufficient sample size in the target population. Inter-rater reliability was assessed in a sample of 56 adults, including 25 with moderate or severe TBI. Subjects were interviewed by telephone by one rater and then face-to-face by a different rater up to one month later. The reported weighted kappa value, representing the level of agreement between the in-person and phone-based GOSE ratings, exceeded the EB-COP’s pre-specified threshold of 0.70 (K_w_ = 0.92, CI = 0.51–1.00) as shown in Table 2, however, because of the wide confidence interval, the results were considered inconclusive.

It is important to note that two highly cited papers, the original study of the GOSE structured interview by Wilson and colleagues [15], which assessed reliability, and a study by Levin and colleagues [45] that investigated the validity and sensitivity of the GOSE, were excluded by the EB-COP because their samples consisted primarily of subjects with mild TBI.

In the final analysis, while the EB-COP did not find any “inadequate” GOSE QIs, more than two QIs required for detection of treatment effects were characterized as “undetermined.” No purpose-specific GOSE data were available to assess internal consistency, test-retest reliability (i.e., cross-section, longitudinal), longitudinal inter-rater reliability, validity (i.e., construct, predictive, convergent, cross-cultural), responsiveness (i.e., minimum clinically important difference) or floor and ceiling effects. Consequently, a Grade III rating was assigned- “*Not currently recommended” for detecting treatment effects on global function in adult patients with moderate to severe TBI assessed in the subacute setting*.

*Implications for clinical trial design*. An investigator interested in using the GOSE score at 6 months post-injury as the primary endpoint in a clinical trial designed to test the benefits of a novel drug administered within 72 hours of sustaining severe traumatic brain injury would want to be confident that this measure is appropriate for detecting improvements in function that exceed those expected based on knowledge of the natural history of recovery. To achieve this, the investigator should be reasonably sure that the GOSE:

- Is comprised of items that are inter-correlated, independent of each other and behave lawfully (internal consistency, local independence, monotonicity, invariant item ordering);
- Captures a single underlying construct (unidimensionality);
- Can be reliably administered (interrater and test-retest reliability);
- Measures behaviors that are tied to functional ability, behaves like other measures of functional ability and predicts future functional status (construct, convergent and predictive validity);
- Is robust to cultural differences in the target population (cross-cultural validity)
- Detects “true” change (i.e., beyond measurement error and spontaneous recovery) that is clinically relevant across low and high performers (responsiveness, minimum clinically important difference, ceiling/floor effects).

Failure of a measure to demonstrate any one of these features can jeopardize accurate interpretation of the study findings and lead to either over-estimation or under-estimation of the effects of the intervention. The EB-COP guides the investigator through a systematic process that can be used to identify which COAs are best-suited to accurately detect treatment effects. The failure of a measure to meet the mandatory requirements does not mean that the measure should be scrapped, rather it reveals weaknesses that require further investigation before the measure can be confidently used for the desired purpose.

#### Conclusion

The pilot test results demonstrated that the EB-COP platform is a feasible method of assessing COAs within a specific COU. Guided by a Qualtrics-driven software platform underpinned by branch logic, two independent reviewers systematically and transparently navigated a comprehensive evidence-based COA review of the GOSE. Pilot-testing also provided an opportunity to re-organize the order in which some of the questions are presented and to clarify language ambiguities. These changes have been incorporated into the current version of the EB-COP.

## Discussion

The success of a clinical trial hinges, in part, on the performance of the selected COAs. Relying on existing literature on psychometric standards, well-established evidence-based methodology developed by the AAN and published FDA guidance, we developed a six-step evidence-based method (i.e., EB-COP) to evaluate COA performance within well-defined COUs, and operationalized this procedure on a semi-automated Qualtrics-driven software platform. To our knowledge, this combination of features is not available in any existing systematic review platform.

The EB-COP is distinct from the COSMIN guidelines in a number of important ways. First, it extends COSMIN by incorporating QIs that are specific to, and required for, different COA applications. This is critically important as a COA may perform well for one purpose or population, and poorly for another. As an illustration of this point, EB-COP pilot-testing suggested that the GOSE may not be suitable for detecting treatment effects in patients with moderate to severe TBI because most of the mandatory QIs for this purpose of use have not been investigated. This finding is not surprising since the GOSE was originally developed to assess global functional outcome after brain injury, not to detect treatment effects. Second, a modified Delphi consensus process involving a diverse stakeholder group was used to guide development of the EB-COP. The EB-COP’s six-tiered framework, the criteria for grading study methodology and the purpose-specific QIs were all subjected to an iterative consensus-building process. Third, the EB-COP’s grading system only considers evidence associated with moderate risk of bias or lower in accord with the AAN evidence classification scheme [21], ensuring that COA recommendations are driven only by high-quality studies. Fourth, the cloud-based Qualtrics software platform [46], used by more than 1,600 colleges and universities worldwide, will foster consistency across users and enable adaptation for use in a wide variety of clinical populations.

The EB-COP can be viewed as a decision-making tool that minimizes historical, subjective and idiosyncratic biases that influence COA selection in both clinical practice and research trials. We wish to emphasize that the EB-COP is not intended to be the only driver in determining which COAs should be selected for a specific TBI research application. Other factors, both practical and scientific, will also play a role. For example, investigators are likely to encounter situations in which a particular COA is not recommended for a specific purpose of use because of missing or inadequate QIs, but a suitable alternative is not available. Under these circumstances, some judgement will be required to determine whether the level of risk associated with the measure is acceptable, based on the number of missing or inadequate QIs, other COAs included in the assessment battery, the study aims and other considerations.

While our original focus was on TBI COAs, the EB-COP can be modified to address other populations and expanded to incorporate additional applications. From a regulatory standpoint, the EB-COP can sharpen the process used to determine the readiness of a COA for use as a clinical endpoint in drug development. This is consistent with the Agency’s efforts to encourage “community collaboration in the development of COAs for unmet measurement needs” [12] and supports its mission to develop efficient publicly-available medical device development tools for widespread use in device development programs [13]. For investigators, the EB-COP will help narrow the dense and confusing landscape of TBI COAs by identifying the most psychometrically-robust measures and the contexts in which they are most effective. This has direct relevance to the existing TBI CDEs for outcome measures. A recent critical review of CDE harmonization across three large multicenter TBI studies found substantial overlap between CDEs recommended for use during acute hospitalization and acute rehabilitation [47]. The EB-COP is well-equipped to help investigators optimize COA selection by assessing suitability for use within specific settings and phases of recovery. Finally, for clinicians, the EB-COP may improve diagnostic precision, symptom detection, prognostic accuracy and treatment monitoring through its inclusion of purpose-specific QIs.

The EB-COP has some limitations. Although selection of the QIs used in the EB-COP review process was guided by existing psychometric standards and approved by COA content experts from scientific, regulatory and clinical sectors using a formal consensus process, it is important to show that the grading scheme has criterion validity. This is typically accomplished by comparing a new measure to a “gold standard.” While no clear-cut gold standard exists, one approach might be to compare performance ratings generated for the same COA by the EB-COP and the COSMIN Risk of Bias Checklist [48]. A second issue is that while the EB-COP was designed for use across a variety of COA types (e.g. patient, clinician, observer-reported or performance-based), pilot-testing was limited to the GOSE, a clinician-reported COA. A separate unpublished study used the EB-COP to assess the appropriateness of the Rivermead Post-Concussion Questionnaire (RPQ) [49], a patient-reported COA, for three purposes of use- detecting post-concussive symptoms, stratifying PCS subtypes and monitoring resolution of PCS (see S4 File). Results demonstrated the feasibility of the EB-COP for assessment of the RPQ and were very similar to the findings for the GOSE. The review did not show that the RPQ was inadequate for the purposes specified, rather, it demonstrated that most of the QIs that are critical to these three applications have not been investigated. Additional field-testing is warranted to assess the generalizability of the EB-COP to other types of TBI COAs, including those intended for use in other health conditions. Third, the EB-COP is not designed to assess COAs that serve as screening measures. Screening is a distinct purpose of use that requires its own set of QIs, some of which differ from those currently included in the EB-COP. Future iterations of the EB-COP can be adapted to incorporate screening and other purposes of use. Fourth, budgetary constraints prevented us from conducting cognitive testing on the questions that guide the user through the EB-COP review. We intend to make the EB-COP publicly accessible so there should be ample opportunity to obtain user feedback for future updates. We envision the EB-COP as a “living” tool that can be modified and expanded as the field progresses. The EB-COP software platform can also be enhanced further by incorporating additional semi-automation technologies, including narrative search tools, to further streamline data extraction and synthesis. Lastly, the TBI literature, in general, suffers from a lack of well-designed psychometric studies on TBI outcome measures, despite the fact that they are integral to almost every type of TBI study. The utility of the EB-COP is dependent, in part, on the breadth and depth of the psychometric literature. The findings discussed above on the GOSE and RPQ illustrate this point. A better understanding of how COAs behave within different contexts is essential to improving precision in TBI research. The EB-COP can serve as a blueprint to aid in this endeavor.

## Conclusion

We developed a semi-automated evidence-based platform (i.e., “EB-COP”) to facilitate the evaluation of TBI COAs for use in research and clinical practice. This tool is unique in that it includes quality indicators that assess the performance of TBI COAs within specific *a-priori*-defined COUs. This feature is critically important as it considers the psychometric strengths and limitations of the instrument in relation to the target population and intended purpose. These characteristics may help reduce type II error in research and improve diagnostic and prognostic sensitivity in clinical settings. Although designed for use in TBI, the underpinnings of the EB-COP framework are cross-cutting and may be applicable to other health conditions.

## Supporting information

S1 File List of abbreviations. (DOCX)

S2 File EB-COP Manual of Operating Procedures (MOP). The MOP guides the EB-COP user through the review process by asking a series of questions about psychometric indicators that are important in judging the relevance and strength of the COA. Links are provided in the MOP to the on-line Qualtrics Survey Software System. The software platform streamlines the review process by efficiently navigating the EB-COP user through the review process, and ultimately produces a recommendation.
(PDF)

S3 File List of search terms to promote capture of studies on TBI outcome measures. The EB-COP search filter extends the filter recommended by COSMIN by adding terms that help identify studies focusing on TBI outcome measures.
(DOCX)

S4 File EB-COP review of the Rivermead Post-Concussion Questionnaire (RPQ). The RPQ is a widely-used patient-reported measure of symptom severity following mild TBI. In this study, we assessed the Rivermead to address the following evidence question: *In adult patients with mTBI of subacute (<6mo) duration*, *for the purpose of (1) detecting PCS*, *(2) stratifying sub-types*, *or (3) monitoring the resolution or progression of PCS*, *are PCS adequately measured by the English version of the patient-reported RPQ*? Seventy-nine full-text articles were reviewed by two independent reviewers and four qualified for EB-COP review. The two most common reasons for exclusion were study design (e.g., conference abstracts, review articles, books) and lack of relevance to the evidence question. Results indicated that there is paucity of relevant, high-quality evidence addressing the performance of the RPQ within the three COUs assessed. As such, the RPQ was not recommended for these applications.
(PDF)
