# Supplementary material for: An evidence-based methodology for systematic evaluation of clinical outcome assessment measures for traumatic brain injury
Source: PLoS One. 2020 Dec 14;15(12):e0242811. doi: 10.1371/journal.pone.0242811 (PMC7735614; doi:10.1371/journal.pone.0242811)
Supplement: S1 File — (DOCX) [file pone.0242811.s001.docx]

**S1 File. Glossary of Abbreviations**

AAN American Academy of Neurology

CDE Common data element

COA Clinical outcome assessment

COI Concept of interest

COSMIN Consensus-based Standards for the selection of health Measurement Instruments

COU Context of use

DDT Drug development tool

DoD Department of Defense

EB-COP Evidence-Based Clinical Outcome Assessment Platform

FDA Food and Drug Administration

GOS-E Glasgow Outcome Scale– Extended

IOM Institute of Medicine

MOP Manual of Operating Procedures

NINDS National Institute of Neurological Disorders and Stroke

NIDILRR National Institute on Disability, Independent Living and Rehabilitation Research

QI Quality indicators

QUADAS-2 Quality Assessment of Diagnostic Accuracy Studies

QUIP Quality in Prognostic Studies

ROC AUC Receiver operating characteristic area under the curve

TBI Traumatic brain injury

TED TBI Endpoint Development
