# Supplementary material for: An evidence-based methodology for systematic evaluation of clinical outcome assessment measures for traumatic brain injury
Source: PLoS One. 2020 Dec 14;15(12):e0242811. doi: 10.1371/journal.pone.0242811 (PMC7735614; doi:10.1371/journal.pone.0242811)
Supplement: S2 File — The MOP guides the EB-COP user through the review process by asking a series of questions about psychometric indicators that are important in judging the relevance and strength of the COA. Links are provided in the MOP to the on-line Qualtrics Survey Software System. The software platform streamlines the review process by efficiently navigating the EB-COP user through the review process, and ultimately produces a recommendation. (PDF) [file pone.0242811.s002.pdf]

Manual of Operating Procedures

An Evidence-Based Clinical Outcome Assessment Platform for Validation of TBI Assessment Measures (EB-COP)

(Version 2)

EB-COP Development Team
Joseph T. Giacino (PI), Michael McCrea, Melissa J. Armstrong,
Ann Robbins, Thomas Getchius, Shannon Merillat, Patricia Erwin,
Michael Bergin, Andrea Christoforou, Sabrina Taylor,
Geoff Manley (Administrative PI)

Funded by the Department of Defense (Award # W81XWH-14-2-0176)

Table of Contents

Definitions **3**

Introduction **5**

Figure 1 6

***Step I*: Specify Population, Purpose of Use, Concept of Interest, and COA (P-P-C-C)** **7**

EB-COP Validation Framework 9

***Step II*: Assess COA Fundamentals** **12**

Fundamental Quality Indicators 12

***Step III*: Perform Systematic Literature Search** **23**

***Step IV*: Assess the Relevance and Methodological Quality of the Studies Investigating the COA** **24**

Step IV – A: Abstract Review: Assess All Articles' Relevance to the P-P-C-C and Sample Size 25
Step IV – B: Full-text Review: Confirm Relevance to P-P-C-C and Sample Size 26
Step IV – C: Full-text Review: Confirm Article's Generalizability to P-P-C-C and Overall Methodological Quality 28
Step IV – D: Full-text Review: Assess the Methodological Quality of the Article at the Level of the QI 28

***Step V*: Analyze the COA Evidence** **81**

***Step VI*: Synthesize the Evidence and Develop a Recommendation for the COA** **82**

Acknowledgments **83**

References **83**

Definitions

KEY TERMS AND ABBREVIATIONS

**Clinical outcome assessment (COA)**	Any tool that measures a patient's symptoms, overall mental state, or the effects of a disease or condition on how the patient functions. It may be influenced by human choices, judgment, or motivation. Furthermore, a COA's measurement ability may be delineated by the context in which it is used (see COU below) and depends on the implementation, interpretation, and reporting from a patient, a clinician, or an observer. The four types of COAs are patient-reported outcome (PRO) measures, clinician-reported outcome (ClinRO) measures, observer-reported outcome (ObsRO) measures, and performance outcome (PerfO) measures. The FDA defines each of these types as follows:<br><br>**ClinRO** – A COA that is based on a report that comes from a trained health-care professional after observation of a patient's health condition and involves a clinical judgment or interpretation of the observable signs, behaviors, or other physical manifestations thought to be related to a disease or condition. It cannot directly measure the symptoms that are known only to the patient (e.g. pain intensity).<br><br>**ObsRO** – A COA that is based on an observation by someone other than the patient or a health professional, including a parent, spouse, non-clinical caregiver, or other individual in a position to regularly observe the patient in daily life and report on a specific aspect of the patient's health. It cannot include medical judgment or interpretation.<br><br>**PRO** – A COA that is based on a report that comes from the patient about the status of their health condition without amendment or interpretation of the patient's report by a clinician or anyone else. It can be measured by self-report or by interview, and is the only means by which symptoms or other unobservable concepts known only to the patient can be measured. It is also used to assess the patient's perspective of their level of functioning or degree of participation.<br><br>**PerfO** – A COA that is based on the task(s) performed by a patient, according to standardized instructions that are delivered by a health care professional. It requires patient understanding, cooperation and motivation and may include measures of gait speed, memory recall or general cognitive abilities.
**Concept of Interest (COI)**	The trait, skill, knowledge, sign, symptom, perception, limitation or any "thing" that represents a meaningful aspect to the patient experience that is intended to be measured by the COA.
**Context of Use (COU)**	A comprehensive statement that fully and clearly delineates the way in which the COA will be employed in the study or clinical trial, including its drug development-related purpose of use. It defines the boundaries or circumstances within which the COA is deemed qualified to be used meaningfully, accurately and effectively.
**Evidence-Based Clinical Outcome Assessment Platform (EB-COP)**	The step-by-step evidence-based approach/process currently being developed to evaluate Clinical Outcome Assessments (COAs) for Traumatic Brain Injury (TBI). The process will result in a final grade and associated recommendation for the COA that will be based on the evaluation of the pre-selected quality indicators (QIs). As

	part of the grading process, each QI will be vetted against a pre-specified cut-off and rated as *"adequate"* if it meets the cut-off, *"inadequate"* if it fails to meet the cut-off or *"undetermined"* if it has not been investigated in a study of sufficient methodological quality.
**Quality Indicator (QI)**	The measurement property (e.g. inter-rater reliability and criterion validity) that evaluates an aspect of the ability and utility of the COA to measure the trait, skill, symptom or other concept of interest (COI).
**Purpose of Use (PoU)**	**PoU1) Accurately Diagnose Traumatic Brain Injury (TBI)**<br>- To discriminate between those with and without TBI.<br><br>**PoU 2) Detect TBI Sequelae**<br>- To detect, for e.g., physical, cognitive or behavioral deficits due to the TBI<br>- This may be determined in comparison to pre-established normative population values or in comparison to a non-TBI group.<br><br>**PoU 3) Stratify TBI Subgroups**<br>- To discriminate individuals with TBI on a specific dimension, e.g., severity, risk of late decline.<br>- NB: Stratification based on trajectory of (natural) change or responsiveness to treatment is also plausible. However, the Modified Delphi for the selection of quality indicators has not accommodated for this longitudinal operationalization of PoU3. Guidance will be developed to accommodate this operationalization in the pilot/beta version of the EB-COP. For example, the user may be advised to first evaluate the COAs ability to detect change (e.g., PoU5) as well as its ability to stratify (i.e., PoU3), thus merging the QIs selected for the two PoUs (e.g., PoU3+PoU5).<br><br>**PoU 4) Predict TBI Outcome**<br>- To project a future outcome, e.g., mortality, degree of disability<br>- NB: Prognosis based on rate of change is also plausible. However, the Modified Delphi for the selection of quality indicators has not accommodated for this operationalization of PoU4. Guidance will be developed to accommodate this operationalization in the pilot/beta version of the EB-COP. For example, the user may be advised to first evaluate the COAs ability to detect change (i.e., PoU5) as well as its ability to predict (i.e., PoU4), thus merging the QIs selected for the two PoUs (e.g., PoU4+PoU5).<br><br>**PoU 5) Identify Natural History Changes**<br>- To detect changes in the COA over time (e.g., recovery of sequelae; global outcome) not associated with exposure to an intervention.<br><br>**PoU 6) Detect Treatment Effects**<br>- To detect the effects of exposure to/withdrawal from a specific intervention.

Introduction

Among the more than 1,000 clinical outcome assessment measures (COAs) currently used for traumatic brain injury (TBI), few have been systematically evaluated to determine their performance within specific "contexts of use (COU)." As described by the U.S. Food and Drug Administration, the COU specifies the population of interest and the purpose for which the COA will be employed. COAs are commonly used for screening, diagnostic categorization, outcome prediction and establishing treatment effectiveness. Despite the pivotal role that outcome assessment plays in research, COA selection typically relies on expert consensus. There is currently no methodology designed to determine the appropriateness of a particular COA within a specific COU. To address this gap, we developed and pilot-tested the *Evidence-Based Clinical Outcome Assessment Platform (EB-COP)* to efficiently and transparently evaluate the suitability of TBI COAs for specific purposes of use. Development of the EB-COP was informed by the FDA's Roadmap to Patient-Focused Outcome Measurement in Clinical Trials,[1] the American Academy of Neurology's (AAN's) well-established Clinical Practice Guideline Process Manual,[2] the COnsensus-based Standards for the selection of health Measurement INstruments (COSMIN)[3] and other literature describing standards for measurement development.

The framework of the EB-COP is built around the six distinct "Purposes of Use" (PoUs) shown below:

1. Accurate diagnosis of TBI
2. Detection of TBI sequelae
3. Stratification of TBI subpopulations
4. Prediction of TBI outcome
5. Identification of natural history changes
6. Detection of treatment effects

The EB-COP and evaluates the strength of COAs using distinct sets of quality indicators (QIs) that are specific to each purposes of use. This feature helps the user determine the contexts within which the COA should and should not be used.

This Manual of Operating Procedures (MOP) guides the EB-COP user through the COA review process. which relies on the on-line Qualtrics Survey Software System. The Qualtrics platform streamlines the process by efficiently navigating EB-COP user through the review process. Using logic, the software is able to populate the relevant criteria for the user to evaluate and generate recommendations based on users' responses.

See page 1 for a list of the key terms, their abbreviations and definitions. In this MOP is a description of each of the six EB-COP Steps. The core components of the six-step COA evaluation process is depicted in Figure 1. The links to the EB-COP platform are below (Steps III and V completed outside Qualtrics):

Step I: https://aan.co1.qualtrics.com/jfe6/form/SV_6VDB0Jpx4Oepr2R

Step II: https://aan.co1.qualtrics.com/jfe/form/SV_eb4bYQjmlUet1T7

Step IV-A: https://aan.co1.qualtrics.com/jfe/form/SV_29T0wYcIDmIfLM1

Step IV-B–D: https://aan.co1.qualtrics.com/jfe/form/SV_5chr9XxF1GHl3JX

Step VI: https://aan.co1.qualtrics.com/jfe/form/SV_8BOFAW5atR9jNqd

I **Define the Context of Use**

(What is the Population, Purpose of use, and Concept of Interest?)

II **Assess COA Fundamentals**

(Given the Context of Use, is the COA relevant, content-appropriate and feasible to administer?)

Yes
(Proceed with Systematic Literature Review)

If *no*, **STOP**.
(Consider selecting another COA)

III **Perform a Systematic Literature Search**

(Which studies have investigated the COA in/for the intended Context of Use?)

(NB: 2 Reviewers per article)

IV* **Assess the Relevance and Methodological Quality of the Studies Investigating the COA**

(Which relevant, high-quality studies have assessed the COA in/for the intended Context of Use?)

*This step is performed iteratively for each article identified in STEP III.

Drop if not relevant or of low-quality.

V **Analyze the COA Evidence**

(What is the evidence in support of the COA in/for the intended Context of Use?)

VI **Synthesize the Evidence and Develop a Recommendation for the COA**

(What is the strength of the evidence supporting the COA in the intended Context of Use, and what are the implications for its research and clinical application?)

- **Grade I - Recommended without Reservation:** For specified COU, *all* required QIs found to be "adequate".
- **Grade II – Recommended with Reservation(s):** For specified COU, *all* required QIs found to be "adequate", except for no more than 2 QIs found to be "undetermined". No QIs are "inadequate". Focused research recommendations outlined.
- **Grade III – Not Currently Recommended:** For specified COU, all required QIs found to be "adequate", except >2 are "undetermined". No QIs are "inadequate". Focused research recommendations outlined.
- **Grade IV – Recommended Against ("Fatal Flaw"):** For specified COU, ≥1 required QI found to be "inadequate".

Figure 1. EB-COP Flowchart illustrating the EB-COP 6-Step Process and COA Grading Approach

***Step I*: Specify Population, Purpose of Use, Concept of Interest, and COA (P-P-C-C)**
(What is the intended Context of Use, and which COA has been selected for review (i.e., P-P-C-C)?)

Step I serves to frame the evidence question that will direct the literature search and review toward the determination of whether the COA under investigation is 'fit for purpose'. This step is akin to specifying the PICO framework in other systematic review processes. In order to frame the evidence question, the WHO (POPULATION), WHY (PoU), WHAT (COI), and HOW (COA/type/mode) must be specified. In particular, specification of the Population and PoU results in the specification of the COU, which the FDA defines as the 'boundaries' within which the COA is qualified for use. Specifying the COU also defines the boundaries of this evidentiary review.

The Validation Framework (presented below) lists the values (i.e., parameters and options offered in the drop-down menus) currently supported by the EB-COP, but the user is free to make specifications beyond those supported (e.g., via free-text boxes in the Qualtrics web-based version).

1. Population
   Specify relevant parameters for age (pediatric, adult, older adult), sex (male, female), TBI severity (e.g. mild, moderate, severe), cause of TBI (e.g., fall, blunt trauma, MVA, sports, blast), chronicity (e.g., acute, subacute, chronic), setting (e.g., ICU, hospital, rehab, outpatient) and other domains relevant to the natural history, onset, duration or pathophysiology of TBI.

2. Purpose of Use
   Specify the Purpose of Use:
   1. Accurately diagnose TBI
   2. Detect TBI sequelae
   3. Stratify TBI subgroups
   4. Predict outcome
   5. Identify natural history changes
   6. Detect treatment effects

3. Concept of Interest
   Specify the construct, trait, or aspect of the patient experience – e.g. how a patient survives, feels, functions – to be measured by the COA. These may include global outcome, level of consciousness, quality of life or aspects of neuropsychological function. Select the domain, and sub-domain if applicable, under which the COI falls. Domains and sub-domains mirror those from the National Institute of Neurological Disorders and Stroke (NINDS) TBI common data elements (CDE).

4. COA
   a. Specify the name of the COA under investigation: <<text entry only>>
   b. Specify the type of COA that will be used:
      1. Patient-reported outcome (PRO)
      2. Observer-reported outcome (ObsRO)
      3. Clinician-reported outcome (ClinRO)
      4. Performance outcome (PerfO)
   c. Specify the Administration Mode (e.g. in person, by phone) of the COA under investigation.
   d. Specify the Language(s) and/or Cultural Adaptation(s) required.

You will be asked to provide additional specifications with respect to the P-P-C-C that may be relevant to the parameters of the Review (e.g., Population: +/- presence of a biomarker, or COI: disability due to TBI vs overall trauma).

Based on the P-P-C-C parameters selected above, the evidence question will be presented in the web-based version of Qualtrics. You will be asked to provide an email address. You will receive a copy of your evidence questions, a record of your other specifications, and a link to the next step, Step II, at the email address that you provide.

You may elect to proceed to Step II or end the session and complete Step II at a later time.

EB-COP Validation Framework (for P-P-C-C Evidence Question in Step I)

FRAMEWORK CONCEPT	DOMAIN	SUB-DOMAIN (items in Drop-down Menu)
**Population**	Age<br>*Specify the relevant age range e.g. if adults, “>18,” “18–65,” etc.:*	Pediatric
		Adult
		Older Adult
	Gender	Female
		Male
	TBI Severity<br>*Specify criteria:*<br>*For example, measures of severity based on: 1) Glasgow Coma Scale {range} (e.g., GCS≤8 for severe TBI or GCS≥13 and +CT for complicated mild TBI); 2) Duration of post traumatic amnesia {range}; and/or 3) imaging findings	Mild
		Complicated Mild
		Moderate
		Severe
		Other (e.g., DOC or mixture)
	Type of TBI	Acceleration/deceleration
		Blast exposure (military)
		Blunt trauma (direct impact)
		Closed (non-penetrating)
		Crush
		Fall
		Motor vehicle accident (MVA)
		Penetrating (open)
		Sports-related
		Other (e.g., Motorcycle crash [MCC]; bicycle on car crash)
	Chronicity<br>*Specify corresponding time post-injury:*	Acute
		Sub-acute
		Chronic
		Other (e.g., mixture)
	Setting	Emergency Department
		Intensive Care
		Inpatient Rehabilitation (e.g., IRF, SNF, LTAC)
		Outpatient Clinic
		Sports
		Home Care

		Community Residential
		Military
		Other (e.g., SNF only)
**Purpose of Use (PoU)**		Accurately diagnose TBI
		Detect TBI sequelae
		Stratify TBI subgroups
		Predict TBI Outcome
		Identify natural history changes
		Detect treatment effects
**FRAMEWORK CONCEPT**	**DOMAIN (from NINDS TBI CDEs)**	**SUB-DOMAIN (from McCrea TRACK structure)**
**Concept of Interest (COI)**	Global Outcome	Global Outcome
		Other (specify)
	Recovery of Consciousness/Memory	Recovery of Consciousness/Memory
		Other (specify)
	Post-concussive/TBI-Related Symptoms	Post-concussive/TBI-Related Symptoms
		Other (specify)
	Neuropsychological Function	Attention
		Executive function
		General intelligence
		Language and communication
		Memory
		Visuoperceptual
		Other (specify)
	Psychiatric and Psychological Status	Anxiety
		Depression
		Post Traumatic Stress Disorder
		Personality
		Psychosis
		Substance abuse
		Other (specify)
	Physical Function	Balance
		Mobility
		Motor
		Sensory

		Other (specify)
	Social Role Participation and Social Competence	Social Role Participation and Social Competence
		Other (specify)
	Health-Related Quality of Life (Generic and Disease-Specific)	Health-Related Quality of Life
		Other (specify)
	Effort/Symptom Validity	Effort/Symptom Validity
		Other (specify)
**FRAMEWORK CONCEPT**	**DOMAIN**	**SUB-DOMAIN (items in Drop-down Menu)**
**Clinical Outcome Assessment (COA)**	Name	<<allow for free text entry of COA they choose to review>>
	Type	Patient-Reported Outcome (PRO)
		Observer-Reported Outcome (ObsRO)
		Clinician-Reported Outcome (ClinRO)
		Performance Outcome (PerfO)
	Administration Mode	In-person/face-to-face
		Via telehealth
		Video recording
		Static image(s)
		Over the phone
		By post
		Other (Specify)
	Language(s) and/or Cultural Adaptation(s)<br>*e.g., English for Australians or Portuguese for Brazilians:*	<<allow for free text entry of language(s) and/or cultural adaptation required>>
**Additional Specifications (optional)**	Additional specifications with respect to the P-P-C-C that may be relevant to the parameters of the Review<br>e.g., Population: +/- presence of a biomarker, or COI: disability due to TBI vs overall trauma	<<allow for free text entry of additional specifications>>

***Step II*: Assess COA Fundamentals**
(Given the Context of Use, is the COA relevant, content-appropriate and feasible to administer?)

Step II serves to determine whether the COA under investigation is appropriate in content and feasibility for measuring the desired COI in the specified population given the specified PoU. In Step II, you will assess the 8 fundamental Quality Indicators (QIs) listed at the bottom of this page. These QIs have been selected via a modified Delphi approach to be the most essential to a COA for your pre-specified P (population), P (PoU), and C (COI) (i.e., the evidence question formulated in Step I).

These 8 QIs have been identified as the most essential based on models of outcome measure development, evaluation and regulatory qualification (e.g., FDA) to ensure that the COA is appropriate in content, purpose, and appropriateness. You will be investigating the development of the COA, its content and face validity, feasibility and applicability to your intended context of use. To do this, you will review the items making up the COA, its user manual, early papers of its development, and administration. In this step, if any of the 8 fundamental QIs is not met based on pre-defined criteria, the user will be advised to discontinue the review and select a different COA, unless otherwise indicated. Some questions aim to gather additional information regarding the COA in preparation for subsequent Steps in the EB-COP Review. Descriptions/definitions are provided for each QI, which is operationalized via the associated “judgement criteria”.

At the start of Step II, you will be asked to provide your email. You will also be asked to re-enter or paste the COA of interest and P-P-C-C question developed in Step I. (You may refer to the email that you received after completing Step I.) These will be displayed at the top and bottom of the page, respectively, as you advance through answering questions surrounding each of the 8 QI’s judgment criteria, which are defined starting on the next page. You will also be presented with a concluding prompt that offers the opportunity to note any other evidence that the COA, as it was framed in terms of the P-P-C-C parameters specified in Step I, fails to meet the fundamental QIs. At the end of Step II, a summary of your responses will be displayed. You will receive a copy of this summary and further information to the email address provided at the beginning of Step II.

Fundamental Quality Indicators
QI1: Documented Development
QI2: Specification of Intended Population(s)
QI3: Specification of Intended Concept of Interest (COI)
QI4: Specification of Intended Purpose(s) of Use (POU(s))
QI5: Content Validity
QI6: Face Validity
QI7: Feasibility
QI8: Missingness in the context of the COA

(If the answer to any of the following questions (unless otherwise indicated) is “NO,” STOP. Explain why you responded “NO” in the comment box and then submit the form. The COA is not appropriate for further review. Otherwise, proceed with systematic literature review.)

Fundamental Quality Indicator	Judgment Criteria
**1) Documented Development**<br><br>The existence of publicly accessible documentation - a manual and/or peer-reviewed article - describing the development of the COA. The documentation should include a description of the conceptual framework underlying the COA and associated content and provide the information necessary for evaluating the remaining QIs in Step II (COA Fundamentals) of the EB-COP. A general search for the article/manual describing its development should be sufficient for most COAs. Sites such as NINDS Common Data Elements, Rehabilitation Measures Database, COMBI and ERABI may be good starting points for locating such documentation as well as basic information about the COA. Review the available evidence obtained regarding document development before answering the following questions.	**A. Is there documentation of how the COA was developed? <<Yes/No>>**<br>***If yes, please provide reference or link to this documentation:***<br><br>**B. Is the documentation and instrument publicly accessible for review? <<Yes/No>>**<br>*Guidance: Answer NO if there are any non-negotiable restrictions to its access. These restrictions should* ***not*** *include factors such as costs, licensing or training requirements or language translations, which generally can be reasonably overcome (e.g., costs associated with accessing or translating the documentation can be factored into grant applications).*<br>***If yes, please indicate if there are any costs and/or other requirements associated with accessing the COA content and/or its documentation for review:***<br>***If no, please specify why the COA content and/or documentation cannot be accessed:***<br><br>**C. Has the conceptual framework or theoretical background underlying the COA been described? <<Yes/No >>**

Fundamental Quality Indicator	Judgment Criteria
**2) Specification of Intended Population(s)**<br><br>Clear specification and justification of the Population(s) for which this COA was initially developed or subsequently adapted, including an indication of whether this COA is disease-specific or generic. The condition (e.g., TBI), chronicity of the condition, mechanism of injury or reason for onset (e.g. sports or blast), setting or other information that may be relevant to the Population in which the COA is intended for use should be specified, as appropriate.	**A. Does the COA specify the intended population? <<Yes/No>>**<br>*Guidance: The purpose of this first prompt is to determine if the COA developers specified the intended Population(s), which may or may not have been relevant to TBI, as this is a 'hallmark' of good study design for instrument development.*<br><br>***If yes, indicate the intended Population(s):***<br><br>**B. Is the intended population relevant to TBI, or is there evidence that the COA subsequently has been studied in a population that is relevant to TBI? <<Yes/No>>**<br>*Guidance: The purpose of this follow-up prompt is to determine if the COA has been developed specifically for or, at the very least, previously studied in the Population of interest, as specified in Step I. To answer this prompt, it may be necessary to perform a brief search to determine whether the COA under review has been studied in the TBI Population. Rehab Measures DB and/ TBI-specific outcome measures databases (e.g., COMBI[3] [http://www.tbims.org/combi/], NINDS CDE [https://commondataelements.ninds.nih.gov/Traumatic%20Brain%20Injury) may serve as a good starting point.*<br><br>***If yes, please specify whether the COA has been developed (i) specifically for TBI, (ii) for another population, but subsequently studied in TBI, or (iii) as a generic instrument that has also been studied in TBI:***

Fundamental Quality Indicator	Judgment Criteria
**3) Specification of Intended Concept of Interest (COI)**<br><br>Clear specification and justification of the COI(s) the COA is intended to measure. Evidence of the relevance of the COI(s) to the Population in which it is being measured should also be provided. For example, in measuring degree of global disability in patients with severe TBI with the GOS-E, it should be evident that it is intended to measure disability that results from the TBI and not from other factors, such as coexisting orthopedic injuries.	**A. Does the COA specify the concept of interest (COI) it is intended to measure? <<Yes/No>>**<br>*Guidance: The purpose of this first prompt is to determine if the COA developers specified the intended Concept of Interest (COI), as this is a 'hallmark' of good study design for instrument development.*<br><br>***If yes, indicate the intended COI:***<br><br>**B. Does the intended concept of interest match the COI specified in the evidence question in STEP I? <<Yes/No>>**<br>*Guidance:* The purpose of this follow-up prompt is to determine if the developers' intended COI corresponds with the COI specified when building the evidence question in Step I.<br><br>***If no, describe difference or discrepancy between the two (optional):***

Fundamental Quality Indicator	Judgment Criteria
**4) Specification of Intended Purpose(s) of Use (PoU(s))**<br><br>Clear specification and justification of the purpose for which the COA has been developed, which may include one or more of the six targeted PoUs: (1) Accurately Diagnose TBI; (2) Detect TBI Sequelae; (3) Stratify TBI Populations; (4) Predict TBI Outcome; (5) Detect Natural History Changes; and (6) Detect Treatment Effects. For example, in the COMBI review of the Coma Recovery Scale - Revised, the intended PoUs are specified as follows: "The purpose of the scale is to assist with differential diagnosis, prognostic assessment and treatment planning in patients with disorders of consciousness." [http://www.tbims.org/combi/crs/].	**A. Does the COA specify its intended purpose(s) of use (PoUs)? <<Yes/No>>**<br>Guidance*:* The purpose of this first prompt is to determine if the COA developers specified the intended Purpose of Use (PoU), as this is a 'hallmark' of good study design for instrument development.<br><br>***If yes, indicate the intended POU:***<br><br>**B. Does the intended purpose of use match the PoU specified in the evidence question in Step I? <<Yes/No>>**<br>Guidance***:*** *The purpose of this follow-up prompt is to determine if the developers' intended PoU corresponds with the PoU specified when building the evidence question in Step I.*<br><br>***If no, describe difference or discrepancy between the two (optional):***

Fundamental Quality Indicator	Judgment Criteria
**5) Content Validity**<br><br>Non-statistical assessment of the degree to which the COA represents all aspects of the COI it is intended to measure. It includes consideration of the development and selection of items, domains and corresponding response options and of their appropriateness and breadth to the COA's intended measurement concept, population, and use. It is typically determined by reviewing the systematic, qualitative studies, e.g., patient/caregiver focus groups and/or expert panels, which informed the development of the conceptual framework that underlies the COA and/or the derivation of the items that comprise the COA. Inclusion of the appropriate stakeholders (e.g. content experts, clinicians, and patients) in the development of the COA is also evaluated.	**A. Has the content validity of the COA been tested? That is, have the items or questions in the COA been determined to have adequate coverage across all relevant facets of the COI being measured for the intended study population and purpose of use? <<Yes/No>>**<br>*Guidance: Review the documentation on the development of this COA to determine if its Content Validity has been assessed. Note that a statement regarding the inclusion of relevant stakeholders in the development of the COA is strong support for the COAs content validity. For Patient-Reported Outcomes (PROs), in particular, the inclusion of patients in their development is strongly advised. If there is no evidence to suggest that the appropriate stakeholders have been included in the development of the COA, then the COA's content validity should be questioned and a NO response should be strongly considered.*<br><br>***If no, specify your rationale:***<br><br>**B. Does the COA exhibit adequate content validity for the COU (i.e., COI, Population and Purpose of Use) specified in the evidence question in Step I of the EB-COP? <<Yes/No>>**<br>*Guidance: Review the questions/items of the COA and consider whether it is appropriate for your COI in your Population and for your Purpose of Use.*<br><br>***If no, please explain why you have determined that the COA does not exhibit adequate content validity for your pre-specified COU:***<br><br>**C. Is the range of answers to the questions appropriate to the purpose of the COA (for e.g., dichotomous answers may be sufficient for diagnostic/discriminative purposes but a greater range may be more appropriate for evaluative)? <<Yes/No>>**<br><br>***If no, specify your rationale:***<br><br>**D. Are the answers or categories mutually exclusive? <<Yes/No>>**<br><br>***If no, specify your rationale:***

Fundamental Quality Indicator	Judgment Criteria
**6) Face Validity**<br><br>Subjective assessment of the extent to which the COA, its items and responses *appear* (at 'face value') to be adequate and appropriate to the COA's intended measurement concept, population and use. Formal assessment may involve asking people – typically, non-experts – to rate the suitability of the COA to its purpose, as it appears to them. A reasonable level of agreement of suitability should be reached. This property is important for widespread acceptance and uptake.	**A. Does the COA appear to measure what it intends to? <<Yes/No>>**<br><br>***If no, specify your rationale:***<br><br>**B. Are the questions/items in the COA clearly worded and easy to understand? <<Yes/No>>**<br>*<u>Guidance</u>: Review the questions/items making up the COA and assess their general readability. Consider whether the questions/items are easy to understand and make sense to the TBI population for which they are intended.*<br><br>***If no, specify your rationale:***<br><br>**C. Are the response options appropriate to questions/items being asked? <<Yes/No>>**<br>*<u>Guidance</u>: Review the questions/items in the COA and their respective response options. Determine whether the response options enable a meaningful response to their respective questions. That is, is YES/NO provided for YES-NO questions and a Likert Scale provided for questions asking about extent of agreement/disagreement or level of severity (e.g., Pain VAS).*<br><br>***If no, specify your rationale:***<br><br>**D. If a global/total score is calculated, is the method of obtaining the global/total score appropriate? <<Yes/No/NA>>**<br>*<u>Guidance</u>: Review the scoring instructions and identify how the scores of the individual items are combined into a global/total score. Does it make sense? For example, if the total score of a COA that consists of three domains is generated by adding up the scores of the individual domains, are the number of items in each domain the same or is each domain weighted so that it contributes equally to the total score? If the method of obtaining the scoring approach is difficult to decipher or involves a more complex mathematical approach, have the COA developers provided justification for the approach?*<br><br>***If no, specify your rationale:***

Fundamental Quality Indicator	Judgment Criteria
**7) Feasibility**<br><br>Subjective assessment of acceptability and feasibility of the administration and processing of the COA. Involves determination of established, standardized administration and scoring procedures and training materials and consideration of respondent and administrative burden given the intended Population, COI and PoU, including completion time, comprehensibility, legibility, availability of language translations and/or culturally-adapted versions for multi-geographic use (if applicable) and availability of alternate forms for the attenuation of practice effects (if applicable).	**A. Are there established standardized administration and scoring procedures and training materials? <<Yes/No>>**<br><br>**B. Are the instructions for administration and scoring and training materials clearly worded and easy to understand? <<Yes/No>>**<br><br>***If no, specify your rationale:***<br><br>**C. Is the COA available in the desired language(s)? <<Yes/No>>**<br><br>***Please specify the language(s) required for your COU:***<br><br>*If only the original COA is required, then it is not relevant to assess its cross-cultural validity in Step IV-D of the EB-COP. However, if a translated version of the original COA is required for use within the pre-specified COU, then the translated version must undergo the full EB-COP review, including an assessment of its cross-cultural validity relative to the original version in Step IV-D of the EB-COP.*<br><br>**D. Is the COA under review culturally acceptable to the Population of interest, or is an acceptable culturally-adapted version of the COA available for use? <<Yes/No>>**<br><br>***Please specify the adaptions required for your COU:***<br><br>*If only the original COA is required, then the prior evaluation of its Content Validity may suffice, particularly if members of the Population of interest were involved in its development. Otherwise, a deeper exploration of the COA may be required to ensure that the questions or items comprising the COA are culturally appropriate to the Population(s) of interest. If a culturally-adapted version exists and is required for use in the pre-specified COU, then the adapted version must undergo the full EB-COP review, including assessment of its cross-cultural validity relative to the original version in Step IV-D of the EB-COP.*

E. Is the administration format/mode (e.g. patient-reported vs in-person interview) and time appropriate for the pre-specified PoU? <<Yes/No>>

> ***If no, please explain why the administration format/mode and/or time were not appropriate:***

F. Is the administration format/mode (e.g. patient-reported vs in-person interview) and time appropriate for the pre-specified Population? <<Yes/No>>

> ***If no, please explain why the administration format/mode and/or time were not appropriate:***

G. If appropriate (see Guidance) and if needed for the pre-specified COU, does an appropriate alternate/parallel form for your COA exist? <<Yes/No/NA>>

> *<u>Guidance</u>**:** For the COU, including time-frame in which you are interested, please consider if a risk of a practice effect exists with the current COA, such that an alternate/parallel form is needed to help attenuate this risk. If an alternate form is required, but does not exist for the current COA, it is not worthwhile pursuing this COA for review. If an alternate form exists and will be used in the pre-specified COU, then the alternate/parallel-forms reliability [QI16] of this adapted version should be evaluated for the Grading and Development of Recommendations for COAs at STEP VI of the EB-COP. Please select N/A if an alternate/parallel form is not required for your COA/COU.*

Fundamental Quality Indicator	Judgment Criteria
**8) Missingness in the context of the COA**<br><br>The identification and reporting of the number of missing responses/items generated by a COA as an indicator of data quality. A high percentage (≥20%) of missing items/responses of COA represents poor data quality and may be an indication of inappropriate content, poor readability and/or lack of feasibility in a particular Population.	**A. Was the number or percentage of missing items/responses described in the available evidence on the development of the COA? [If NO, the review will not be terminated.] <<Yes/No>>**<br><br>**B. If yes, were the data on missing items/responses obtained from the population of interest (e.g., TBI)? [If NO, the review will not be terminated.] <<Yes/No/NA>>**<br><br>**C. If yes to B, was the number of missing items/responses in the TBI population <20%? <<Yes/No>>**

Concluding Prompt	Judgment Criteria
**CONCLUDING PROMPT**	**This ends the assessment of the Fundamental QIs for your COA and, in turn, Step II of the EB-COP.**<br><br>**Before proceeding to Step III, is there any other evidence to suggest that this COA fails to meet any of the previous Fundamental Quality Indicators, rendering it inappropriate in terms of relevance, content and feasibility to the COU (i.e., Population, COI and PoU) specified in Step I? <<YES/NO>>**<br>*Guidance*: *The review should only be discontinued if the COA does not meet the Fundamental QIs. This final question offers you the opportunity to address an aspect of the Fundamental Qis that may not have been covered by the preceding prompts.*<br><br>***If yes, please describe this evidence and your rationale:***

***Step III*: Perform Systematic Literature Search**
(Which relevant, high-quality studies have assessed the COA in/for the intended Context of Use?)

In Step III, you will be performing a comprehensive search of the world literature to identify studies that have evaluated the relevant psychometric properties, i.e., quality indicators, of your COA. Step III represents the first step in identifying the relevant literature for evaluating the COA's ability to measure the concept of interest (COI) in a particular TBI population (P) for a particular Purpose of Use (PoU), as specified in the evidence question. It involves developing a search strategy that is sensitive and specific and searching multiple databases. For guidance on how to conduct the systematic literature review, please consult the 2017 edition of the American Academy of Neurology Clinical Practice Guideline Process Manual:[2] https://www.aan.com/siteassets/home-page/policy-and-guidelines/guidelines/about-guidelines/17guidelineprocman_pg.pdf

1. Develop a search strategy that consists of collections of terms for the following characteristics:
- Population
- Concept of Interest (COI)
- Quality Indicators (QIs or measurement properties)*

NOTE: The PoU is not explicitly included but represented by the profile of QIs that need to be identified as 'adequate' to achieve a full recommendation (in Step VI).

*Filters such as those provided by the COSMIN group[3] on https://cosmin.nl/wp-content/uploads/sensitief-zoekfilter.docx and https://www.cosmin.nl/wp-content/uploads/COSMIN-syst-review-for-PROMs-manual_version-1_feb-2018-1.pdf may be adapted and used to help identify those articles that assess the measurement properties of the COA.

2. Search at least two databases (as recommended by AAN), which may include OVID MEDLINE OVID, EMBASE and PsycINFO, EBSCO CINAHL and SCOPUS.

3. Limit articles by excluding dissertations, book chapters, conference proceedings and case studies.

***Step IV*: Assess the Relevance and Methodological Quality of the Studies Investigating the COA**
(Which relevant, high-quality studies have assessed the COA in/for the intended Context of Use?)

In Step IV, you will use a multi-staged approach to review the articles identified in Step III (Systematic Literature Search). Studies eligible for inclusion must be high-quality according to data quality standards similar to those outlined in the AAN's Clinical Practice Guidelines[2] and COSMIN checklist,[3] among others. The approach to filtering studies is strategically ordered to enable a top-down review that assesses increasingly more granular aspects of the study. Substeps A–D, as below, begin with a general screen of abstracts followed by a review full-text articles with increasing granularity. Studies meeting inclusion will be those evaluating the quality indicators upon which the COA will be graded in the final step (VI).

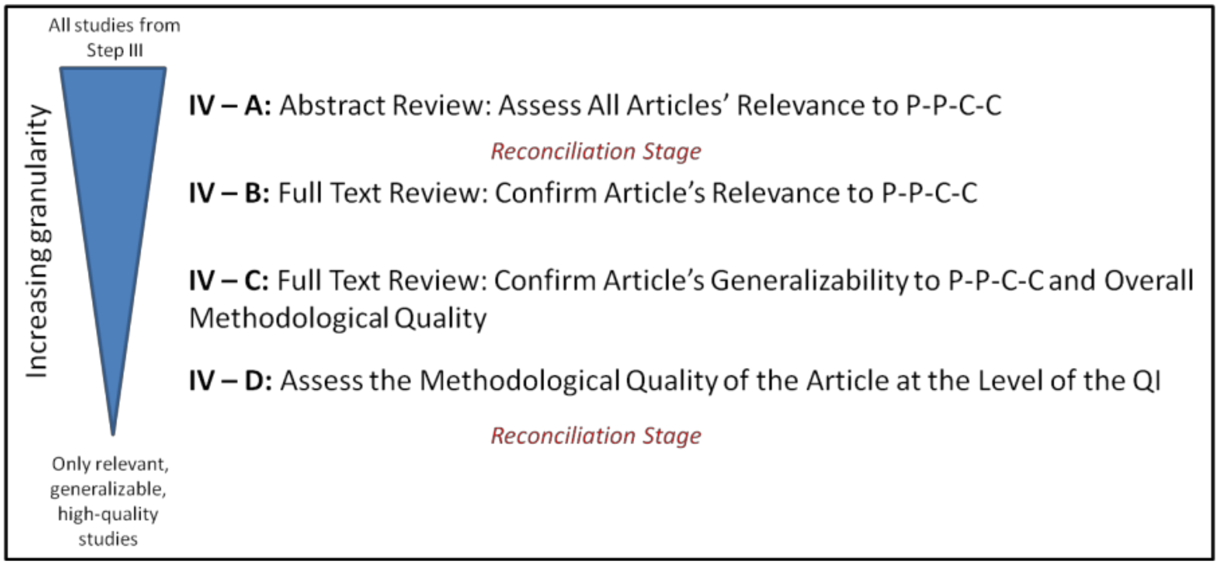

Two independent reviewers are required to complete Step IV-A and Steps IV-B–D iteratively for each article identified in the literature search (Step III). Each time, you will be asked to enter your name or unique initials/identification as well as the article identifying information (primary author, publication year, and citation). It is important to make sure you and your co-reviewer are identifying articles in the survey using the same format, i.e. you both are pasting the complete full citation in the same way.

Questions regarding inclusion/exclusion are organized into sub-steps (following which you will have a chance to review your responses). You may enter any comments or notes in the space provided on each page. Studies will be recommended for exclusion from the review if they do not meet all the criteria presented in each sub-step, and you will be redirected to the beginning of the form to start reviewing the next article.

The final decision to exclude the abstract (Step IV-A) or full-text article (Steps IV-B–D) will be made following comparison and reconciliation with your co-reviewer. The EB-COP administrator will email you the results once the other reviewer has finished and the two reviews have been compiled. If you and your co-reviewer have discrepancies regarding whether to bring an article forward, these should be resolved prior to proceeding to Step IV-B and Step V, respectively. If the two reviewers are unable to reconcile their responses, a third reviewer may be needed to break ties.

Step IV-A: **Abstract Review: Assess All Articles' Relevance to the P-P-C-C and Sample Size**

In Step IV-A, you will answer a short series of questions about each abstract that was retrieved. The aim is to broadly characterize the article's relevance to the P-P-C-C evidence question established in Step I. This will determine which will be brought forward for full-text review. You will assess whether the article A) examines a TBI population in general, B) addresses the chosen age group, C) has adequate sample size, and D) investigates a psychometric property or scoring characteristic of the COA. Should your age criteria change, you will be able to refer back to the second question (B). In the third question (C), you must assess if there are at least 25 appropriately aged participants with TBI in the starting sample (independent of the control group size, if one exists). This question is important when considering the power and generalizability of the study. The 25 number was chosen such that even with up to 80% loss to follow-up, the final sample size would still be 20. The fourth question (D) excludes studies that simply use the COA assuming it is valid and reliable without specifically examining its properties, per COSMIN guidelines.[3]

You may elect to complete the form again for another abstract or end the session and finish at a later time. When you have indicated that abstract review is complete, you will be asked for your email address.

(If the response to any of these questions is "No", the article will be excluded from review, pending consensus between the two independent reviewers. If the answer is unclear, the response "Not Stated" should be chosen, and the article may still be included.)

A. Does this study address a TBI population? <<Yes/No/Not Stated>>

B. Does this study address the age group of interest? <<Yes/No/Not Stated>>

C. Does this study have a sample size of at least 25 subjects that broadly match the population AND age group of interest? <<Yes/No/Not Stated>>

D. Does this study assess/investigate the properties and/or performance of the COA of interest? <<Yes/No/Not Stated>>

> Guidance*: The purpose of this question is to exclude articles that only use the COA to measure an effect or outcome as part of a trial or observation study but do not assess any of its psychometric properties or performance quality indicators.*

***Step IV-B:* Full-text Review: Confirm Relevance to P-P-C-C and Sample Size**

Steps IV-B–D evaluate the relevance and sample size, generalizability, and methodological quality of studies screened for inclusion after reconciliation of Step IV-A. In the online form, you will be asked to select the PoU from your P-P-C-C question in Step 1. Ensure you have the correct PoU at the beginning of the form, as this cannot be changed in later sub-steps. If appropriate, you will extract relevant information about the study characteristics as well as PoU-specific psychometric properties (i.e., quality indicators) from the full text. After reconciliation of full-text articles identified by you and your co-reviewer in this process, those studies deemed relevant and high quality will be brought forward to subsequent steps (V and VI) where they will be used for the grading and development of recommendations for your COA. Note that when using this online form, your progress is cached. Should you need to restart the survey, please clear your browser's cookies or open a private browsing window.

(If the response is "No" or "Not Stated" to any of these questions, the article will be excluded from review, pending consensus between the two independent reviewers.)

A. Is this an original research study? <<Yes/No>>

Select "No" if, for example, the article is an editorial or commentary of an original research study or a review of studies. If the original study described in this article seems relevant, confirm that it is or include it among your articles to review.

B. Does this study address the TBI Population of interest (i.e., at least with respect to age, severity, and chronicity)? <<Yes/No/Not Stated>>

C. Does this study address the COA of interest? <<Yes/No/Not Stated>>

D. Select the PoU from your P-P-C-C question in Step 1 to enable the relevant list of additional QIs for consideration. A table of all QIs associated with each of the 6 PoUs is on the following page. Similar to the fundamental QIs in Step II, following 3 rounds of a modified delphi voting process, these QIs were determined to be important for the grading and development of recommendations for your COA (Step VI of the EB-COP).

**PoU1:** Accurately Diagnose TBI
**PoU2:** Detect TBI Sequelae
**PoU3:** Stratify TBI Subgroups
**PoU4:** Predict TBI Outcome
**PoU5:** Identify Natural History Changes
**PoU6:** Detect Treatment Effects

E. Does the study investigate one or more of the *PoU-specific* quality indicators for the COA under review? <<Yes/No>>

(User is shown list of QIs that are Mandatory or Relevant to preselected PoU as outlined below.)

F. Were the statistical methods used in this study based on Classical Test Theory (CTT) or Item Response Theory (IRT)/Rasch analysis approach or both? <<CTT/IRT/Both>>

G. Does this study include the minimum sample size of subjects that match the TBI Population of interest (i.e., 25 for CTT and 50 for IRT)? <<Yes/No/Not Stated>>

Quality Indicators for Grading and Development of Recommendations for COAs (EB-COP STEP VI)	Accurately Diagnose TBI	Detect TBI Sequelae	Stratify TBI Subgroups	Prognosis of Outcome	Detect Natural History Changes	Detect Treatment Effects
Internal Consistency	Green	Green	Green	Green	Green	Green
Test-Retest Reliability (cross-sectional)	Green	Green	Green	Green	Green	Green
Test-Retest Reliability (longitudinal)	Purple	Purple	Purple	Green	Green	Green
Inter-Rater Reliability (cross-sectional)	Green	Green	Green	Green	Green	Green
Inter-Rater Reliability (longitudinal)	Purple	Purple	Purple	Green	Green	Green
Intra-Rater Reliability (cross-sectional)	Purple	Purple	Purple	Purple	Purple	Purple
Intra-Rater Reliability (longitudinal)	Purple	Purple	Purple	Purple	Purple	Purple
Alternate/Parallel-Forms Reliability (cross-sectional)	Green	Green	Green	Green	Green	Green
Alternate/Parallel-Forms Reliability (longitudinal)	Purple	Purple	Purple	Green	Green	Green
Concurrent Validity	Green	Green	Green	Green	Green	Green
Predictive Validity	Green	Green	Green	Green	Green	Green
Convergent Validity	Purple	Green	Green	Green	Green	Green
Divergent or Discriminant Validity	Purple	Purple	Purple	Purple	Purple	Purple
Known/Contrasted Groups Validity	Purple	Purple	Purple	Purple	Purple	Purple
Internal Construct Validity – Unidimensionality	Green	Green	Green	Green	Green	Green
Internal Construct Validity (Monotonicity/Scalability/Linearity, Invariant Item Ordering, and Local Independence)	Purple	Green	Purple	Purple	Green	Green
Ecologic Validity	Red	Red	Red	Red	Red	Purple
Cross-Cultural Validity	Green	Green	Green	Green	Green	Green
Diagnostic Validity/Accuracy	Green	Purple	Green	Red	Red	Red
Diagnostic Cut-off Score	Green	Purple	Green	Red	Red	Red
Prognostic Validity/Accuracy	Red	Red	Green	Green	Red	Red
Prognostic Cut-off Score	Red	Red	Green	Green	Red	Red
Internal Responsiveness	Red	Red	Red	Red	Purple	Purple
Minimal (Statistically) Important Difference (MID)	Red	Red	Red	Red	Purple	Purple
External Responsiveness	Red	Red	Red	Red	Green	Green
Minimum Clinically Important Difference (MCID)	Red	Red	Red	Red	Green	Green
Normative Values (reference values from a relevant population)	Purple	Green	Purple	Purple	Purple	Purple
Score Variability and Floor and Ceiling Effects	Purple	Green	Purple	Purple	Green	Green

Colors indicate whether the QI is mandatory (green), non-mandatory but should be reported if studied (purple), or not relevant to a specific PoU (red).

Step IV-C: **Full-text Review: Confirm Article's Generalizability to P-P-C-C and Overall Methodological Quality**

(If the response is "No" or "Not Stated" to any of questions A–D or "Yes" to question E, the article will be excluded from review, pending consensus between the two independent reviewers.)

A. Was the TBI sample recruited via a random or consecutive (but NOT convenience) approach? <<Yes/No/Not Stated>>

(Retrospective studies do not need to be excluded if a study dataset that was collected prospectively is utilized.)

B. Was a broad spectrum sample of individuals from the TBI population of interest recruited? That is, if the Population of interest involved an adult (≥18 years of age) TBI population, were individuals covering the full range of the adult age spectrum included? <<Yes/No/Not Stated>>

C. If a mixed sample of individuals was included, was the data of ≥20 (e.g., CTT) and/or ≥40 (e.g., IRT) samples from the TBI Population of interest extractable for a sub-analysis, or did individuals from the TBI Population of interest constitute ≥80% of the total sample of persons with TBI? The Population of interest refers to that specified in P-P-C-C with respect to age, severity, chronicity and/or other such defining factors. <<Yes/No/Not Stated>>

D. Was the percentage of missing items/responses <20%? <<Yes/No/Not Stated>>

(If authors did not comment on missed items, then select "No/Not Stated".)

E. Is there evidence to suggest that the COA was not administered in accord with the guidelines for the administration described in the COA instructions or manual? <<Yes/No>>

Step IV-D: **Full-text Review: Assess the Methodological Quality of the Article at the Level of the QI**

For each study, you will enter basic information including: sample size of the Population of interest represented in the study (not to include controls), age (mean, median, range), frequency of males in the sample, other relevant characteristics (e.g., ethnicity, language), specific COI the study is measuring, and COA administration (e.g., in person, over the phone, by proxy). You will also indicate the severity of TBI, chronicity, and study setting (emergency department, intensive care unit, outpatient clinic, sports, home care, community residential, military, not specified, other). You will then be presented with a list of QIs and asked to choose the ones evaluated by the article under review, specific to your PoU. If multiple QIs were evaluated by the study, select all (this can be done by holding control and clicking more than one).

You will be prompted to answer questions surrounding QI-specific Methodological Quality Standards (MQS), which are presented below. Your responses will determine whether the article has sufficiently high quality. Articles must meet all of the MQS for mandatory QIs to be included in data analysis and synthesis in subsequent steps (V and VI). When mandatory criteria are not met for a QI under review, you will be notified that the article's methodological quality is insufficient. Refer to the color-coded table above for QIs deemed mandatory (per a modified delphi consensus-building process) for each PoU. This is an iterative process such that when you have indicated that all QIs addressed by the article have been evaluated, you will be asked if you would like to review another article. You may elect to complete the form again for another article or end the session and finish at a later time. Following this substep, your responses for Steps IV-B–D will be reconciled with your co-reviewer's.

(User will be asked to select the PoU-specific QIs under investigation in the study, and will only be shown forms for the PoU-specific QIs selected.)

RELIABILITY

Involves assessing the reproducibility or stability of the COA at a particular point in time or within a relatively short time interval (cross-sectional), and/or over a longer period of time (longitudinal). Various types exist, including internal consistency, test-retest reliability, inter-rater reliability, intra-rater reliability and alternate/parallel-forms reliability.

Quality Indicator (QI)	QI-Specific MQS Prompts
**Reliability: Internal Consistency**<br><br>For multi-item COAs, the extent to which the items making up the COA scale or subscale correlate with the other items making up the scale or subscale and with the total score. It is typically evaluated by Cronbach's alpha, Kuder-Richardson formula 20 (KR-20), average inter-item and item-total correlations, split-half reliability coefficient or the Spearman-Brown formula. It can also be evaluated via Rasch analysis (i.e., Person Separation Index (PSI)). In general, evaluation of internal consistency is only meaningful in COAs that are unidimensional (i.e., expected to measure only one construct). NB: Internal consistency and unidimensionality are not the same thing. Unidimensionality is considered separately as a QI in the EB-COP.	**A. Was there a clear hypothesis about the expected correlation between all the items in the measure and each of the subscales, or between each subscale? <<YES/NO>>**<br><br>***If yes, specify the hypothesis(es):***<br><br>**B. Was internal consistency tested between the total score and the subscale scores, and/or between the subscale scores? <<YES/NO>>**<br><br>**C. Were the statistical approaches used to evaluate this QI clearly reported? <<YES/NO>**<br>*(e.g., refer to description of internal consistency above for examples of these approaches.)*<br><br>**D. Were the statistics appropriate relative to the data produced by the COA? <<YES/NO/I don't know>>**<br>*(e.g., **for CTT**: Interval data – Split-half; Cronbach's alpha, Pearson/Spearman Correlation Coefficient; dichotomous – Cronbach's alpha, KR-20. Please note that for Cronbach's alpha to be accurate, the (sub)scale should consist of at least four items. **For IRT**: global goodness of fit statistic, PSI. For IRT, the assumptions (e.g., unidimensionality, local independence, and item fit) for estimating the parameters of the IRT should be checked. Please ensure you have selected IRT assumptions among your list of QIs. If you are unsure, seek statistical guidance prior to using the evidence.)*<br><br>***Specify data type produced by COA (interval, ordinal, nominal):***<br><br>***Specify statistic(s) used:***

	Specify statistic(s) result (including 95% confidence intervals, if available):<br><br>**E. Was there any other evidence to suggest that there were flaws in the study design, methods or statistical analysis for this QI, rendering the findings unreliable? <<YES/NO>>**<br><br>***If yes, please describe this evidence and where it appears in the text (e.g., page and line number):***<br><br>**F. Summary and Comments (optional):**<br>*(Use this space to summarize the study findings and add comments that may be helpful for reconciling discrepancies and analyzing the evidence downstream.)*

Quality Indicator (QI)	QI-Specific MQS Prompts
**Reliability: Test-Retest Reliability (cross-sectional)**<br><br>The extent to which the COA remains stable or consistent over repeated administrations delivered over a short time interval (e.g., hours or days). Requires consideration of appropriate time interval between measurements to ensure clinical stability with respect to the COI in the relevant Population and minimal memory effects, if applicable. It is typically calculated using the intraclass correlation coefficient (ICC), (weighted) Cohen's Kappa or Pearson's and Spearman's rank correlation coefficients, depending on data type and distribution.	**A. Were at least two, independent administrations of the COA by the same rater available? <<YES/NO>>**<br>*(Here, independence refers to completely separate, objective administrations of the COA by the same rater.)*<br><br>**B. Were at least two scores available for ≥80% of sample? <<YES/NO>>**<br><br>**C. Was the time interval between administrations stated? <<YES/NO>>**<br><br>***Specify time interval:***<br><br>**D. Was the time interval between measurements appropriate? <<YES/NO>>**<br>*(i.e., patients were stable and practice effects mitigated based on your understanding of the concept of interest and/or the justification provided in the study)*<br><br>**E. Did the conditions for the COA administration (e.g., environment, instructions) appear consistent between the two measurements? <<YES/NO>>**<br><br>**F. Was there a clear hypothesis about the expected level of agreement or correlation (e.g., ≥0.70) between the independent administrations? <<YES/NO>>**<br><br>***Specify the hypothesis:***<br><br>**G. Was the statistical approach used to evaluate this QI clearly reported? <<YES/NO>>**<br>*(e.g., refer to description of cross-sectional test-retest reliability on the left for examples of these approaches.)*<br><br>**H. Were the statistics appropriate relative to the data produced by the COA? <<YES/NO/I don't know>>**<br>*(e.g., for nominal/dichotomous - (weighted) Cohen's kappa; for normally distributed interval data) - ICC or Pearson's correlation + Wilcoxon signed rank, Bland-Altman plot or other for systematic*

	error; for skewed/non-normally distributed data - Spearman.'s rank correlation + test of systematic error. If you are unsure, seek statistical guidance prior to using the evidence.)<br><br>***Specify data type produced by COA (interval, ordinal, nominal):***<br><br>***Specify statistic(s) used:***<br><br>***Specify statistic(s) result (including 95% confidence intervals, if available):***<br><br>**I. Was there any other evidence to suggest that there were flaws in the study design, methods or statistical analysis for this QI, rendering the findings unreliable? <<YES/NO>>**<br><br>***If yes, describe this evidence and where it appears in the text (e.g., page and line number):***<br><br>**J. Summary and Comments (optional):**<br>*(Use this space to summarize the study findings and add comments that may be helpful for reconciling discrepancies and analyzing the evidence downstream.)*

Quality Indicator (QI)	QI-Specific MQS Prompts
**Reliability: Test-Retest Reliability (longitudinal)**<br><br>The extent to which the COA remains stable or consistent over repeated administrations delivered over a longer time interval during which change may occur, but is tested on individuals who are clinically stable. It can be calculated using the intraclass correlation coefficient (ICC), (weighted) Cohen's Kappa or Pearson's and Spearman's rank correlation coefficients, depending on data type and distribution. Longitudinal statistical models, such as autoregressive, trait-state, growth curve and Rasch models, are used when change may have occurred across the sample tested but the rank order is expected to be preserved.	**A. Were at least two, independent administrations of the COA by the same rater available? <<YES/NO>>**<br>*(Here, independence refers to completely separate, objective administrations of the COA by the same rater.)*<br><br>**B. Were at least two scores available for ≥80% of sample? <<YES/NO>>**<br><br>**C. Was the time interval between administrations stated? <<YES/NO>>**<br><br>***Specify time interval:***<br><br>**D. Did the authors confirm that the patients studied were stable or acknowledge that appropriate statistics were used to account for any changes? <<YES/NO>>**<br><br>**E. Did the conditions for the COA administration (e.g., environment, instructions) appear consistent between the two measurements? <<YES/NO>>**<br><br>**F. Was there a clear hypothesis about the expected level of agreement or correlation (e.g., ≥0.70) between the independent administrations? <<YES/NO>>**<br><br>**G. Were the statistical approaches used to evaluate this QI clearly reported? <<YES/NO>>**<br>*(e.g., refer to description of longitudinal test-retest reliability to the left for examples of these approaches.)*<br><br>**H. Were the statistics appropriate relative to the data produced by the COA? <<YES/NO/I don't know>>**<br>*(e.g., for nominal/dichotomous - (weighted) Cohen's kappa; for normally distributed interval data – ICC or Pearson's correlation + Wilcoxon signed rank, Bland-Altman plot or other for systematic error; for skewed/non-normally distributed data - Spearman's rank correlation + test of systematic error. Other, longitudinal models that allow for change in the patients may also be appropriate. If you are unsure, seek statistical guidance prior to using the evidence.)*

	Specify data type produced by COA (interval, ordinal, nominal):<br><br>***Specify statistic(s) used:***<br><br>***Specify statistic(s) result (including 95% confidence intervals, if available):***<br><br>**I. Was there any other evidence to suggest that there were flaws in the study design, methods or statistical analysis for this QI, rendering the findings unreliable? <<YES/NO>>**<br><br>***If yes, describe this evidence:***<br><br>**J. Summary and Comments (optional):**<br>*(Use this space to summarize the study findings and add comments that may be helpful for reconciling discrepancies and analyzing the evidence downstream.)*

Quality Indicator (QI)	QI-Specific MQS Prompts
**Reliability: Inter-Rater Reliability (cross-sectional)**<br><br>The extent to which administrations by independent raters of a COA agree when measured at a particular point in time. It is typically calculated using intraclass correlation coefficient (ICC), (weighted) Cohen's Kappa or Pearson's and Spearman's rank correlation coefficients, depending on data type and distribution.	**A. Were at least two independent administrations of the COA by two different raters available? <<YES/NO>>**<br>*(Here, to qualify as 'independent', the raters should have been blinded/masked to the others' results.)*<br><br>**B. Were at least two scores available for ≥80% of sample? <<YES/NO>>**<br><br>**C. Were the two administrations conducted within a short period of time (i.e., concurrently) to increase the likelihood that the patient remained stable between measurements? <<YES/NO>>**<br><br>***Specify time interval:***<br><br>**D. Did the conditions for the COA administration (e.g., environment, instructions) appear consistent between the two measurements? <<YES/NO>>**<br><br>**E. Was there a clear hypothesis about the expected level of agreement or correlation (e.g., ≥0.70) between the independent administrations? <<YES/NO>>**<br><br>**F. Were the statistical approaches used to evaluate this QI clearly reported? <<YES/NO>>**<br>*(e.g., refer to description of cross-sectional inter-rater reliability to the left for examples of these approaches.)*<br><br>**G. Were the statistics appropriate to the distribution/type of data produced by the COA? <<YES/NO/I don't know>>**<br>*(e.g., for nominal/dichotomous - (weighted) Cohen's kappa; for normally distributed interval data) - ICC or Pearson's correlation + Wilcoxon signed rank, Bland-Altman plot or other for systematic error; for skewed/non-normally distributed data - Spearman's rank correlation + test of systematic error. If you are unsure, seek statistical guidance prior to using the evidence.)*<br><br>***Specify statistic(s) used:***

Specify statistic(s) result (including 95% confidence intervals, if available):

H. Was there any other evidence to suggest that there were flaws in the study design, methods or statistical analysis for this QI, rendering the findings unreliable? <<YES/NO>>

If yes, please describe this evidence and where it appears in the text (e.g., page and line number):

I. Summary and Comments (optional):

(Use this space to summarize the study findings and add comments that may be helpful for reconciling discrepancies and analyzing the evidence downstream.)

Quality Indicator (QI)	QI-Specific MQS Prompts
**Reliability: Inter-Rater Reliability (longitudinal)**<br><br>The extent to which administrations by independent raters of a COA agree when administered on multiple occasions over a longer period time in which change may have occurred, but is tested in individuals who are clinically stable. In particular, assessment of longitudinal inter-rater reliability helps to monitor ‘rater drift’ or changes in rater behavior (e.g., instruction, cueing/feedback and interpretation) over administrations and time. It can be calculated using the intraclass correlation coefficient (ICC), (weighted) Cohen’s Kappa or Pearson's and Spearman's rank correlation coefficients, depending on data type and distribution. Longitudinal models, such as autoregressive, trait-state, growth curve and Rasch models, are used when change may have occurred across the sample tested but the rank order is expected to be preserved.	**A. Were at least two, independent administrations of the COA available? <<YES/NO>>**<br>*(Here, to qualify as ‘independent’, the raters should have been blinded/masked to the others’ results.)*<br><br>**B. Were at least two scores available for ≥80% of sample? <<YES/NO>>**<br><br>**C. Was the time interval between administrations stated? <<YES/NO>>**<br><br>***Specify time interval:***<br><br>**D. Did the authors confirm that the patients were stable in the interim or acknowledge that appropriate statistics were used to account for any changes? <<YES/NO>>**<br><br>**E. Did the conditions for the COA administration (e.g., environment, instructions) appear consistent between the two measurements? <<YES/NO>>**<br><br>**F. Was there a clear hypothesis about the expected level of agreement or correlation (e.g., ≥0.70) between the independent administrations? <<YES/NO>>**<br><br>***Specify the hypothesis:***<br><br>**G. Were the statistical approaches used to evaluate this QI clearly reported? <<YES/NO>>**<br>*(e.g., refer to description of longitudinal inter-rater reliability to the left for examples of these approaches.)*<br><br>**H. Were the statistics appropriate to the distribution/type of data produced by the COA? <<YES/NO/I don’t know>>**<br>*(e.g., for nominal/dichotomous - (weighted) Cohen's kappa; for normally distributed interval data) - ICC or Pearson’s correlation + Wilcoxon signed rank, Bland-Altman plot or other for systematic error; for skewed/non-normally distributed data - Spearman’s rank correlation + test of systematic error. Other, longitudinal models that allow for change in the patients may also be appropriate. If you are unsure, seek statistical guidance prior to using the evidence.)*

	Specify statistic(s) used:<br><br>***Specify statistic(s) result (including 95% confidence intervals, if available):***<br><br>**I. Was there any other evidence to suggest that there were flaws in the study design, methods or statistical analysis for this QI, rendering the findings unreliable? <<YES/NO>>**<br><br>***If yes, describe this evidence and where it appears in the text (e.g., page and line number):***<br><br>**J. Summary and Comments (optional):**<br>*(Use this space to summarize the study findings and add comments that may be helpful for reconciling discrepancies and analyzing the evidence downstream.)*

Quality Indicator (QI)	QI-Specific MQS Prompts
**Reliability: Intra-Rater Reliability (cross-sectional)**<br><br>The extent to which independent administrations by the same rater of a COA agree when measured at a particular time. It aims to assess whether the COA is amenable to consistent administrations by the same rater. It is typically calculated using intraclass correlation coefficient (ICC), (weighted) Cohen's Kappa or Pearson's and Spearman's rank correlation coefficients, depending on data type and distribution. It is often difficult to separate it from test-retest reliability.	**A. Were at least two, independent administrations of the COA by the same rater available? <<YES/NO>>**<br><br>**B. Were at least two scores available for ≥80% of sample? <<YES/NO>>**<br><br>**C. Was the time interval between administrations stated? <<YES/NO>>**<br><br>***Specify time interval:***<br><br>**D. Was the time interval between administrations appropriate? <<YES/NO>>**<br>*(i.e., patients were stable and practice effects mitigated based on your understanding of the concept of interest and/or the justification provided in the study)*<br><br>**E. Did the conditions for the COA administration (e.g., environment, instructions) appear consistent between the two measurements? <<YES/NO>>**<br><br>**F. Was there a clear hypothesis about the expected level of agreement or correlation (e.g., ≥0.70) between the independent administrations? <<YES/NO>>**<br><br>**G. Were the statistical approaches used to evaluate this QI clearly reported? <<YES/NO>>**<br>*(e.g., refer to description of cross-sectional intra-rater reliability to the left for examples of these approaches.)*<br><br>**H. Were the statistics appropriate to the distribution/type of data produced by the COA? <<YES/NO/I don't know>>**<br>*(e.g., for nominal/dichotomous - (weighted) Cohen's kappa; for normally distributed interval data) - ICC or Pearson's correlation + Wilcoxon signed rank, Bland-Altman plot or other for systematic error; for skewed/non-normally distributed data - Spearman's rank correlation + test of systematic error. If you are unsure, seek statistical guidance prior to using the evidence.)*<br><br>***Specify statistic(s) used:***

	Specify statistic(s) result (including 95% confidence intervals, if available):<br><br>**I. Was there any other evidence to suggest that there were flaws in the study design, methods or statistical analysis for this QI, rendering the findings unreliable? <<YES/NO>>**<br><br>***If yes, describe this evidence:***<br><br>**J. Summary and Comments (optional):**<br>*(Use this space to summarize the study findings and add comments that may be helpful for reconciling discrepancies and analyzing the evidence downstream.)*

Quality Indicator (QI)	QI-Specific MQS Prompts
**Reliability: Intra-Rater Reliability (longitudinal)**<br><br>The extent to which independent administrations by the same rater of a COA agree when measured over a longer period of time when change may have occurred, but is tested in individuals who are clinically stable. It aims to assess whether the COA is amenable to consistent administrations by the same rater. It is typically calculated using intraclass correlation coefficient (ICC), (weighted) Cohen's Kappa or Pearson's and Spearman's rank correlation coefficients, depending on data type and distribution. Longitudinal models, such as autoregressive, trait-state, growth curve and Rasch models, may also be used when change may have occurred across the sample tested but the rank order is expected to be preserved.	**A. Were at least two, independent administrations of the COA by the same rater available? <<YES/NO>>**<br>*(Here, independence refers to completely separate, objective administrations of the COA by the same rater.)*<br><br>**B. Were at least two scores available for ≥80% of sample? <<YES/NO>>**<br><br>**C. Was the time interval between administrations stated? <<YES/NO>>**<br><br>***Specify time interval:***<br><br>**D. Did the authors confirm that the patients were stable in the interim or acknowledge that appropriate statistics were used to account for any changes? <<YES/NO>>**<br><br>**E. Did the conditions for the COA administration (e.g., environment, instructions) appear consistent between the two measurements? <<YES/NO>>**<br><br>**F. Was there a clear hypothesis about the expected level of agreement or correlation (e.g., ≥0.70) between the independent administrations? <<YES/NO>>**<br><br>**G. Were the statistical approaches used to evaluate this QI clearly reported? <<YES/NO>>**<br>*(e.g., refer to description of longitudinal intra-rater reliability to the left for examples of these approaches.)*<br><br>**H. Were the statistics appropriate to the distribution/type of data produced by the COA? <<YES/NO/I don't know>>**<br>*(e.g., for nominal/dichotomous - (weighted) Cohen's kappa; for normally distributed interval data) - ICC or Pearson's correlation + Wilcoxon signed rank, Bland-Altman plot or other for systematic error; for skewed/non-normally distributed data - Spearman's rank correlation + test of systematic error. Other, longitudinal models that allow for change in the patients may also be appropriate. If you are unsure, seek statistical guidance prior to using the evidence.)*

	Specify statistic(s) used:<br><br>***Specify statistic(s) result (including 95% confidence intervals, if available):***<br><br>**I. Was there any other evidence to suggest that there were flaws in the study design, methods or statistical analysis for this QI, rendering the findings unreliable? <<YES/NO>>**<br><br>***If yes, describe this evidence:***<br><br>**J. Summary and Comments (optional):**<br>*(Use this space to summarize the study findings and add comments that may be helpful for reconciling discrepancies and analyzing the evidence downstream.)*

Quality Indicator (QI)	QI-Specific MQS Prompts
**Reliability: Alternate/Parallel-Forms Reliability (cross-sectional)**<br><br>The extent to which parallel forms of a COA correlate with each other when administered at the same point in time. Parallel forms are constructed as equivalent measures (i.e. measuring the same COI in the same way and generating comparable scores) that are intended to be used independently of each other. Examples include the administration of different word lists to test episodic verbal learning and memory with the California Verbal Learning Test (CVLT) or of the Trail Making Test Part A with a different configuration of 25 circles to test for speed of processing or mental flexibility. Two MRI scanners of the same make and model measuring the identical structure may also be viewed as parallel forms. May be evaluated by estimating Cronbach's alpha via a maximum likelihood test.	**A. Was the development of the alternate/parallel form described? <<YES/NO>>**<br><br>**B. Was the content validity of the alternate/parallel form justified? <<YES/NO>>**<br>*(e.g., parallel forms are usually developed using items from the same pool of questions that measure the construct as the original form)*<br><br>**C. Were both forms independently administered to the same group of subjects (i.e. within-subject analysis)? <<YES/NO>>**<br><br>**D. Was the order of administration of the two forms randomized in the same group of subjects (e.g., half started with one form, while the other half started with the other)? <<YES/NO>>**<br><br>**E. Were the scores of both forms available in ≥80% of sample? <<YES/NO>>**<br><br>**F. Were the two administrations conducted within a short period of time (e.g., 24 hours)? <<YES/NO>>**<br>*(i.e., to increase the likelihood that the patient remained stable between measurements)*<br><br>**G. Did the conditions for the COA administration (e.g., environment, instructions) appear consistent between the two measurements? <<YES/NO>>**<br><br>**H. Was there a clear hypothesis about the expected level of agreement or correlation (e.g., ≥0.70) between the two forms? <<YES/NO>>**<br><br>**I. Were the statistical approaches used to evaluate this QI clearly reported? <<YES/NO>>**<br>*(e.g., refer to description of cross-sectional alternate/parallel-form reliability to the left for examples of these approaches.)*<br><br>**J. Were the statistics appropriate to the distribution/type of data produced by the COA? <<YES/NO/I don't know>>**<br>*(If you are unsure, seek statistical guidance prior to using the evidence.)*

Specify statistic(s) used:

Specify statistic(s) result (including 95% confidence intervals, if available):

K. Was there any other evidence to suggest that there were flaws in the study design, methods or statistical analysis for this QI, rendering the findings unreliable? <<YES/NO>>

If yes, describe this evidence and where it appears in the text (e.g., page and line number):

L. Summary and Comments (optional):

(Use this space to summarize the study findings and add comments that may be helpful for reconciling discrepancies and analyzing the evidence downstream.)

Quality Indicator (QI)	QI-Specific MQS Prompts
**Reliability: Alternate/Parallel-Forms Reliability (longitudinal)**<br>The extent to which parallel forms of a COA correlate with each other when administered over a longer period of time in which change may have occurred, but is tested in individuals who are clinically stable. May be evaluated by estimating Cronbach's alpha via a maximum likelihood test.	**A. Was the development of the alternate/parallel form described? <<YES/NO>>**<br><br>**B. Was the content validity of the alternate/parallel form justified? <<YES/NO>>**<br>*(e.g., parallel forms are usually developed using items from the same pool of questions that measure the construct as the original form)*<br><br>**C. Were both forms independently administered to the same group of subjects (i.e. within-subject analysis)? <<YES/NO>>**<br><br>**D. Was the order of administration of the two forms randomized in the same group of subjects (e.g., half started with one form, while the other half started with the other)? <<YES/NO>>**<br><br>**B. Were the scores of both forms available in ≥80% of sample? <<YES/NO>>**<br><br>**C. Was the time interval between administrations stated? <<YES/NO>>**<br><br>**D. Did the authors confirm that the patients studied were stable or acknowledge that appropriate statistics were used to account for any changes? <<YES/NO>>**<br><br>**E. Did the conditions for the COA administration (e.g., environment, instructions) appear consistent between the two measurements? <<YES/NO>>**<br><br>**F. Was there a clear hypothesis about the expected level of agreement or correlation (e.g., ≥0.70) between the two forms? <<YES/NO>>**<br><br>**G. Were the statistical approaches used to evaluate this QI clearly reported? <<YES/NO>>**<br>*(e.g., refer to description of cross-sectional alternate/parallel-form reliability to the left for examples of these approaches.)*<br><br>**H. Were the statistics appropriate to the distribution/type of data produced by the COA? <<YES/NO/I don't know>>**

	(If you are unsure, seek statistical guidance prior to using the evidence.)<br><br>***Specify statistic(s) used:***<br><br>***Specify statistic(s) result (including 95% confidence intervals, if available):***<br><br>**I. Was there any other evidence to suggest that there were flaws in the study design, methods or statistical analysis for this QI, rendering the findings unreliable? <<YES/NO>>**<br><br>***If yes, describe this evidence and where it appears in the text (e.g., page and line number):***<br><br>**J. Summary and Comments (optional):**<br>*(Use this space to summarize the study findings and add comments that may be helpful for reconciling discrepancies and analyzing the evidence downstream.)*

CRITERION VALIDITY

An external measure of validity that aims to evaluate the relationship between the COA and a 'gold' standard. Requires specification of a 'gold' standard, which may include a tool or measurement that is widely accepted as being the best available in the field. Two forms exist: concurrent and predictive validity (see below).

Quality Indicator (QI)	QI-Specific MQS Prompts
**Criterion Validity: Concurrent Validity**<br><br>The extent to which the COA correlates with a field-accepted ‘gold’ or criterion standard that is intended to measure the same or similar construct and administered at the same time. Involves consideration and justification of the ‘gold’ standard. It is typically measured using the correlation coefficient (e.g. Pearson or Spearman's) or the Area Under the Curve (AUC) via Receiver Operating Characteristics (ROC) analysis, upon dichotomization of the ‘gold’ standard.	**A. Was the ‘gold’ or criterion standard adequately described? <<YES/NO>>**<br><br>***Specify ‘gold’/criterion standard:***<br><br>**B. Was the criterion standard employed adequately justified or, in your best judgment, reasonable? <<YES/NO>>**<br><br>***If no, please specify your reasoning:***<br><br>**C. Was there less than 70% item overlap between the COA and its ‘gold’ standard? <<YES/NO>>**<br><br>**D. Were the outcomes of the COA and criterion standard determined independently by raters who were blinded/masked to the outcome of the other assessment? <<YES/NO>>**<br>*(That is, was the individual using the COA blinded to the outcome of the gold standard, and vice versa?)*<br><br>**E. Were outcomes for both the COA and criterion standard available in ≥80% of total sample? <<YES/NO>>**<br><br>**F. Were the two administrations conducted within a short period of time? <<YES/NO>>**<br>*(i.e., to increase the likelihood that the patient remained stable between measurements)*<br><br>**G. Was there a clear hypothesis about the direction and magnitude of correlation (e.g., ≥0.70) expected between the two assessments? <<YES/NO>>**

	If yes, specify the hypothesis:<br><br>**H. Were the statistical approaches used to evaluate this QI clearly reported? <<YES/NO>>**<br>*(e.g., refer to description of concurrent validity above for examples of these approaches.)*<br><br>**I. Were the statistics appropriate to the type of data produced by the COA? <<YES/NO/I don't know>>**<br>*(e.g., Pearson, Spearman, Kendall rank correlation, partial correlation for continuous or categorical; ROC-AUC upon dichotomization of the criterion standard. If you are unsure, seek statistical guidance prior to using the evidence.)*<br><br>***Specify statistic(s) used:***<br><br>***Specify statistic(s) result (including 95% confidence intervals, if available):***<br><br>**J. Was there any other evidence to suggest that there were flaws in the study design, methods or statistical analysis for this QI, rendering the findings unreliable? <<YES/NO>>**<br><br>***If yes, describe this evidence and where it appears in the text (e.g., page and line number):***<br><br>**K. Summary and Comments (optional):**<br>*(Use this space to summarize the study findings and add comments that may be helpful for reconciling discrepancies and analyzing the evidence downstream.)*

Quality Indicator (QI)	QI-Specific MQS Prompts
**Criterion Validity: Predictive Validity**<br><br>The extent to which the COA correlates with a field-accepted ‘gold’ or criterion standard that is intended to measure the same or similar construct and administered at some time in the future. In other words, the extent to which the COA is able to predict the score of this 'gold' standard in the future. For example, the high school grade point average (GPA) is known to have strong predictive validity because it has been found to correlate strongly with students' GPA in college. Evaluation of predictive validity involves consideration and justification of the future 'gold' standard. It is typically measured using the correlation coefficient (e.g. Pearson or Spearman's) or the Area Under the Curve (AUC) via Receiver Operating Characteristics (ROC) analysis, upon dichotomization of the ‘gold’ standard.	**A. Was the ‘gold’ or criterion standard adequately described? <<YES/NO>>**<br><br>***Specify ‘gold’ standard, including time-frame:***<br><br>**B. Was the criterion standard adequately justified or, in your best judgment, reasonable? <<YES/NO>>**<br><br>***If no, please specify your reasoning:***<br><br>**C. Were the outcomes of the COA and criterion standard determined independently by raters who were blinded/masked to the outcome of the other assessment? <<YES/NO>>**<br>*(That is, was the individual using the COA blinded to the outcome of the gold standard, and vice versa?)*<br><br>**D. Were outcomes for both the COA and criterion standard available in ≥80% of total sample? <<YES/NO>>**<br><br>**E. Was there a clear hypothesis about the direction and magnitude of correlation (e.g., ≥0.70) expected between the two assessments? <<YES/NO>>**<br><br>**F. Were the statistical approaches used to evaluate this QI clearly reported? <<YES/NO>>**<br>*(e.g., refer to description of predictive validity to the left for examples of these approaches.)*<br><br>**G. Were the statistics appropriate to the distribution/type of data produced by the COA? <<YES/NO/I don’t know>>**<br><br>***Specify statistic(s) used:***<br><br>***Specify statistic(s) result (including 95% confidence intervals, if available):***<br><br>**H. Was there any other evidence to suggest that there were flaws in the study design, methods or statistical analysis for this QI, rendering the findings unreliable? <<YES/NO>>**

	If yes, describe this evidence and where it appears in the text (e.g., page and line number):<br><br>**I. Summary and Comments (optional):**<br>*(Use this space to summarize the study findings and add comments that may be helpful for reconciling discrepancies and analyzing the evidence downstream.)*

CONSTRUCT VALIDITY

A broad property of measurement instruments that aims to verify that a COA measures the (often intangible) phenomenon, or construct, that it is intended to measure and in the manner that it is expected to measure that construct. Both internal (or 'strong') and external (or 'weak') measures of construct validity exist. Internal construct validity is also referred to as "structural" or "factorial" validity. Assessment of internal construct validity refers to the formal verification of certain pre-specified hypotheses (i.e. a model) regarding the behavior of the COA's composite items and resulting scores, using robust, multi-variate statistical approaches. Confirmation of internal construct validity has become more prevalent with the application of 'modern' approaches to outcome measure development, such as Item Response Theory (IRT) and Rasch analysis. The elements of internal construct validity described in the following QIs are considered critical to COAs and, as such, represent assumptions that underly IRT/Rasch modeling approaches that must be confirmed in the development or assessment of a psychometrically sound COA. While they are most efficiently captured under an IRT model, these properties may also be evaluated using classical test theory (CTT) approaches. Generally, if the exact type of construct validity being reported is not specified, it is most likely convergent validity (a type of external construct validity described below along with divergent/discriminant validity). Traditionally, measures of external construct validity have been the most commonly reported measures of construct validity due to their prevalence in outcome measure development using Classical Test Theory. The FDA define construct validity as "evidence that relationship among items, domains and concepts conform to a priori hypotheses concerning logical relationships that should exist with measures of related concepts or scores produced in similar or diverse patient groups. This form of construct validity has also been referred to as "weak" validity because it is established by correlating it with some external measure.

Quality Indicator (QI)	QI-Specific MQS Prompts
**Unidimensionality**<br>**(via non-IRT-based approaches)**<br><br>Evidence that a COA that results in a *single* (composite/total) score represents a *single* latent construct, namely the single construct that it is proposed to measure. Failure to show unidimensionality suggests that more than one construct is being captured by the COA, complicating the interpretation of the total score. Unidimensionality is assumed when testing the internal consistency or correlation between the items making up	**A. Was the single latent trait that the COA is intended to measure adequately described? <<YES/NO>>**<br><br>**B. Were all of the items of the COA identified as representing the construct? <<YES/NO>>**<br><br>**C. Was unidimensionality assessed using all of the items in the COA? <<YES/NO>>**<br>*(i.e., unidimensionality was assessed using all the items that contribute to the COA's subscale or total scores. Select NO if any items were removed a priori).*<br><br>***If No, indicate which items were removed:***<br><br>**D. Were the statistical approaches used to evaluate this QI clearly reported? <<YES/NO>>**<br>*(e.g., refer to description of unidimensionality above for examples of these approaches.)*

that construct. It is typically assessed via factor analytic techniques, such as confirmatory and exploratory factor analysis, or via IRT/Rasch analysis. It is one of the main assumptions underlying IRT/Rasch models. If IRT/Rasch approaches were applied, complete the MQS prompts for the QI Internal Construct Validity (IRT assumptions), below. May also be referred to as “structural validity”.	**E. Were the statistics appropriate to the distribution/type of data produced by the COA? <<YES/NO/I don’t know>>**<br>*(e.g., for non-IRT-based approaches: exploratory or confirmatory factor analysis, principle component analysis)*<br><br>***Specify statistic(s) used:***<br><br>***Specify statistic(s) result:***<br><br>**F. Was there any other evidence to suggest that there were flaws in the study design, methods or statistical analysis for this QI, rendering the findings unreliable? <<YES/NO>>**<br><br>***If yes, describe this evidence and where it appears in the text (e.g., page and line number):***<br><br>**G. Summary and Comments (optional):**<br>*(Use this space to summarize the study findings and add comments that may be helpful for reconciling discrepancies and analyzing the evidence downstream.)*

Quality Indicator (QI)	QI-Specific MQS Prompts
**Internal Construct Validity (via IRT-based approaches)**<br><br>**Unidimensionality**<br>Evidence that a COA that results in a *single* (composite/total) score represents a *single* latent construct, namely the single construct that it is proposed to measure. Failure to show unidimensionality suggests that more than one construct is being captured by the COA, complicating the interpretation of the total score. Unidimensionality is assumed when testing the internal consistency or correlation between the items making up that construct. It is typically assessed via factor analytic techniques, such as confirmatory and exploratory factor analysis, or via IRT/Rasch analysis. It is one of the main assumptions underlying IRT/Rasch models.<br><br>**Monotonicity (or Scalability)**<br>Evidence that, as one's score on a COA increases, the score on any single item within the COA increases or at least remains stable, representing a S-shaped function for dichotomous responses. For polytomous items, the "category structure" or "threshold ordering" is determined.	**A. Was the single latent trait or construct that the COA is intended to measure adequately described? <<YES/NO>>**<br><br>**B. Were all of the items of the COA identified as relevant to the single latent trait or construct? <<YES/NO>>**<br><br>**C. Were all of the items of the COA included at the start of the IRT/Rasch analysis? <<YES/NO>>**<br>*(Select NO if any items were removed a priori)*<br><br>**D. Was the IRT/Rasch model and/or software used clearly reported? <<YES/NO>>**<br>*(e.g., for dichotomous response options, the dichotomous model; for 3 or more (polytomous) response options (e.g., likert scale), the Andrich Rating Scale Model or Masters Partial Credit Model via eg, RUMM2020 or WINSTEP)*<br><br>**E. Was an evaluation of Person Fit performed for each subject in the analysis? <<YES/NO>>**<br>*(e.g., via chi-square statistics)*<br><br>***Specify statistic(s) used:***<br><br>***Specify the number of persons identified as "mis-fitting" the model:***<br><br>***Specify how these persons were handled:***<br><br>**F. Was an evaluation of Item Fit performed for each item in the COA? <<YES/NO>>**<br>*(e.g., via INFIT, OUTFIT, CHI-SQUARE-based, Tucker-Lewis Index, item response curve, depending on software used.)*<br><br>***Specify statistic(s) used:***<br><br>***Specify the number of items identified as "mis-fitting" the model:***

**Linearity**<br>Evidence that the COA score is linear throughout its range, such that a 1-point change should be equivalent regardless of the location along the range of the score scale the change occurs. Linearity assumes monotonicity, which is an assumption of the IRT model.<br><br>**Invariant Item Ordering**<br>In a COA, there will be items that are easier and items that are harder to achieve, complete or endorse. Invariant Item Ordering refers to evidence that order of difficulty of the items does not change across different patients or respondents. For example, in the GOS-E, if Invariant Item Ordering is met, then achieving “Independence in the Home” (item 2) should be easier than achieving “Independence outside of the Home” for all assessed by the GOS-E. Also referred to as "item fit", "differential item functioning" (DIF) or "nonintersection of item response curves".<br><br>**Local Independence**<br>Evidence that the responses to any given item in a COA is dependent only on the severity or level of the trait or construct being tested and not on the responses to previous items in the COA. In other words, the only significant source of correlation between any 2 or more items in any COA should be the underlying construct that is	***Specify how these items were handled:***<br><br>**G. Was an assessment of the Monotonicity of the COA performed? <<YES/NO>>**<br>*(e.g., via item-trait interaction chi-square analyses)*<br><br>***Specify statistic(s) used:***<br><br>***Specify statistic(s) results:***<br><br>***Specify the items with disordered thresholds:***<br><br>***If disordered threshold identified, specify remedial action:***<br><br>**H. Was an assessment of the Invariant Item Ordering of the COA performed? <<YES/NO>>**<br>*(e.g., an assessment of systematic differences in responses among respondents (e.g., male vs female; younger vs older), using ANOVA-based approaches)*<br><br>***Specify statistic(s) used:***<br><br>***Specify statistic(s) result:***<br><br>**I. Was an assessment of Local Independence of the COA performed? <<YES/NO>>**<br>*(e.g., via the extraction of a residual correlation matrix)*<br><br>***Specify statistic(s) used:***<br><br>***Specify statistic(s) result:***<br><br>**J. Was an assessment of the Unidimensionality of the COA performed? <<YES/NO>>**<br><br>***Specify statistic(s) used:***<br><br>***Specify statistic(s) result:***

being measured by the COA. Also referred to as "mutual independence".	***pecify the number of factors identified using all of the items of the COA:***<br><br>**K. Was there any other evidence to suggest that there were flaws in the study design, methods or statistical analysis for this QI, rendering the findings unreliable? <<YES/NO>>**<br>***If yes, describe this evidence and where it appears in the text (e.g., page and line number):***<br><br>**L. Summary and Comments (optional):**<br>*(Use this space to summarize the study findings and add comments that may be helpful for reconciling discrepancies and analyzing the evidence downstream.)*

<table>
<tr><th>Quality Indicator (QI)</th><th>QI-Specific MQS Prompts</th></tr>
<tr><td>External Construct Validity: Convergent Validity

The extent to which the COA correlates with another (‘peer’) COA with which it is theoretically expected to correlate. Unlike with criterion validity (see above), here, the comparator is not a ‘gold’ standard but another COA intended to measure the same or similar construct. Evaluation of convergent validity involves consideration and justification of the selected comparator. It is typically measured using the correlation coefficient (e.g. Pearson or Spearman's) or the Area Under the Curve (AUC) via Receiver Operating Characteristics (ROC) analysis, upon dichotomization of the comparator.</td><td>A. Was the reference standard (including its measurement properties) adequately described? <<YES/NO>>

Specify reference standard:

B. Was the reference standard adequately justified or, in your best judgment, can it be considered an appropriate reference standard for the COA? <<YES/NO>>

If no, please specify your reasoning:

C. Were the COA and reference standard administered independently by raters who were blinded/masked to the outcome of the other assessment? <<YES/NO>>

D. Were outcomes for both the COA and criterion standard available in ≥80% of total sample? <<YES/NO>>

E. Were the two administrations conducted within a short period of time? <<YES/NO>>
(i.e., to increase the likelihood that the patient remained stable between measurements)

F. Was there a clear hypothesis about the direction and magnitude of correlation (e.g., ≥0.70) expected between the outcomes of the two assessments? <<YES/NO>>

Specify the hypothesis:

G. Were the statistical approaches used to evaluate this QI clearly reported? <<YES/NO>>
(e.g., refer to description of convergent validity to the left for examples of these approaches.)

H. Were the statistics appropriate to the distribution/type of data produced by the two measures? <<YES/NO/I don’t know>></td></tr>
</table>

(e.g., Pearson, Spearman, Kendall rank correlation, partial correlation for continuous or categorical; ROC-AUC upon dichotomization of the criterion standard. If both convergent and divergent validity were examined together: Multitrait-Multimethod Matrix approach. If you are unsure, seek statistical guidance prior to using the evidence.)

Specify statistic(s) used:

Specify statistic(s) result (including 95% confidence intervals, if available):

I. Was there any other evidence to suggest that there were flaws in the study design, methods or statistical analysis for this QI, rendering the findings unreliable? <<YES/NO>>

If yes, describe this evidence and where it appears in the text (e.g., page and line number):

J. Summary and Comments (optional):

(Use this space to summarize the study findings and add comments that may be helpful for reconciling discrepancies and analyzing the evidence downstream.)

Quality Indicator (QI)	QI-Specific MQS Prompts
**External Construct Validity: Divergent or Discriminant Validity**<br><br>The extent to which the COA **fails** to correlate with a COA that is intended to measure a different construct and therefore is not theoretically expected to correlate. Requires consideration and justification of comparator instrument. Evaluation of divergent validity involves consideration and justification of the selected comparator. It is typically measured using the correlation coefficient (e.g. Pearson or Spearman's) or the Area Under the Curve (AUC) via Receiver Operating Characteristics (ROC) analysis, upon dichotomization of the comparator.	**A. Was the reference standard (including its measurement properties) adequately described? <<YES/NO>>**<br><br>***Specify reference standard:***<br><br>**B. Was the reference standard employed adequately justified or, in your best judgment, reasonable? <<YES/NO>>**<br><br>***If no, please specify your reasoning:***<br><br>**C. Were the COA and reference standard administered independently by raters who were blinded/masked to the outcome of the other assessment? <<YES/NO>>**<br><br>**D. Were outcomes for both the COA and reference standard available in ≥80% of total sample? <<YES/NO>>**<br><br>**E. Were the two administrations conducted within a short period of time? <<YES/NO>>**<br>*(i.e., to increase the likelihood that the patient remained stable between measurements)*<br><br>**F. Was there a clear hypothesis about the direction and magnitude of correlation (e.g., <0.50) expected between the outcomes of the two assessments? <<YES/NO>>**<br><br>***If yes, specify the hypothesis:***<br><br>**G. Were the statistical approaches used to evaluate this QI clearly reported? <<YES/NO>>**<br>*(e.g., refer to description of divergent validity to the left for examples of these approaches.)*<br><br>**H. Were the statistics appropriate to the distribution/type of data produced by the two measures? <<YES/NO/I don't know>>**

	(e.g., Pearson, Spearman, Kendall rank correlation, partial correlation for continuous or categorical; ROC-AUC upon dichotomization of the criterion standard. If both convergent and divergent validity were examined together: Multitrait-Multimethod Matrix approach. If you are unsure, seek statistical guidance prior to using the evidence.)<br><br>***Specify statistic(s) used:***<br><br>***Specify statistic(s) result (including 95% confidence intervals, if available):***<br><br>**I. Was there any other evidence to suggest that there were flaws in the study design, methods or statistical analysis for this QI, rendering the findings unreliable? <<YES/NO>>**<br><br>***If yes, describe this evidence and where it appears in the text (e.g., page and line number):***<br><br>**J. Summary and Comments (optional):**<br>*(Use this space to summarize the study findings and add comments that may be helpful for reconciling discrepancies and analyzing the evidence downstream.)*

Quality Indicator (QI)	QI-Specific MQS Prompts
**Known/Contrasted Groups Validity**<br><br>Supports construct validity by assessing the degree to which the COA can distinguish between groups hypothesized *a priori* to be different (i.e. those who are known to have the particular COI versus those who do not have the particular COI).	**A. Were at least two groups included in the analysis? <<YES/NO>>**<br><br>**B. Were the groups included in the analysis adequately described? <<YES/NO>>**<br><br>**C. Was the selection of these two groups (i.e., one known to exhibit a feature of interest not present in the other) adequately justified, or in your best judgment, appropriate for this comparison? <<YES/NO>>**<br>**D. Was there a clear hypothesis about the expected difference in scores between the two groups? <<YES/NO>>**<br><br>***Specify the hypothesis:***<br><br>**E. Was a threshold for significant difference defined a priori? <<YES/NO>>**<br><br>**F. Were the statistical approaches used to evaluate this QI clearly reported? <<YES/NO>>**<br>*(e.g., refer to description of known/contrasted groups validity to the left for examples of these approaches.)*<br><br>**G. Were the statistics appropriate to the distribution/type of data produced by the two measures? <<YES/NO/I don't know>>**<br>*(If you are unsure, seek statistical guidance prior to using the evidence.)*<br><br>***Specify statistic(s) used:***<br><br>***Specify statistic(s) result (including 95% confidence intervals, if available):***<br><br>**H. Was there any other evidence to suggest that there were flaws in the study design, methods or statistical analysis for this QI, rendering the findings unreliable? <<YES/NO>>**

	If yes, describe this evidence and where it appears in the text (e.g., page and line number):<br><br>**I. Summary and Comments (optional):**<br>*(Use this space to summarize the study findings and add comments that may be helpful for reconciling discrepancies and analyzing the evidence downstream.)*

Quality Indicator (QI)	QI-Specific MQS Prompts
**Ecological Validity**<br><br>The extent to which performance on a COA predicts, transfers or can be functionally related to behavior or performance in 'real-life' settings. The COA is expected to have characteristics similar to a naturally occurring behavior and value in predicting everyday functioning. It is evaluated via two main approaches: veridicality and verisimilitude. *Veridicality* refers to the extent to which the COA correlates with measures of real-world functioning and involves statistically testing the relationship between the COA score and a measure of everyday function (e.g., employment status). *Verisimilitude* refers to the extent to which the task(s) performed or measured as part of the COA resemble those performed in everyday life (e.g., ADLs, attention). It involves developing COAs comprised of everyday tasks or activities.	**A. Was the 'real-world' function, behavior or task adequately described? <<YES/NO>>**<br><br>**B. Was the 'real-word' function, behavior or task adequately justified or, in your best judgment, appropriate for testing the COA? <<YES/NO>>**<br><br>***If no, please specify your reasoning:***<br><br>**C. Was a recognized approach for establishing Ecological Validity employed? <<YES/NO/I don't know>>**<br>*(e.g., see the definition to the left for descriptions of the two main approaches: veridicality and verisimilitude; Face Validity is not an appropriate approach for establishing ecological validity. If you are unsure, seek relevant guidance prior to using the evidence.)*<br><br>***If yes, please specify or describe approach used:***<br><br>**D. If veridicality was used, were the COA and measure of everyday function administered independently by raters who were blinded/masked to the outcome of the other assessment? <<YES/NO/NA>>**<br>*(Select NA if veridicality was not used.)*<br><br>**E. If veridicality was used, were measurements for both the COA and measure of function available in ≥80% of total sample? <<YES/NO/NA>>**<br>*(Select NA if veridicality was not used.)*<br><br>**F. Was there a clear hypothesis about the expected relationship between the COA and 'real-word' function? <<YES/NO>>**<br>*(e.g., for veridicality, was the direction and magnitude of the correlation pre-specified? for verisimilitude, were the everyday tasks that the COA items were meant to resemble pre-specified?)*

G. Were the qualitative and/or statistical approaches used to evaluate ecological validity clearly reported? <<YES/NO>>

H. Were the qualitative approach and/or statistics appropriate to the distribution/type of data produced by the COA and 'real-word' function, behavior or task? <<YES/NO/I don't know>>

(If you are unsure, seek statistical guidance prior to using the evidence.)

Specify approach and/or statistic(s) used:

Specify qualitative and/or statistic(s) result (including 95% confidence intervals, if available):

I. Was there any other evidence to suggest that there were flaws in the study design, methods or statistical analysis for this QI, rendering the findings unreliable? <<YES/NO>>

If yes, describe this evidence and where it appears in the text (e.g., page and line number):

J. Summary and Comments (optional):

(Use this space to summarize the study findings and add comments that may be helpful for reconciling discrepancies and analyzing the evidence downstream.)

Quality Indicator (QI)	QI-Specific MQS Prompts
**Cross-Cultural Validity**<br><br>COSMIN defines this as the "degree to which the performance of the items on a translated or culturally adapted COA are an adequate reflection of the performance of the items of the original version of the COA instrument". It is typically calculated by confirmatory factor analysis (Classical Test Theory) or invariant item ordering/differential item function (DIF) between language groups (Item Response Theory – see internal construct validity).<br><br>*NB: While this form assesses the development of the translated or culturally-adapted version relative to the original version, if the translated version will be used in the COU, then it must also undergo formal validation via the EB-COP.*	**A. Were the source/original and target languages and/or cultures adequately described? <<YES/NO>>**<br><br>***Specify source language/culture:***<br><br>***Specify target language/culture:***<br><br>**B. Was the original COA forward translated by two independent, bilingual translators whose mother tongue was that of the target language? <<YES/NO>>**<br>*(Here, to qualify as 'independent', the translators should have been blinded/masked to the other's translation.)*<br><br>**C. Were the discrepancies between the two independent forward translations and their resolution adequately described? <<YES/NO>>**<br><br>**D. Was the synthesized forward translation backtranslated by two independent, bilingual translators (whose mother tongue is the source language)? <<YES/NO>>**<br>*(Here, to qualify as 'independent', the translators should have been blinded/masked to the other's translation and to the original version of the COA.)*<br><br>**E. Did an independent third party review the final translation and establish/finalize its cross-cultural equivalence? <<YES/NO>>**<br><br>**F. Was the final translated version pre-tested (i.e., subjected to cognitive testing) to check interpretation, cultural relevance and ease of comprehension? <<YES/NO>>**<br><br>**G. Was the sample used in the pre-test adequately described and representative of the target population? <<YES/NO>>**<br><br>**H. Were the statistical approaches used to evaluate this QI clearly reported? <<YES/NO>>**

(e.g., for CTT, confirmatory factor analysis; for IRT, invariant item ordering to identify systematic differences between language groups.)

I. Were the statistics appropriate to the type of data produced by the COA? <<YES/NO>>

(If you are unsure, seek statistical guidance prior to using the evidence.)

Specify statistic(s) used:

Specify statistic(s) result:

If no, specify why:

J. Was there any other evidence to suggest that there were flaws in the study design, methods or statistical analysis for this QI, rendering the findings unreliable? <<YES/NO>>

If yes, describe this evidence and where it appears in the text (e.g., page and line number):

K. Summary and Comments (optional):

(Use this space to summarize the study findings and add comments that may be helpful for reconciling discrepancies and analyzing the evidence downstream.)

Quality Indicator (QI)	QI-Specific MQS Prompts
**Diagnostic Validity/Ability/Accuracy (*includes Diagnostic Cut-Off Score/Threshold*)**<br><br>The extent to which a COA can distinguish between groups of patients/participants (e.g. TBI versus non-TBI or mild versus moderate versus severe) that have been identified by an independent, field-accepted 'gold' or other standard approach. Standard methods for assessing diagnostic ability include sensitivity/specificity calculations and Area Under the Curve (AUC) via Receiver Operating Characteristics (ROC) analysis. May also be referred to as "discriminative ability" and may result in the identification of a Diagnostic Cut-off score to aid in the interpretation of the COA. For example, a total score of 8 on the Coma Recovery Scale-Revised identifies individuals with the diagnostic criteria for conscious awareness with 93% sensitivity and 96% specificity. For PoU1 (accurately diagnose TBI), Diagnostic and Criterion Validity are equivalent.	**A. Was the 'gold' or criterion standard adequately described? <<YES/NO>>**<br><br>***Specify 'gold'/criterion standard:***<br><br>**B. Was the criterion standard employed adequately justified or, in your best judgment, reasonable for diagnosing TBI? <<YES/NO>>**<br><br>***If no, please specify your reasoning:***<br><br>**C. Was there less than 30% item overlap between the COA and its 'gold' standard? <<YES/NO>>**<br><br>**D. Were the outcomes of the COA and criterion standard determined independently by raters who were blinded/masked to the outcome of the other assessment? <<YES/NO>>**<br>*(That is, was the individual using the COA blinded to the outcome of the gold standard, and vice versa?)*<br><br>**E. Were outcomes for both the COA and criterion standard available in ≥80% of total sample? <<YES/NO>>**<br><br>**F. Were the two administrations conducted within a short period of time? <<YES/NO>>**<br>*(i.e., to increase the likelihood that the patient remained stable between measurements)*<br><br>**G. Was there a clear hypothesis about the degree of diagnostic accuracy (e.g., sensitivity/specificity) expected to be achieve by the COA in relation to the 'gold' standard? <<YES/NO>>**<br><br>***If yes, specify the hypothesis:***

H. Were the statistical approaches used to evaluate this QI clearly reported? <<YES/NO>>

(e.g., refer to description of diagnostic validity to the left for examples of these approaches.)

I. Were the statistics appropriate to the distribution/type of data produced by the two assessments? <<YES/NO/I don't know>>

(e.g., sensitivity/specificity, Area Under the Curve (AUC) c-statistic via Receiver Operating Characteristics (ROC) analysis, Youden's Index. If you are unsure, seek statistical guidance prior to using the evidence.)

Specify statistic(s) used:

Specify statistic(s) result (including 95% confidence intervals, if available):

Specify Diagnostic cut-off score/threshold and supporting statistics (if determined):
(e.g., COA score at which the maximum sensitivity/specificity were reported.)

J. Was there any other evidence to suggest that there were flaws in the study design, methods or statistical analysis for this QI, rendering the findings unreliable? <<YES/NO>>

If yes, describe this evidence and where it appears in the text (e.g., page and line number):

K. Summary and Comments (optional):

(Use this space to summarize the study findings and add comments that may be helpful for reconciling discrepancies and analyzing the evidence downstream.)

Quality Indicator (QI)	QI-Specific MQS Prompts
**Prognostic Validity/Ability/Accuracy (*includes Prognostic Cut-Off Score/Threshold*)**<br><br>The extent to which a COA can predict a future (health-related) event or outcome of functional or global relevance, such as mortality, independence in activities of daily living (ADLs) or return to driving. Standard methods for assessing prognostic ability include sensitivity/specificity calculations, Area Under the Curve (AUC) via Receiver Operating Characteristics (ROC) analysis and Positive and Negative Predictive Values (PPV and NPV, respectively). May also be referred to as "predictive ability" and it may be used interchangeably with predictive validity (described previously) depending on the future outcome. It may also result in the identification of a Prognostic Cut-off score to aid in the interpretation of the COA. For example, a score of >13.5 seconds on the Timed Up and GO performance-based test has been shown to identify community-dwelling older adults at high risk of falling with 80% sensitivity and 100% specificity.	**A. Was the future outcome adequately described? <<YES/NO>>**<br>*(This should include the time in the future at which the outcome was determined.)*<br><br>***Specify future outcome, including time-frame:***<br><br>**B. Was the appropriateness of the future outcome for this population adequately justified or, in your best judgment, reasonable for this population? <<YES/NO>>**<br><br>***If no, please specify your reasoning:***<br><br>**C. Was there less than 70% item overlap between the COA and the future outcome? <<YES/NO>>**<br><br>**D. Were the COA and future outcome administered independently by raters who were blinded/masked to the outcome of the other assessment? <<YES/NO>>**<br>*(That is, was the individual obtaining the future outcome blind to the outcome of the COA?)*<br><br>**E. Were measurements of both the COA and future outcome available in ≥80% of total sample? <<YES/NO>>**<br><br>**F. Was there a clear hypothesis about the degree of prognostic accuracy (e.g., positive predictive value) expected to be achieve by the COA in relation to the future outcome? <<YES/NO>>**<br><br>***If yes, specify the hypothesis:***<br><br>**G. Were the statistical approaches used to evaluate this QI clearly reported? <<YES/NO>>**<br>*(e.g., refer to description of prognostic validity to the left for examples of these approaches.)*<br><br>**H. Were the statistics appropriate to the distribution/type of data produced by the two assessments? <<YES/NO/I don't know>>**

	(e.g., sensitivity/specificity, Area Under the Curve (AUC) c-statistic via Receiver Operating Characteristics (ROC) analysis, Positive and Negative Predictive Value. If you are unsure, seek statistical guidance prior to using the evidence.)<br><br>***Specify statistic(s) result (including 95% confidence intervals, if available):***<br><br>***Specify Prognostic cut-off score/threshold and supporting statistics (if determined):***<br>*(e.g., COA score at which the maximum sensitivity/specificity were reported.)*<br><br>***If no, specify why:***<br><br>**I. Was there any other evidence to suggest that there were flaws in the study design, methods or statistical analysis for this QI, rendering the findings unreliable? <<YES/NO>>**<br><br>***If yes, describe this evidence and where it appears in the text (e.g., page and line number):***<br><br>**J. Summary and Comments (optional):**<br>*(Use this space to summarize the study findings and add comments that may be helpful for reconciling discrepancies and analyzing the evidence downstream.)*

RESPONSIVENESS

Responsiveness refers to the ability of the COA to detect true change (i.e. change that *has* occurred) over time. It is an essential property of evaluative COAs. The assessment of responsiveness requires *a priori* specification of expected magnitude and/or direction of effect or comparison to ‘known’ groups. Internal and external responsiveness (described below) are the two ways in which it can be assessed.

Quality Indicator (QI)	QI-Specific MQS Prompts
**Internal Responsiveness**<br><br>The extent to which a COA score changes over a pre-specified time frame due to treatment effects or natural history change. It is usually measured within the context of the study, e.g. repeated measures design of a treatment/intervention previously shown to be efficacious or well-established natural history changes. It is involves statistics that are based on the distribution of the data generated by the study and can therefore be strongly influence by the study design (e.g., sample size). Common measures of internal responsiveness include Cohen's effect size, the standardized response mean and the paired t-test.	**A. Was the COA employed in a longitudinal design with at least two independent measurements? <<YES/NO>>**<br><br>**B. Was the time-frame between the two measurements (e.g., before and after the intervention) specified? <<YES/NO>>**<br><br>***Specify time-frame:***<br><br>**C. Were the two measurements of the COA available in ≥80% of total sample? <<YES/NO>>**<br><br>**D. If an intervention or other event occurred between measurements, was it adequately described? <<YES/NO>>**<br><br>***If yes, briefly describe the intervention/event***:<br><br>**E. Was sufficient prior evidence for the effectiveness of the intervention or anticipated natural history change during the pre-specified time-frame presented? <<YES/NO>>**<br><br>***If yes, describe the evidence***:<br><br>**F. Was there any evidence to suggest that a proportion of the sample showed change (improvement or deterioration) in the intended construct during the study interval (i.e., some indication that the sample was not stable during that time) presented? <<YES/NO>>**

G. Was there a clear hypothesis about the magnitude and direction of the change (e.g., effect size) expected to be observed? <<YES/NO>>

H. Were the statistical approaches used to evaluate this QI clearly reported? <<YES/NO>>

(e.g., refer to description of internal responsiveness to the left for examples of these approaches.)

I. Were the statistics appropriate to the type of data produced by the COA? <<YES/NO/I don't know>>

(e.g., Cohen's effect size, the standardized response mean and the paired t-test. If you are unsure, seek statistical guidance prior to using the evidence.)

Specify statistic(s) used:

Specify statistic(s) result:

If no, specify why:

J. Was there any other evidence to suggest that there were flaws in the study design, methods or statistical analysis for this QI, rendering the findings unreliable? <<YES/NO>>

If yes, describe this evidence and where it appears in the text (e.g., page and line number):

K. Summary and Comments (optional):

(Use this space to summarize the study findings and add comments that may be helpful for reconciling discrepancies and analyzing the evidence downstream.)

Quality Indicator (QI)	QI-Specific MQS Prompts
**Minimal (Statistically) Important Difference (MID)**<br><br>A statistical or distribution-based estimate of the smallest change in an individual's COA score that needs to be achieved or observed to ensure that it is beyond measurement error. Several approaches to calculating the MID exist, including the minimum detectable change (MDC), which is derived from the standard error of measurement (SEM), the Bland-Altman Plot Limits of Agreement (LoA) and the reliable change index (RCI). The SEM is an absolute measure of reliability and estimates the expected variation in observed scores due to measurement error. MIDs are typically determined in the context of reliability or responsiveness studies.	**A. Has the MID been determined in the context of a study evaluating the reliability and/or internal responsiveness of the COA or was the COA employed in a longitudinal design with at least two independent measurements? <<YES/NO>>**<br><br>**B. For COAs employed in a longitudinal design, was the time-frame or interval between the two measurements specified? <<YES/NO>>**<br><br>***Specify time-frame/interval:***<br><br>**C. For COAs employed in a longitudinal design, were the two measurements of the COA available in ≥80% of total sample? <<YES/NO>>**<br><br>**D. For COAs employed in a longitudinal design, if an intervention or other event occurred between measurements, was it adequately described? <<YES/NO>>**<br><br>***If yes, briefly describe the intervention/event*:**<br><br>**E. Were the statistical approaches used to determine this QI clearly reported? <<YES/NO>>**<br>*(e.g., refer to description of MID to the left for examples of these approaches.)*<br><br>**F. Were the statistics appropriate to the distribution/type of data produced by the two assessments? <<YES/NO/I don't know>>**<br>*(If you are unsure, seek statistical guidance prior to using the evidence.)*<br><br>***Specify statistic(s) used:***<br><br>***Specify MID identified:***<br><br>***If no, specify why:***<br><br>**G. Was there any other evidence to suggest that there were flaws in the study design, methods or statistical analysis for this QI, rendering the findings unreliable? <<YES/NO>>**

	If yes, describe this evidence and where it appears in the text (e.g., page and line number):<br><br>**H. Summary and Comments (optional):**<br>*(Use this space to summarize the study findings and add comments that may be helpful for reconciling discrepancies and analyzing the evidence downstream.)*

Quality Indicator (QI)	QI-Specific MQS Prompts
**External Responsiveness**<br><br>The extent to which changes in a COA over a specified time frame relate to corresponding changes in an independent, external measure ('gold' or 'peer') of the same or similar construct or another relevant measure of health status. It is typically evaluated by calculating the correlation between the change in the COA and the change in the reference standard (a.k.a. ‘correlational approach’) or by calculating Area Under the Curve (AUC) via Receiver Operating Characteristics (ROC) analysis in a dichotomized (i.e. changed versus not changed) or binomial reference standard (e.g. a patient's self-reported transition score - improved vs. not improved - following an intervention). It is considered the more rigorous of the two types of responsiveness because it compares to external measures of change, and may form the basis for the identification of the minimum clinically important difference (MCID, described below). External Responsiveness is also known as “longitudinal construct validity”.	**A. Was the independent external measure or reference for change adequately described? <<YES/NO>>**<br><br>***Specify the independent ‘gold’ or reference measure:***<br><br>**B. Were the COA and external measure employed in a longitudinal design with at least two independent measurements for each? <<YES/NO>>**<br><br>**C. Was the time-frame between the two measurements of the COA and external measure (e.g., before and after the intervention) specified? <<YES/NO>>**<br><br>***Specify time-frame:***<br><br>**D. Were the two administrations of the COA and the external reference conducted within the same pre-specified time-frame to ensure comparability of the change detected by each instrument? <<YES/NO>>**<br><br>**E. Were the two measurements of the COA available in ≥80% of total sample? <<YES/NO>>**<br><br>**F. Were the two measurements of the external measure available in ≥80% of total sample? <<YES/NO>>**<br><br>**G. Was there ≥80% overlap in the samples that underwent both administrations of the COA, and in the samples that underwent both administrations of the external measure? <<YES/NO>>**<br><br>**H. If an intervention or other event occurred between measurements, was it adequately described? <<YES/NO>>**

I. Was there any evidence to suggest that a proportion of the sample showed change (improvement or deterioration) in the intended construct during that time (i.e., some indication that the sample was not stable during that time) presented? <<YES/NO>>

J. Was there a clear hypothesis about the magnitude of the correlation or agreement between the change in the COA and the change in the external measure? <<YES/NO>>

K. Were the statistical approaches used to evaluate this QI clearly reported? <<YES/NO>>

(e.g., refer to description of external responsiveness above for examples of these approaches.)

L. Were the statistics appropriate to the distribution/type of data produced by the two assessments? <<YES/NO/I don't know>>

(If you are unsure, seek statistical guidance prior to using the evidence.)

Specify statistic(s) used:

Specify statistic(s) result:

M. Was there any other evidence to suggest that there were flaws in the study design, methods or statistical analysis for this QI, rendering the findings unreliable? <<YES/NO>>

If yes, describe this evidence and where it appears in the text (e.g., page and line number):

N. Summary and Comments (optional):

(Use this space to summarize the study findings and add comments that may be helpful for reconciling discrepancies and analyzing the evidence downstream.)

Quality Indicator (QI)	QI-Specific MQS Prompts
**Minimum Clinically Important Difference (MCID)**<br><br>An anchor-based estimate of the smallest change in an individual's COA score that has been determined to be meaningful to the patient and/or clinician. Typically determined by comparing the change in the COA score to a question or survey (i.e. an anchor) regarding the patients perceived health/status at a subsequent time point relative to baseline. May be determined in the context of an external responsiveness study.	**A. Was the independent external measure or reference for change adequately described? <<YES/NO>>**<br><br>**B. Were the COA and external measure both administered at least twice by independent examiners? <<YES/NO>>**<br><br>**C. Was the time-frame between the two administrations of the COA and external measure (e.g., before and after the intervention) specified? <<YES/NO>>**<br><br>***Specify time-frame:***<br><br>**D. Were the two administrations of the COA and the external reference conducted within the same pre-specified time-frame to ensure comparability of the change detected by each instrument? <<YES/NO>>**<br><br>**E. Were the two administrations of the COA available in ≥80% of total sample? <<YES/NO>>**<br><br>**F. Were the two administrations of the external measure available in ≥80% of total sample? <<YES/NO>>**<br><br>**G. Was there ≥80% overlap in the samples that underwent both administrations of the COA, and in the samples that underwent both administrations of the external measure? <<YES/NO>>**<br><br>**H. If an intervention or other event occurred between administrations, was it adequately described? <<YES/NO>>**<br><br>**I. Was the direction (i.e., improvement vs deterioration) of the MCID clearly indicated? <<YES/NO>**

	**J. Were the statistical approaches used to determine the MCID clearly reported? <<YES/NO>>**<br>*(e.g., refer to description of the MCID above for examples of these approaches.)*<br><br>**K. Were the statistics appropriate to the distribution/type of data produced by the two assessments? <<YES/NO/I don't know>>**<br>*(If you are unsure, seek statistical guidance prior to using the evidence.)*<br><br>***Specify statistic(s) used:***<br><br>***Specify MCID:***<br><br>**L. Was there any other evidence to suggest that there were flaws in the study design, methods or statistical analysis for this QI, rendering the findings unreliable? <<YES/NO>>**<br><br>***If yes, describe this evidence and where it appears in the text (e.g., page and line number):***<br><br>**M. Summary and Comments (optional):**<br>*(Use this space to summarize the study findings and add comments that may be helpful for reconciling discrepancies and analyzing the evidence downstream.)*

INTERPRETABILITY

The remaining QIs represent properties of a COA that may lend to its clinical utility and facilitate the interpretation of an individual's score.

Quality Indicator (QI)	QI-Specific MQS Prompts
**Normative Values (derived from a relevant sample/population)**<br><br>The distribution of COA scores derived from a 'healthy' or relevant demographically-related (with respect to age, sex, education and race) reference population that represent "normal" values and that may serve as a rough guideline for interpreting COA scores derived from the Population of interest.	**A. Were the characteristics of the sample/population from which the normative values were obtained adequately described? <<YES/NO>>**<br><br>**B. Was the sample/population that produced the normative values demographically-similar to the Population of interest? <<YES/NO>>**<br><br>**C. Was the adequacy of the psychometric properties (e.g., reliability and validity) of the COA in the normative sample/population addressed? <<YES/NO>>**<br><br>**D. Were the statistical approaches used to determine the normative values clearly reported? <<YES/NO>>**<br><br>**E. Were the statistics appropriate to the type of data produced by the COA? <<YES/NO/I don't know>>**<br>*(e.g, mean and standard deviation for normally distributed interval data; median and quartiles for non-normally distributed interval and nominal/ordinal data; sensitivity/specificity of a cut score. If you are unsure, seek statistical guidance prior to using the evidence.)*<br><br>***Specify statistic(s) used:***<br><br>***Specify statistic(s) result:***<br><br>***Specify the recommended threshold or cut-off for 'deviation' from the norm (include evidence of specificity/sensitivity of this cut-off, if appropriate):***<br><br>***If no, specify why:***

	**F. Was there any other evidence to suggest that there were flaws in the study design, methods or statistical analysis for this QI, rendering the findings unreliable? <<YES/NO>>**<br><br>***If yes, describe this evidence and where it appears in the text (e.g., page and line number):***<br><br>**G. Summary and Comments (optional):**<br>*(Use this space to summarize the study findings and add comments that may be helpful for reconciling discrepancies and analyzing the evidence downstream.)*

Quality Indicator (QI)	QI-Specific MQS Prompts
**Score Variability and Floor and Ceiling Effects**<br><br>Assessment of the distribution of COA scores in the Population of interest to ensure variation in the scores across the full range of the COA and that a large proportion of the patients did not achieve the same score, particularly at the extremes - i.e., the lowest (floor) or highest (ceiling) scores. In IRT, this is referred to as item difficulty or targeting and involves calculating the Item Difficulty Parameter or displaying Person-Item Threshold maps.	**A. Was the distribution of COA scores adequately reported? <<YES/NO/NA>>**<br>*(e.g., for ordinal outcomes, the frequency of individuals who achieved each level of outcome; for interval outcomes, summary statistics that include mean/median, spread, range; for IRT-based approaches, determination of Item Difficultly Parameter or display of Person-Item Threshold Map)*<br><br>***Describe distribution of COA scores:***<br><br>**B. Was the possibility/presence of floor effects addressed? <<YES/NO/NA>>**<br>*(Select NA if floor effects are not a concern in the study sample for the COA.)*<br><br>***Specify % of sample with lowest score on the COA (CTT) or persons' mean location score, if greater than 0 (IRT):***<br><br>**C. Was the possibility/presence of ceiling effects addressed? <<**YES/NO/NA>><br>*(Select NA if ceiling effects are not a concern in the study sample for the COA.)*<br><br>**D. Was there any other evidence to suggest that there were flaws in the study design, methods or statistical analysis for this QI, rendering the findings unreliable? <<YES/NO>>**<br><br>***If yes, describe this evidence and where it appears in the text (e.g., page and line number):***<br><br>**E. Summary and Comments (optional):**<br>*(Use this space to summarize the study findings and add comments that may be helpful for reconciling discrepancies and analyzing the evidence downstream.)*

***Step V*: Analyze the COA Evidence**

What is the evidence in support of the COA in/for the intended Context of Use?

In Step V, data collected for each PoU-specific QI from all included articles reviewed in Steps IV-B–D is exported from Qualtrics into an Excel-based Evidence Summary Table. For each study assessing the QI, evaluate whether cut-offs shown below are met ("adequate"), not met ("inadequate"), or inconclusive.

Quality Indicators for Grading and Development of Recommendations for COAs	QI Cut-Offs
Internal Consistency	Correlation coefficient ≥ 0.70
Test-Retest Reliability (cross-sectional)	Correlation coefficient ≥ 0.70
Test-Retest Reliability (longitudinal)	Correlation coefficient ≥ 0.70
Inter-Rater Reliability (cross-sectional)	Correlation coefficient ≥ 0.70
Inter-Rater Reliability (longitudinal)	Correlation coefficient ≥ 0.70
Intra-Rater Reliability (cross-sectional)	Correlation coefficient ≥ 0.70
Intra-Rater Reliability (longitudinal)	Correlation coefficient ≥ 0.70
Alternate/Parallel-Forms Reliability (cross-sectional)	Correlation coefficient ≥ 0.70
Alternate/Parallel-Forms Reliability (longitudinal)	Correlation coefficient ≥ 0.70
Concurrent Validity	Correlation coefficient ≥ 0.60<br>ROC AUC ≥ 0.70
Predictive Validity	Correlation coefficient ≥ 0.60<br>ROC AUC ≥ 0.70
Internal Construct Validity (Classical Approaches) – Unidimensionality	Depends on the Model/Method
Internal Construct Validity (IRT Approach) (Unidimensionality, Monotonicity/Scalability/Linearity, Invariant Item Ordering, and Local Independence)	Depends on the Model/Method
Convergent Validity	Correlation coefficient ≥ 0.60<br>ROC AUC ≥ 0.70
Divergent or Discriminant Validity	Correlation coefficient < 0.30<br>ROC AUC < 0.70
Known/Contrasted Groups Validity	Cohen's d ≥ 0.50 with P ≤ 0.05
Ecologic Validity	*Veridicality* – Correlation ≥ 0.70<br>*Verisimilitude* – ≥70%* of items resemble tasks/activities performed in everyday life.
Cross-Cultural Validity	Process is appropriate.
Diagnostic Validity/Accuracy	Sensitivity >80%, Specificity >60%<br>ROC AUC ≥ 0.80<br>LR+ >10<br>LR- <0.1
Diagnostic Cut-off Score	Described, with adequate diagnostic validity
Prognostic Validity/Accuracy	Sensitivity >80%, Specificity >60%<br>ROC AUC ≥ 0.80<br>LR+ >10<br>LR- <0.1
Prognostic Cut-off Score	Described, with adequate prognostic validity

Internal Responsiveness	Direction and magnitude of change as stated in *a priori* hypothesis
Minimal (Statistically) Important Difference (MID)	Described
External Responsiveness	Correlation coefficient ≥ 0.70<br>ROC AUC ≥ 0.70
Minimum Clinically Important Difference (MCID)	Described
Normative Values (reference values from a relevant population)	Described
Score Variability and Floor and Ceiling Effects	All scores <15% of sample

***Step VI*: Synthesize the Evidence and Develop a Recommendation for the COA**

(What is the strength of the evidence supporting the COA in the intended Context of Use, and what are the implications for its research and clinical application?)

At the start of Step VI, you will be asked to provide your email, to re-enter or paste the COA of interest and P-P-C-C question, select the PoU from a drop-down menu. The form will populate with the appropriate QIs to be graded based on the chosen PoU. In step VI, are asked to synthesize the Evidence Summary Table from the previous step in order to rate each QI as 'adequate' (i.e., meeting the threshold), 'inadequate' (i.e., not meeting the threshold), or 'not determined' (i.e., not been studied in a relevant, high-quality study). It is possible multiple articles in your review have assessed the same QI. In the event of conflicting findings, judge the methodological qualities of the studies when scoring. If findings are equivocal, and the strength of the study design is equivalent (e.g., both Class II), rate the outcome as "not determined." You may provide optional comments in the online form. Your responses for mandatory QIs will determine the numbers needed in order to produce a grade and recommendation, based on the criteria below. This automated result will be displayed, and the information will also be sent to the email address provided.

- **Grade I - Recommended without Reservation:** For specified COU, *all* QIs found to be "adequate".
- **Grade II – Recommended with Reservation(s):** For specified COU, *all* QIs found to be "adequate", except for no more than 2 QIs found to be "undetermined". No QIs are "inadequate". Focused research recommendations outlined.
- **Grade III – Not Currently Recommended:** For specified COU, all QIs found to be "adequate", except >2 are "undetermined". No QIs are "inadequate". Focused research recommendations outlined.
- **Grade IV – Recommended Against ("Fatal Flaw"):** For specified COU, ≥1 QI found to be "inadequate".

This concludes the final step of the EB-COP.

Acknowledgments

The development of the EB-COP was funded by the DoD-funded TED Initiative Seed Grant (W81XWH-14-2-0176) and supported by the American Academy of Neurology (AAN), Spaulding Rehabilitation Hospital, the Medical College of Wisconsin, the University of Florida, the Critical Path Institute and the Mayo Clinic.

References

1. U.S. Food and Drug Administration. Drug Development Tools Qualification Programs - Roadmap to Patient-Focused Outcome Measurement in Clinical Trials (text version) 2016. U.S. Department of Health and Human Services. Available at: http://www.fda.gov/Drugs/DevelopmentApprovalProcess/DrugDevelopmentToolsQualificationProgram/ucm370177.htm [Accessed 11th May 2016]
2. American Academy of Neurology (AAN). 2011. Clinical Practice Guideline Process Manual, 2011 Ed. St. Paul, MN: The American Academy of Neurology.
3. Mokkink et al., 2010. The COSMIN checklist for assessing the methodological quality of studies on measurement properties of health status measurement instruments: an international Delphi study. Qual Life Res, 19:539–549.
