# Supplementary material for: An evidence-based methodology for systematic evaluation of clinical outcome assessment measures for traumatic brain injury
Source: PLoS One. 2020 Dec 14;15(12):e0242811. doi: 10.1371/journal.pone.0242811 (PMC7735614; doi:10.1371/journal.pone.0242811)
Supplement: S3 File — The EB-COP search filter extends the filter recommended by COSMIN by adding terms that help identify studies focusing on TBI outcome measures. (DOCX) [file pone.0242811.s003.docx]

**S3. Search term filter to promote capture of studies on TBI outcome measures.**

MEDLINE

1 (instrumentation or methods).sh. 233243 Advanced

2 (Validation studies or comparative study).pt. 1834787 Advanced

3 exp psychometrics/ 62204 Advanced

4 psychometr*.ti,ab,kw. 35158 Advanced

5 (clinimetr* or clinometr*).ti,ab,kw. 778 Advanced

6 exp "outcome assessment (health care)"/ 848289 Advanced

7 outcome assessment*.ti,ab,kw. 4642 Advanced

8 outcome measure*.ti,ab,kw. 180602 Advanced

9 exp observer variation/ 36442 Advanced

10 (observer adj2 variation*).ti,ab,kw. 1109 Advanced

11 exp health status indicators/ 232116 Advanced

12 exp "reproducibility of results"/ 327811 Advanced

13 reproducib*.ti,ab,kw. 132751 Advanced

14 exp discriminant analysis/ or (discriminant* adj2 analys*).mp. [mp=title, abstract, original title, name of substance word, subject heading word, keyword heading word, protocol supplementary concept word, rare disease supplementary concept word, unique identifier] 21499 Advanced

15 (reliab* or unreliab* or valid* or coefficient or homogeneity or homogeneous or "internal* consisten*").ti,ab,kw. 1075331 Advanced

16 (cronbach* and (alpha or alphas)).ti,ab,kw. 15373 Advanced

17 (item and (correlat* or selection* or reduction*)).ti,ab,kw. 20567 Advanced

18 (agreement or precision or imprecision or "precise values" or test-retest).ti,ab,kw. 328814 Advanced

19 (test and retest).ti,ab,kw. 20615 Advanced

20 reliab*.mp. and (test* or retest*).ti,ab,kw. [mp=title, abstract, original title, name of substance word, subject heading word, keyword heading word, protocol supplementary concept word, rare disease supplementary concept word, unique identifier] 114212 Advanced

21 (stability or interrater or inter-rater or intrarater or intra-rater or intertester or inter-tester or intratester or intra-tester or interobserver or inter-observer or intraobserver or intra-observer or intertechnician or inter-technician or intratechnician or intra-technician or interexaminer or inter-examiner or intraexaminer or intra-examiner or interassay or inter-assay or intraassay or intra-assay or interindividual or inter-individual or intraindividual or intra-individual or interparticipant or inter-participant or intraparticipant or intra-participant or kappa*2 or "k" or repeatab*).ti,ab,kw. 878392 Advanced

22 ((replicab* or repeated) and (measure* or findings or result or results or test or tests)).ti,ab,kw. 170357 Advanced

23 (generaliza* or generalisa* or concondance).ti,ab,kw. 33359 Advanced

24 (intraclass and correlation*).ti,ab,kw. 17274 Advanced

25 (discriminative or "known group*" or factor analysis or factor analyses or dimension* or subscale*).ti,ab,kw. 519498 Advanced

26 (multitrait and scaling and (analysis or analyses)).ti,ab,kw. 134 Advanced

27 (item discriminant or interscale correlation* or error or errors or "individual variability").ti,ab,kw. 240787 Advanced

28 (variability and (analysis or values)).ti,ab,kw. 77502 Advanced

29 (uncertainty and (measure* or measuring)).ti,ab,kw. 15363 Advanced

30 ("standard error of measurement" or sensitiv* or responsive*).ti,ab,kw. 1317288 Advanced

31 ((minimal* or clinical*) and (important or significant or detectable) and (change or difference)).ti,ab,kw. 181396 Advanced

32 (small* and (real or detectable) and (change or difference)).ti,ab,kw. 5739 Advanced

33 (meaningful change or ((ceiling or floor) adj3 effect*) or "item response model" or irt or rasch or "differential item functioning" or dif or "computer adaptive testing" or "item bank" or "cross-cultural*").ti,ab,kw. 20992 Advanced

34 or/1-33 6481000 Advanced

35 "glasgow outcome".mp. or gos.tw. or gose.tw. [mp=title, abstract, original title, name of substance word, subject heading word, keyword heading word, protocol supplementary concept word, rare disease supplementary concept word, unique identifier] 5284 Advanced

36 34 and 35 3203 Advanced

37 (tbi or mtbi or "traumatic brain").mp. or exp head injuries/ or exp craniocerebral trauma/ or concuss*.mp. [mp=title, abstract, original title, name of substance word, subject heading word, keyword heading word, protocol supplementary concept word, rare disease supplementary concept word, unique identifier] 151486 Advanced

38 ((head or cranial or skull) adj2 (trauma* or injur* or fractur*)).mp. [mp=title, abstract, original title, name of substance word, subject heading word, keyword heading word, protocol supplementary concept word, rare disease supplementary concept word, unique identifier] 44384 Advanced

39 36 and (37 or 38) 1434 Advanced

EMBASE

1 (instrumentation or methods).sh. 150194 Advanced

2 exp intermethod comparison/ or exp data collection method/ or exp validation study/ or exp feasibility study/ or exp pilot study/ or exp psychometry/ or exp reproducibility/ or exp observer variation/ or exp discriminant analysis/ or exp validity/ 1526246 Advanced

3 exp psychometrics/ 71968 Advanced

4 psychometr*.ti,ab,kw. 40448 Advanced

5 (clinimetr* or clinometr*).ti,ab,kw. 1159 Advanced

6 exp "outcome assessment (health care)"/ 359033 Advanced

7 outcome assessment*.ti,ab,kw. 8691 Advanced

8 outcome measure*.ti,ab,kw. 217827 Advanced

9 exp observer variation/ 17901 Advanced

10 (observer adj2 variation*).ti,ab,kw. 1640 Advanced

11 exp health status indicators/ 14453 Advanced

12 exp "reproducibility of results"/ 177437 Advanced

13 reproducib*.ti,ab,kw. 145673 Advanced

14 exp discriminant analysis/ or (discriminant* adj2 analys*).mp. [mp=title, abstract, heading word, drug trade name, original title, device manufacturer, drug manufacturer, device trade name, keyword, floating subheading] 22800 Advanced

15 (reliab* or unreliab* or valid* or coefficient or homogeneity or homogeneous or "internal* consisten*").ti,ab,kw. 1247181 Advanced

16 (cronbach* and (alpha or alphas)).ti,ab,kw. 18150 Advanced

17 (item and (correlat* or selection* or reduction*)).ti,ab,kw. 26193 Advanced

18 (agreement or precision or imprecision or "precise values" or test-retest).ti,ab,kw. 340302 Advanced

19 (test and retest).ti,ab,kw. 23292 Advanced

20 reliab*.mp. and (test* or retest*).ti,ab,kw. [mp=title, abstract, heading word, drug trade name, original title, device manufacturer, drug manufacturer, device trade name, keyword, floating subheading] 139580 Advanced

21 (stability or interrater or inter-rater or intrarater or intra-rater or intertester or inter-tester or intratester or intra-tester or interobserver or inter-observer or intraobserver or intra-observer or intertechnician or inter-technician or intratechnician or intra-technician or interexaminer or inter-examiner or intraexaminer or intra-examiner or interassay or inter-assay or intraassay or intra-assay or interindividual or inter-individual or intraindividual or intra-individual or interparticipant or inter-participant or intraparticipant or intra-participant or kappa*2 or "k" or repeatab*).ti,ab,kw. 912454 Advanced

22 ((replicab* or repeated) and (measure* or findings or result or results or test or tests)).ti,ab,kw. 211668 Advanced

23 (generaliza* or generalisa* or concondance).ti,ab,kw. 32900 Advanced

24 (intraclass and correlation*).ti,ab,kw. 19773 Advanced

25 (discriminative or "known group*" or factor analysis or factor analyses or dimension* or subscale*).ti,ab,kw. 502827 Advanced

26 (multitrait and scaling and (analysis or analyses)).ti,ab,kw. 135 Advanced

27 (item discriminant or interscale correlation* or error or errors or "individual variability").ti,ab,kw. 252069 Advanced

28 (variability and (analysis or values)).ti,ab,kw. 100255 Advanced

29 (uncertainty and (measure* or measuring)).ti,ab,kw. 16776 Advanced

30 ("standard error of measurement" or sensitiv* or responsive*).ti,ab,kw. 1396589 Advanced

31 ((minimal* or clinical*) and (important or significant or detectable) and (change or difference)).ti,ab,kw. 272285 Advanced

32 (small* and (real or detectable) and (change or difference)).ti,ab,kw. 7768 Advanced

33 (meaningful change or ((ceiling or floor) adj3 effect*) or "item response model" or irt or rasch or "differential item functioning" or dif or "computer adaptive testing" or "item bank" or "cross-cultural*").ti,ab,kw. 25046 Advanced

34 or/1-33 5726708 Advanced

35 "glasgow outcome".mp. or gos.tw. or gose.tw. [mp=title, abstract, heading word, drug trade name, original title, device manufacturer, drug manufacturer, device trade name, keyword, floating subheading] 7539 Advanced

36 34 and 35 3643 Advanced

37 (tbi or mtbi or "traumatic brain").mp. or exp head injuries/ or exp craniocerebral trauma/ or concuss*.mp. [mp=title, abstract, heading word, drug trade name, original title, device manufacturer, drug manufacturer, device trade name, keyword, floating subheading] 226165 Advanced

38 ((head or cranial or skull) adj2 (trauma* or injur* or fractur*)).mp. [mp=title, abstract, heading word, drug trade name, original title, device manufacturer, drug manufacturer, device trade name, keyword, floating subheading] 53601 Advanced

39 36 and (37 or 38) 1836 Advanced

40 limit 39 to english language 1751 Advanced

41 40 not (letter or note or conference abstract).pt. 1321 Advanced

42 exp brain injury/ 138441 Advanced

43 36 and 42 1583 Advanced

44 limit 43 to (human and english language) 1423 Advanced

45 44 not (letter or note or conference abstract).pt. 1118 Advanced

85 additional foreign language.

PsycINFO 1967 to October Week 2 2016

1 (instrumentation or methods).sh. 0 Advanced

2 exp intermethod comparison/ or exp data collection method/ or exp validation study/ or exp feasibility study/ or exp pilot study/ or exp psychometry/ or exp reproducibility/ or exp observer variation/ or exp discriminant analysis/ or exp validity/ 0 Advanced

3 exp psychometrics/ 44874 Advanced

4 psychometr*.ti,ab,kw. 42634 Advanced

5 (clinimetr* or clinometr*).ti,ab,kw. 197 Advanced

6 exp "outcome assessment (health care)"/ 0 Advanced

7 outcome assessment*.ti,ab,kw. 1048 Advanced

8 outcome measure*.ti,ab,kw. 30589 Advanced

9 exp observer variation/ 0 Advanced

10 (observer adj2 variation*).ti,ab,kw. 28 Advanced

11 exp health status indicators/ 0 Advanced

12 exp "reproducibility of results"/ 0 Advanced

13 reproducib*.ti,ab,kw. 4202 Advanced

14 exp discriminant analysis/ or (discriminant* adj2 analys*).mp. [mp=title, abstract, heading word, table of contents, key concepts, original title, tests & measures] 7550 Advanced

15 (reliab* or unreliab* or valid* or coefficient or homogeneity or homogeneous or "internal* consisten*").ti,ab,kw. 271094 Advanced

16 (cronbach* and (alpha or alphas)).ti,ab,kw. 8922 Advanced

17 (item and (correlat* or selection* or reduction*)).ti,ab,kw. 18201 Advanced

18 (agreement or precision or imprecision or "precise values" or test-retest).ti,ab,kw. 54985 Advanced

19 (test and retest).ti,ab,kw. 14888 Advanced

20 reliab*.mp. and (test* or retest*).ti,ab,kw. [mp=title, abstract, heading word, table of contents, key concepts, original title, tests & measures] 49604 Advanced

21 (stability or interrater or inter-rater or intrarater or intra-rater or intertester or inter-tester or intratester or intra-tester or interobserver or inter-observer or intraobserver or intra-observer or intertechnician or inter-technician or intratechnician or intra-technician or interexaminer or inter-examiner or intraexaminer or intra-examiner or interassay or inter-assay or intraassay or intra-assay or interindividual or inter-individual or intraindividual or intra-individual or interparticipant or inter-participant or intraparticipant or intra-participant or kappa*2 or "k" or repeatab*).ti,ab,kw. 95856 Advanced

22 ((replicab* or repeated) and (measure* or findings or result or results or test or tests)).ti,ab,kw. 40911 Advanced

23 (generaliza* or generalisa* or concondance).ti,ab,kw. 30375 Advanced

24 (intraclass and correlation*).ti,ab,kw. 3230 Advanced

25 (discriminative or "known group*" or factor analysis or factor analyses or dimension* or subscale*).ti,ab,kw. 204953 Advanced

26 (multitrait and scaling and (analysis or analyses)).ti,ab,kw. 56 Advanced

27 (item discriminant or interscale correlation* or error or errors or "individual variability").ti,ab,kw. 85724 Advanced

28 (variability and (analysis or values)).ti,ab,kw. 9977 Advanced

29 (uncertainty and (measure* or measuring)).ti,ab,kw. 3573 Advanced

30 ("standard error of measurement" or sensitiv* or responsive*).ti,ab,kw. 157441 Advanced

31 ((minimal* or clinical*) and (important or significant or detectable) and (change or difference)).ti,ab,kw. 20360 Advanced

32 (small* and (real or detectable) and (change or difference)).ti,ab,kw. 662 Advanced

33 (meaningful change or ((ceiling or floor) adj3 effect*) or "item response model" or irt or rasch or "differential item functioning" or dif or "computer adaptive testing" or "item bank" or "cross-cultural*").ti,ab,kw. 33491 Advanced

34 or/1-33 849918 Advanced

35 "glasgow outcome".mp. or gos.tw. or gose.tw. [mp=title, abstract, heading word, table of contents, key concepts, original title, tests & measures] 1008 Advanced

36 34 and 35 391 Advanced

37 limit 36 to all journals 371

CINAHL

S3 S1 AND S2 Search modes - Boolean/Phrase Interface - EBSCOhost Research Databases

Search Screen - Advanced Search

Database - CINAHL with Full Text 221

S2 "glasgow outcome" OR gos OR gose Search modes - Boolean/Phrase Interface - EBSCOhost Research Databases

Search Screen - Advanced Search

Database - CINAHL with Full Text 488

S1 (MH "Psychometrics") or ( TI psychometr* or AB psychometr* ) or ( TI clinimetr* or AB clinimetr* ) or ( TI clinometr* OR AB clinometr* ) or (MH "Outcome Assessment") or ( TI outcome assessment or AB outcome assessment ) or ( TI outcome measure* or AB outcome measure* ) or (MH "Health Status Indicators") or (MH "Reproducibility of Results") or (MH "Discriminant Analysis") or ( ( TI reproducib* or AB reproducib* ) or ( TI reliab* or AB reliab* ) or ( TI unreliab* or AB unreliab* ) ) or ( ( TI valid* or AB valid* ) or ( TI coefficient or AB coefficient ) or ( TI homogeneity or AB homogeneity ) ) or ( TI homogeneous or AB homogeneous ) or ( TI "coefficient of variation" or AB "coefficient of variation" ) or ( TI "internal consistency" or AB "internal consistency" ) or (MH "Internal Consistency+") or (MH "Reliability+") or (MH "Measurement Error+") or (MH "Content Validity+") or "hypothesis testing" or "structural validity" or "cross-cultural validity" or (MH "Criterion-Related Validity+") or "responsiveness" or "interpretability" or ( TI reliab* or AB reliab* ) and ( (TI test or AB test) OR (TI retest or AB retest) ) or ( TI stability or AB stability ) or ( TI interrater or AB interrater ) or ( TI inter-rater or AB inter-rater ) or ( TI intrarater or AB intrarater ) or ( TI intra-rater or AB intrarater ) or ( TI intertester or AB intertester) or (TI inter-tester or AB inter-tester) or ( TI intratester or AB intratester) or ( TI intra-tester or AB intra-tester) or ( TI interobserver or AB interobserver) or (TI inter-observer or AB inter-observer ) or ( TI intraobserver or AB intraobserver) or ( TI intra-observer or AB intra-observer) or ( TI intertechnician or AB intertechnician) or (TI inter-technician or AB inter-technician) or ( TI intratechnician or AB intratechnician ) or ( TI intra-technician or AB intra-technician ) or ( TI interexaminer or AB interexaminer ) or (TI inter-examiner or AB inter-examiner) or (TI intraexaminer or AB intraexaminer ) OR (TI intra-examiner or AB intra-examiner ) or (TI intra-examiner or AB intraexaminer ) or (TI interassay or AB interassay ) or ( TI inter-assay or AB inter-assay ) or ( TI intraassay or AB intraassay) or ( TI intra-assay or AB intra-assay ) or (TI interindividual or AB interindividual) or (TI inter-individual or AB inter-individual) OR (TI intraindividual or AB intraindividual) or (TI intra-individual or AB intra-individual) or (TI interparticipant or AB interparticipant) or (TI inter-participant or AB inter-participant ) or (TI intraparticipant or AB intraparticipant) or (TI intra-participant or AB intra-participant ) or (TI kappa or AB kappa) or (TI kappa's or AB kappa's ) or (TI kappas or AB kappas) or (TI repeatab* or AB repeatab*) or ( TI responsive* or AB responsive* ) or ( TI interpretab* or AB interpretab* ) Search modes - Boolean

Scopus

(TITLE-ABS-KEY("glasgow outcome*" OR gos OR gose) AND (valid* OR psychomet* OR clinimet* OR clinomet* OR outcome* OR reproducib* OR variation* OR discriminant or reliab* OR unreliab* OR coefficient OR homogen* OR consisten* OR inter* OR intra* OR concordan* OR generali* OR "item response" OR change OR important OR ceiling OR floor OR dif OR "differential item" OR "item bank" OR detectable) AND (brain* OR tbi* OR mtbi* OR concuss* OR crani* OR cerebral* OR head* OR trauma* OR injur* OR fracture*)) AND NOT (PMID(1* OR 2* OR 3* OR 4* OR 5* OR 6* OR 7* OR 8* OR 9*)) 1250
