# Supplementary material for: An evidence-based methodology for systematic evaluation of clinical outcome assessment measures for traumatic brain injury
Source: PLoS One. 2020 Dec 14;15(12):e0242811. doi: 10.1371/journal.pone.0242811 (PMC7735614; doi:10.1371/journal.pone.0242811)
Supplement: S4 File — The RPQ is a widely-used patient-reported measure of symptom severity following mild TBI. In this study, we assessed the Rivermead to address the following evidence question: In adult patients with mTBI of subacute (<6mo) duration, for the purpose of (1) detecting PCS, (2) stratifying sub-types, or (3) monitoring the resolution or progression of PCS, are PCS adequately measured by the English version of the patient-reported RPQ? Seventy-nine full-text articles were reviewed by two independent reviewers and four qualified for EB-COP review. The two most common reasons for exclusion were study design (e.g., conference abstracts, review articles, books) and lack of relevance to the evidence question. Results indicated that there is paucity of relevant, high-quality evidence addressing the performance of the RPQ within the three COUs assessed. As such, the RPQ was not recommended for these applications. (PDF) [file pone.0242811.s004.pdf]

The Validity of the Rivermead Post-Concussion Questionnaire (RPQ) in the Detection and Monitoring of Post-Concussive Symptoms

Andrea Christoforou, PhD, PT[1], Stephanie Agtarap, PhD[2], Shannon Merillat, MLIS[3], Patricia Erwin, MLS[4], Murray Stein, MD[2], and Joseph Giacino, PhD[1]

[1]Spaulding Rehabilitation Hospital & Harvard Medical School, Boston, MA; [2]University of California, San Diego, CA; [3]American Academy of Neurology, Minneapolis, MN; [4]Mayo Clinic, Rochester, MN

Objective

- To determine the strength of the evidence supporting use of the Rivermead Post-Concussion Questionnaire (RPQ) for detection of symptoms of post-concussive syndrome (PCS), stratification of PCS sub-types and monitoring of natural history changes.

Background

- PCS symptoms commonly impact physical (eg, headache, double vision), emotional (eg, depression, irritability) and cognitive (eg, concentration and memory) functions following mild traumatic brain injury (mTBI (ie, GCS>12, confusion, loss of consciousness <30 mins, post-traumatic amnesia <24 hours).
- RPQ is a widely-used TBI clinical outcome assessment measure (COA) that assesses the presence and severity of PCS symptoms in the past 24 hours relative to pre-injury levels.
- RPQ is an NINDS common data element (CDE) for use in research concerning mTBI and sports-related concussion, however, its psychometric integrity and factor structure have not been evaluated for specific "Contexts of Use (COUs)".

Methods

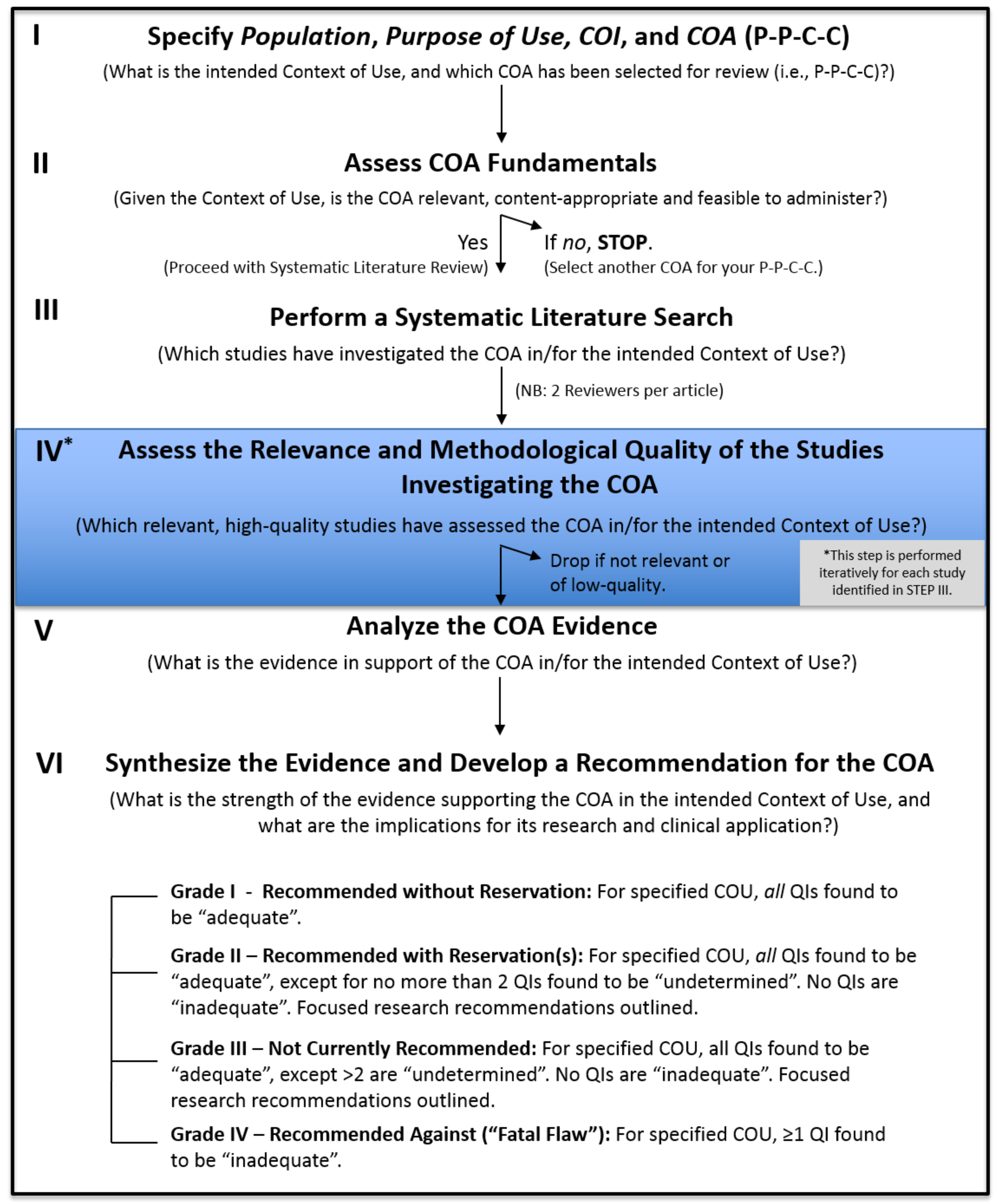

COI, "Concept of Interest" or trait being measured; COU, the intended population and purpose of the COA; PoU, "Purpose of Use"; QI, quality indicator or psychometric property

The RPQ was evaluated using the Evidence-Based Clinical Outcome Assessment Platform ("EB-COP") (left), which relies on:

- Pre-determined quality indicators for specific PoUs;
- High-quality (Level I and II) evidence;
- Transparent and standardized criteria for establishing recommendations.

I. Evidence Question (P-P-C-C)

In adult patients with mTBI of subacute (<6mo) duration, for the purpose of (1) detecting PCS, (2) stratifying sub-types, or (3) monitoring the resolution or progression of PCS, are PCS adequately measured by the English version of the patient-reported RPQ?

Results

II. Are the fundamental QIs (ie, documented development; specification of population, COI and PoU; content and face validity; feasibility; data quality/missingness) adequate?

- Evidence or support for each fundamental QI provided in the original description by King et al., 1995, namely: It is an easy to use 16-item questionnaire of most commonly cited symptoms following mTBI with 5 symptoms ratings (0 – no experience at all; 1- no more of a problem; 2 – a mild problem; 3 – a moderate problem; 4 – a severe problem) and a total score that sums each response, excluding 1's.

III. Which studies have investigated the RPQ within the selected POUs?

- 437 abstracts drawn from OVID Medline, Embase, PsycINFO, and SCOPUS qualified for full-text review. One additional study was identified via ResearchGate.

IV. How many high-quality studies have assessed the RPQ in/for the selected COUs?

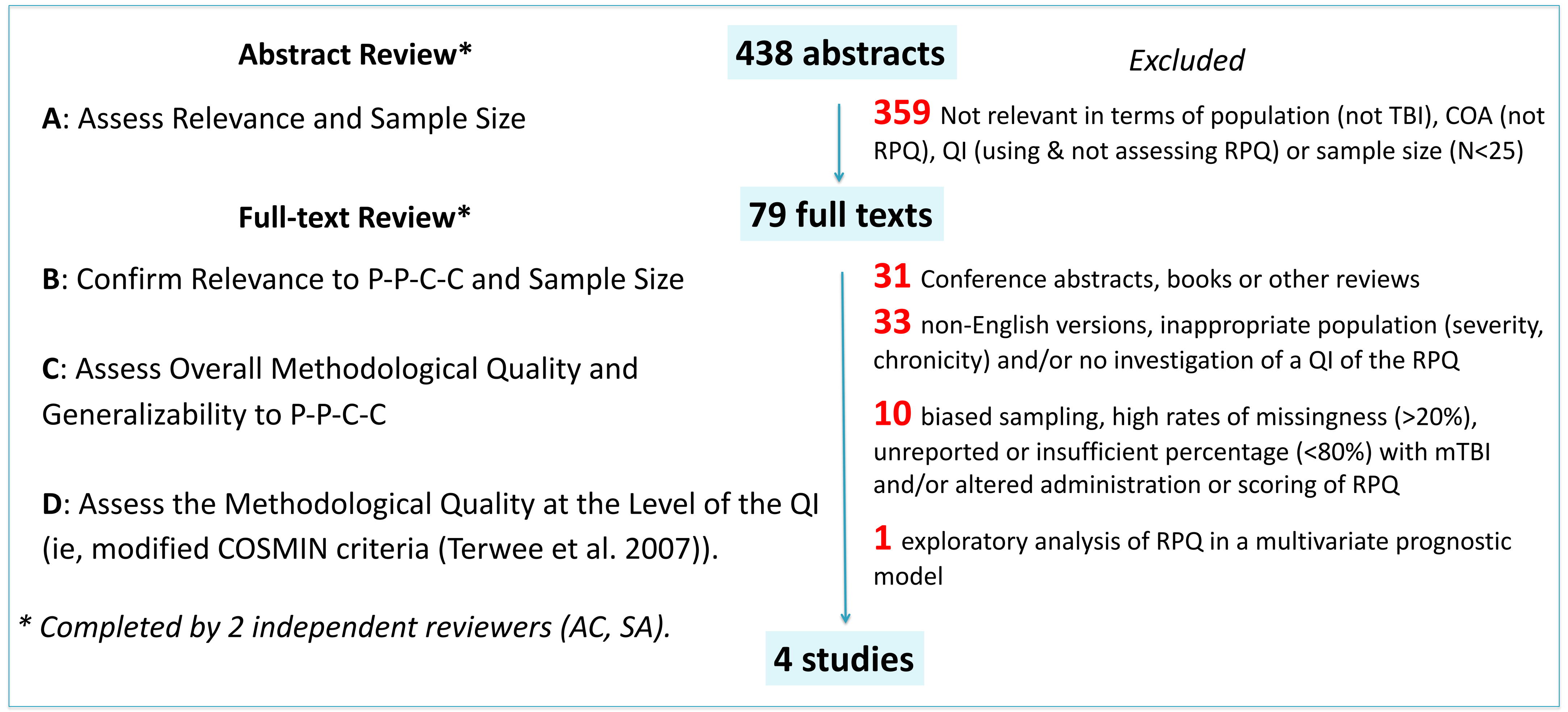

V. What is the evidence in support of the RPQ within each COU?

Study	Population (age, severity, chronicity)	Sample Size (% male)	QI and Results ([1,2,3] refer to relevant PoUs from Step I)	EB-COP Cut-off for Adequacy	EB-COP Rating
de Guise et al. 2016.	adult (mean 39.3yo), mTBI, 2-12 days and 22-137 days post injury	47 (48.9%)	prognostic validity w/ cut-off[2]: RPQ≥35 identifies individuals with moderate-severe limitations (on Mayo-Portland Adaptability Inventory-4 (MPAI-4)) w/ 90% sensitivity, 60% specificity (ROC AUC: 0.777 [95% CI: 0.593-0.961])	sensitivity>0.80, specificity>0.60; ROC≥AUC0.80	Mixed/Undetermined
King et al. 1995.	adult (mean 31yo), mTBI, 7-10 days post injury	41 (54%)	test-restest reliability (cross-sectional)[1,2,3]: Spearman's=0.90	≥0.70	Adequate
King et al. 1996.	adult (mean 33yo), mTBI, RPQ at 7-10 days and 3 months post-injury	50 (46%)	score variability and floor/ceiling effects[1,3]: >80% of sample with score <31 for both time-points; ceiling effects=36% at 3 months	Floor/Ceiling Effects <15%	Adequate (7-10days); Inadequate (3 months)
King et al. 1999.	adult (mean 32yo), mTBI, RPQ at 7-10 days and 6 months post-injury	66 (65%)	score variability and floor/ceiling effects[1,3]: >80% of sample with score <31 for both time-points; ceiling effects=19% at 6 months	Floor/Ceiling Effects <15%	Adequate (7-10days); Inadequate (6 months)

Grades & Recommendation

VI. What is the strength of the evidence for use of the RPQ within each COU?

- Eight mandatory QIs for (1) detecting PCS; 8 for (2) stratifying PCS subtypes and 10 for (3) monitoring change in PCS were undetermined (but not "inadequate") [within the first ~2 weeks post-injury for (1) and (3)]

Conclusion: RPQ is **"Not Currently Recommended" (Grade III)** for use in adult patients with mTBI of (early) subacute duration.

- One mandatory QI required for 1) detecting PCS and 2) monitoring changes at 3 to 6 months post-injury effects was "inadequate".

Conclusion: RPQ is **"Recommended Against" (Grade IV)** for use in adult patients with mTBI who are 3 and 6 months post-injury due to ceiling effects.

Recommendation: Additional research is needed in the following PoU-specific QIs:

- Criterion or convergent validity[1,2,3], diagnostic validity/cut-off score[2], external responsiveness[3], inter-rater reliability (cross-sectional[1,2,3] & longitudinal[3]), internal consistency[1,2,3], internal construct validity[1,3], minimum clinically important difference[3], normative values[1], test-retest reliability (longitudinal[3]), unidimensionality[1,2,3]

Discussion

- There is a paucity of relevant, high-quality evidence in support of the RPQ within specific COUs, calling into question its selection as a mTBI CDE.
- Claims about the RPQ's factor structure/unidimensionality are not adequately supported due to low quality evidence.
- Some well-designed RPQ studies may have been disqualified for not meeting EB-COP requirements for study inclusion or mandatory criteria even if other methodologic aspects of the study were sound.

Key References/Acknowledgement

- ➢ American Academy of Neurology (AAN). 2011 Ed. St. Paul, MN: The AAN American Academy of Neurology.
- ➢ deGuise et al. 2016. *Applied Neuropsychology*, Adult 23(3):213-222.
- ➢ King et al. 1995. *Journal of Neurology*, 242:587-592.
- ➢ King et al. 1996. *Journal of Neurology, Neurosurgery & Psychiatry*, 61(1): 75-81.
- ➢ King et al. 1999. *British Journal of Clinical Psychology*, 38(Pt 1): 15-25.
- ➢ Terwee et al. 2007. *Journal of Clinical Epidemiology*, 60: 34-42.

This project was funded by the Department of Defense (Award: W81XWH-14-2-0176).

Contacts: achristoforou@partners.org, sagtarap@UCSD.edu
